# NUP43 promotes PD-L1/nPD-L1/PD-L1 feedback loop via TM4SF1/JAK/STAT3 pathway in colorectal cancer progression and metastatsis

**DOI:** 10.1038/s41420-024-02025-z

**Published:** 2024-05-18

**Authors:** Fan Wu, Guoqiang Sun, Yongjun Nai, Xuesong Shi, Yong Ma, Hongyong Cao

**Affiliations:** 1 Department of General Surgery, Nanjing First Hospital, Nanjing Medical University, Nanjing, Jiangsu China; 2 Department of Liver Surgery, Liver Cancer Institute, Zhongshan Hospital, and Key Laboratory of Carcinogenesis and Cancer Invasion (Ministry of Education), Fudan University, Shanghai, China

**Keywords:** Colorectal cancer, Metastasis

## Abstract

Programmed cell death-ligand 1 (PD-L1) has a significant role in tumor progression and metastasis, facilitating tumor cell evasion from immune surveillance. PD-L1 can be detected in the tumor cell nucleus and exert an oncogenic effect by nuclear translocation. Colorectal cancer (CRC) progression and liver metastasis (CCLM) are among the most lethal diseases worldwide, but the mechanism of PD-L1 nuclear translocation in CRC and CCLM remains to be fully understood. In this study, using CRISPR-Cas9-based genome-wide screening combined with RNA-seq, we found that the oncogenic factor NUP43 impacted the process of PD-L1 nuclear translocation by regulating the expression level of the PD-L1 chaperone protein IPO5. Subsequent investigation revealed that this process could stimulate the expression of tumor-promoting factor TM4SF1 and further activate the JAK/STAT3 signaling pathway, which ultimately enhanced the transcription of PD-L1, thus establishing a PD-L1-nPD-L1-PD-L1 feedback loop that ultimately promoted CRC progression and CCLM. In conclusion, our study reveals a novel role for nPD-L1 in CRC, identifies the PD-L1-nPD-L1-PD-L1 feedback loop in CRC, and provides a therapeutic strategy for CRC patients.

## Introduction

Colorectal cancer (CRC) is the third most frequently diagnosed cancer worldwide and the second most common cause of cancer-related deaths [1]. Aside from smoking, an unhealthy diet, excessive alcohol consumption, lack of physical activity, and being overweight, genetic and epigenetic alterations can also contribute to the development and advancement of CRC. These alterations include mutations that enhance the function of p53 and APC genes, as well as the increased activity of β-catenin and MAPK [2–4]. While there has been some improvement in the overall survival rate of CRC in recent decades, the specific molecular pathways behind its development are still not well understood. Furthermore, similar to the majority of solid tumors, metastatic progression is a significant contributor to mortality in CRC [5, 6]. About 20% of colorectal cancer patients develop metastasis, while 50% of patients with localized disease eventually develop metastasis, with the liver being the main site of colorectal cancer metastasis [7–9]. During the last two decades, the outlook for patients with metastatic colorectal cancer (mCRC) has greatly improved due to the development of more efficient treatments. In most cases, mCRC remains a difficult disease to treat, especially colorectal cancer liver metastases (CCLM). Undetermined specific genes and mechanisms of CCLM are a major reason for poor clinical treatment [1]. Hence, it is necessary to fully understand the biological mechanisms of CRC and CCLM and to propose more effective therapeutic interventions.

Programmed cell death-ligand 1 (PD-L1, gene symbol: CD274) is extensively expressed in different malignant tumors, serving as a significant component of the B7 protein family. It enhances malignant tumor immune escape by binding to programmed cell death-1 (PD-1) receptors on immune cells [10–13]. While immunotherapies that target the PD-L1/PD-1 checkpoint have shown promising results in enhancing immune cell responses and treating advanced malignant tumors, only a minority of patients experience long-lasting benefits from these therapies. The majority of patients do not respond to blocking the PD-1/PD-L1 axis [14–19]. Mounting evidence indicates that PD-L1, functioning as a cell surface receptor, can transmit internal signals and significantly contribute to the initiation, progression, and resistance to drugs in cancers. In CRC, overexpression of PD-L1 can increase the expression of HMGA1, which in turn activates the PI3K/Akt and MEK/ERK pathways. This ultimately leads to an enhancement in the tumorigenicity of CRC cells [20]. Knocking down PD-L1 in human lung cancer cell lines HCC827 and PC9 leads to a substantial reduction in tumor cell growth and triggers cell death [21]. Research on intrahepatic cholangiocarcinoma (ICC) has revealed that the PD-L1 protein contained in myofibroblasts can enhance the activation of hepatic stellate cells (HSC) and increase the formation of ICC by stabilizing the TGF-β receptor I (TβRI) [22]. This process regulates the tumor microenvironment and tumor growth through mechanisms that are not related to immunosuppression. PD-L1 in bladder cancer (BC) facilitates mTOR signaling and autophagy, which contribute to the development of resistance to the widely employed BC chemotherapeutic drugs cisplatin and gemcitabine, as well as the mTORC1 inhibitor rapamycin [23]. Furthermore, current research has discovered that PD-L1 can function as a catalyst for cancer development and directly control the growth of tumors that are not influenced by the immune system [24, 25]. Nevertheless, the mechanisms by which PD-L1 transmits internal signals and the specific signalosomes implicated have yet to be fully understood.

Previous research has commonly held the belief that PD-L1 is predominantly localized in the cell membrane and cytoplasm. Nevertheless, other studies have shown that PD-L1 has the ability to translocate to the cell nucleus and has a significant impact on a wide range of cellular activities. Research has demonstrated that PD-L1 nuclear expression can be identified in various types of cancer, such as esophageal cancer, colorectal cancer, and prostate cancer. This expression is strongly associated with the extent of tumor invasion and reduced patient survival. These findings indicate that nuclear PD-L1(nPD-L1) could serve as a promising prognostic biomarker for individuals with cancer [26, 27]. Recent findings indicate that nPD-L1 has a role in the regulation of sister chromatid cohesion and maintenance of genome stability in cancer cells [28]. Furthermore, it has the ability to control the activation of immune-related genes, influence the effectiveness of anti-PD-1 immunotherapy, and facilitate the programmed cell death of tumor cells through the regulation of GSDMC/caspase-8 [29, 30]. Nevertheless, the precise nuclear entry mechanism of PD-L1 and the role of nPD-L1 in CRC remain uncertain.

The CRISPR/Cas9 system is an RNA-dependent adaptive immunity system that has been evolved by prokaryotes to counteract external viral assaults [31]. Several studies have shown that the CRISPR/Cas9 system possesses accurate and effective gene editing skills, allowing it to address a range of human diseases, such as cancer, by affecting human genes [32]. In addition to its status as a potent and adaptable platform for genome engineering, CRISPR/Cas9 is also a straightforward and efficient technique. CRISPR/Cas9 system variants incorporating transcriptional repressors or activators have the potential to enhance the efficacy of current treatments by enabling robust transcriptional repression or activation of specific genes [33]. This study involved the construction of a mouse model to simulate CCLM. We then performed a comprehensive CRISPR/Cas9 screening to identify the crucial regulatory elements that influence CCLM. We provide comprehensive data from the screening, enumerating relevant genes that promote or inhibit CCLM. While certain genes have been linked to the development of tumors, their specific function in the process of metastatic colonization remains poorly understood. Through screening for differential genes, we have discovered that Nucleoporin 43 (NUP43) potentially plays a role in CCLM. Our study discovered that NUP43 has the ability to increase the expression of Importin 5 (IPO5). IPO5, in turn, acts as a binding partner for intracellular PD-L1, facilitating the movement of PD-L1 from the cytoplasm to the nucleus in CRC. This process leads to the buildup of PD-L1 in the nucleus of CRC cells. This process involves the stimulation of the transcription and translation of Transmembrane 4 L Six Family Member 1 (TM4SF1), which in turn activates the JAK/STAT3 signaling pathway to regulate the synthesis of PD-L1 mRNA. This leads to the formation of a PD-L1-nPD-L- PD-L1 feedback loop, which results in an overall increase of PD-L1 expression in CRC cells and contributes to CRC incidence, progression, and metastasis. Collectively, we have discovered a new cancer-causing function of nPD-L1 and identified a significant target for therapeutic intervention in the treatment of CRC.

## Results

### Genome-wide CRISPR/Cas9-based screening for CCLM-regulated genes

**Fig. 1:**
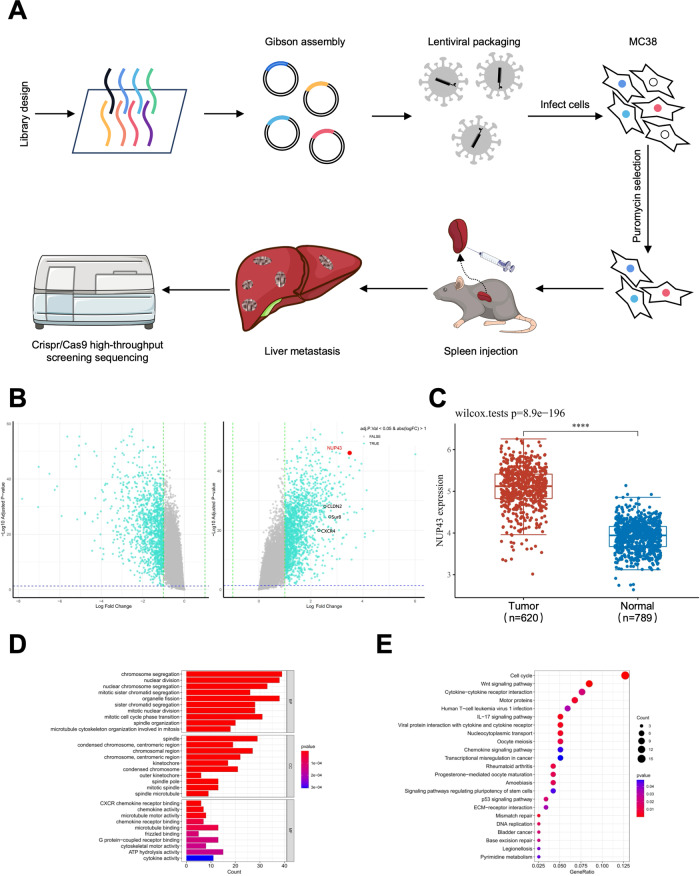
Genome-wide CRISPR/Cas9 screen analysis reveals that NUP43 plays a role in promoting oncogenesis and liver metastasis in CRC. **A** Genome-wide CRISPR screen has identified genes that might control the formation and progression of CRC. **B** Volcano plot displaying the genes that exhibit differential expression. The left side is characterized by positivity, whereas the right side is characterized by negativity. The identification of NUP43 is indicated by the color red. **C** Analysis of NUP43 expression in both healthy tissues and CRC tissues using data from the TCGA database. **D** GO analysis of the negative group. **E** KEGG pathway analysis of the negative group.

To identify genes that facilitate the growth and liver metastasis of CRC, we conducted a whole-genome screen based on CRISPR-Cas9 in the mouse colorectal cancer cell line MC38. The cells were infected with lentivirus containing guide RNA (sgRNA) and treated with puromycin. After selecting factors and amplifying the infected cells, the expanded MC38 cells were used to construct a mouse CCLM model. CRISPR/Cas9 high-throughput screening technology was ultimately employed to examine the acquired mice liver metastases and identify the crucial regulatory elements responsible for CCLM (Fig. 1A). We used volcano plots to visually represent the genes that showed statistically significant differences in the results of the CRISPR negative screen (Fig. 1B). The GO analysis revealed that the most enriched functional categories of differentially expressed genes predominantly involved nuclear division, sister chromatid separation, and encouragement of cell proliferation. Furthermore, they participated in microtubule-dependent locomotion and intracellular protein trafficking (Fig. 1D). The KEGG analysis revealed that the differentially expressed genes mostly impact nucleocytoplasmic transport, cell cycle regulation, DNA replication, and other related processes (Fig. 1E). Differential genes that affect CCLM identified by CRISPR/Cas9 screen technology can be targeted for further research.

### Elevated NUP43 expression in CRC tissues is associated with high nPD-L1 and indicates a worse clinical prognosis

**Fig. 2:**
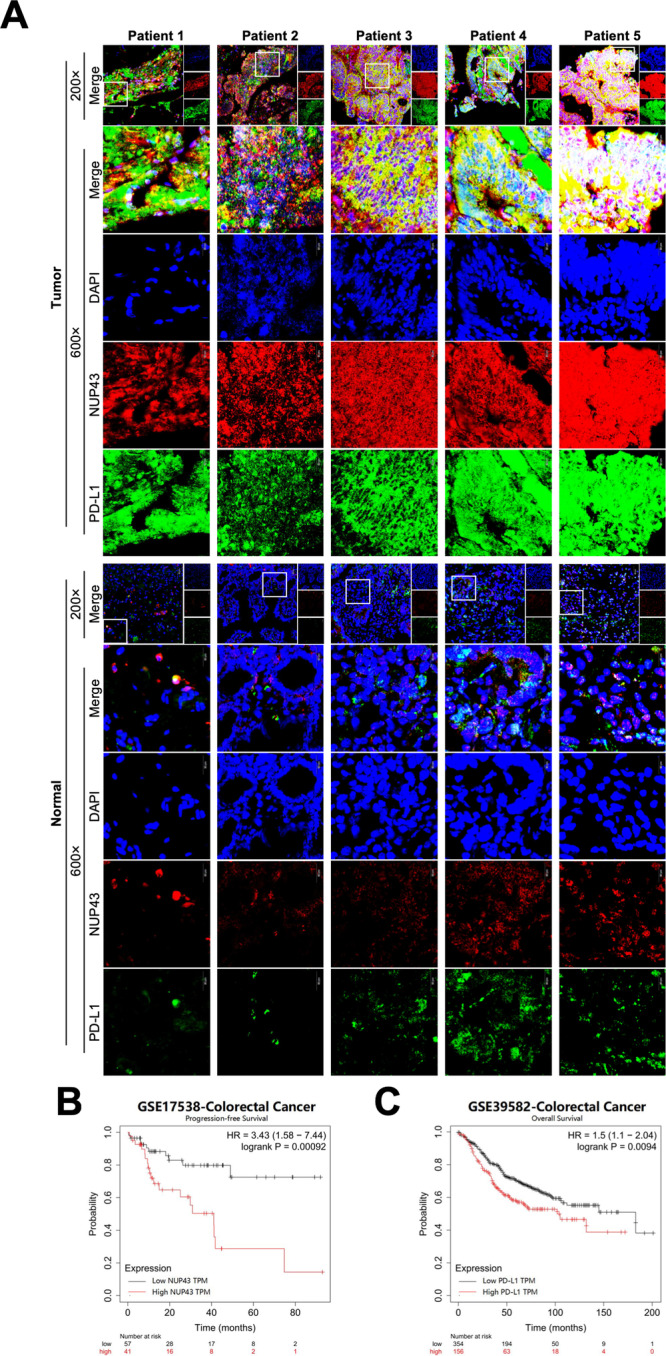
Analysis of NUP43 and PD-L1 expression in human tissues and their influence on prognosis. **A** Immunofluorescence analysis was performed to determine the expression of NUP43 (red) and PD-L1 (green) in cancerous tissue and the comparable surrounding tissue of five patients with CRC. **B**, **C** Analysis of survival time using the TCGA and GEO databases.

Utilizing CRISPR/Cas9 technology, we conducted a screening of differential genes in the negative group to identify potential targets that facilitate the onset, progression, and liver metastasis of CRC. Our first findings suggest a potential tight association between the NUP43 gene and these processes (Fig. 1B). Nevertheless, this result was derived from a mouse model. To confirm the expression of NUP43 in human CRC tissues, we utilized the TCGA database to do a comparative analysis of gene expression between CRC samples and normal samples. The findings revealed a considerable upregulation of NUP43 in CRC tissues compared to normal tissues (Fig. 1C). Furthermore, we employed immunofluorescence to identify malignant and paracancerous tissues in five individuals diagnosed with CRC. Considering the widespread usage of PD-L1/PD-1 as a prominent target in clinical practice, we conducted NUP43 and PD-L1 double fluorescence labeling. The results revealed a significantly higher expression of NUP43 in cancer tissues compared to neighboring tissues. Simultaneously, we noted a consistent pattern for PD-L1, suggesting a correlation between the two factors(Fig. 2A). Upon further examination of the findings, we discovered that in cancerous tissues exhibiting elevated NUP43 expression, both the amount of PD-L1 expression and its co-localization with the nucleus were dramatically increased. It was hypothesized that NUP43 may influence the geographical distribution of PD-L1. Based on the Kaplan–Meier survival curve, patients with CRC who had elevated levels of NUP43 or PD-L1 showed a worse prognosis (Fig. 2B, C).

### NUP43 facilitates CRC development and liver metastasis both in vitro and in vivo

**Fig. 3:**
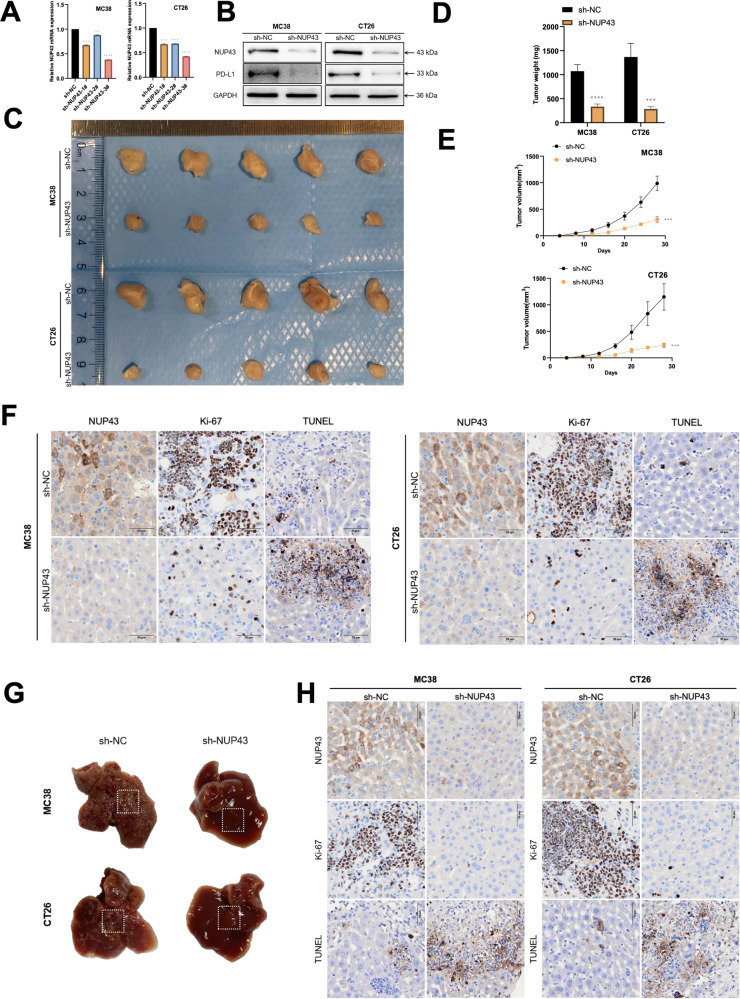
Suppression of NUP43 hampers the progression of CRC and liver metastasis in vivo. **A**, **B** Three shRNAs (sh-NUP43-1#, sh-NUP43-2#, and sh-NUP43-3#) were designed to silence CRC cells (MC38 and CT26) and verified by qRT-PCR and Western blotting. We used sh-NUP43-3# to downregulate NUP43. **C** Illustrative photos of subcutaneous tumors from both the sh-NC and sh-NUP43 groups. **D**, **E** Statistics of weight (**D**) and volume (**E**) of subcutaneous tumors in each group. **F** Immunohistochemical findings of NUP43, Ki-67, and TUNEL expression were seen in each group. **G** Illustrative visuals of liver metastases in both the sh-NC and sh-NUP43 groups. **H** Immunohistochemical findings of NUP43, Ki-67, and TUNEL expression were seen in each group. *P < 0.05; **P < 0.01; ***P < 0.001; ****P < 0.0001.

Hence, it is imperative to investigate the significance of NUP43 in CRC. We designed three specific shRNAs targeting NUP43 in MC38 and CT26 cells to induce gene silencing. We assessed the effectiveness of gene suppression by using qRT-PCR and confirmed the reduction of NUP43 expression at the protein level by using Western blotting. We selected sh-NUP43-3# as the second knockdown sequence (Fig. 3A, B). In this study, we employed the C57/B6 mouse subcutaneous tumor-bearing model to assess the impact of NUP43 in vivo. The findings indicated that the downregulation of NUP43 led to a notable decrease in both the size and weight of tumors. The immunohistochemical analysis revealed a significant decrease in the expression of Ki-67 (Fig. 3C–F). Meanwhile, we administered MC38 and CT26 cells that were transfected with sh-NUP43 and sh-NC, respectively, into the spleens of C57BL/6 mice. In comparison to the sh-NC group, the suppression of NUP43 in the sh-NUP43 group markedly hindered the CCLM. The immunohistochemical findings demonstrated a decrease in Ki-67 expression (Fig. 3G, H).

**Fig. 4:**
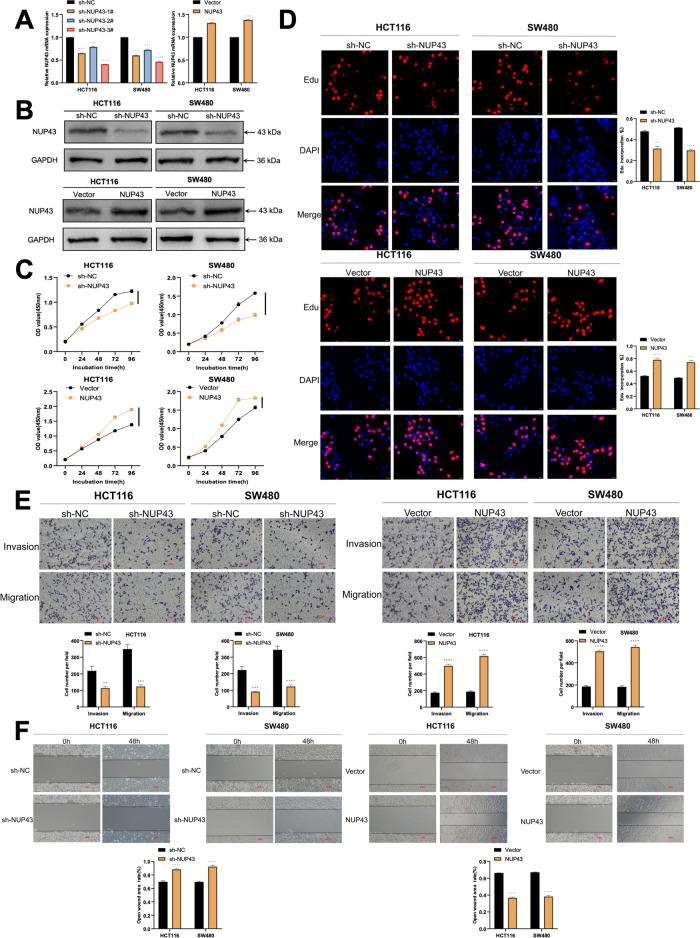
NUP43 enhances the growth and infiltration of CRC cells. **A**, **B** Three shRNAs (sh-NUP43-1#, sh-NUP43-2#, and sh-NUP43-3#) were designed to silence CRC cells (HCT116 and SW480) and verified by qRT-PCR and Western blotting. We used sh-NUP43-3# to downregulate NUP43. NUP43 overexpression was verified in CRC cells (HCT116 and SW480) by qRT-PCR and Western blotting. **C** The growth curve of cells transfected with sh-NUP43/NUP43 was drawn according to the CCK-8 method. The upper figure shows the cell proliferation results after NUP43 knockdown, and the lower figure shows the cell proliferation results after NUP43 overexpression. **D** The EdU assay is used to assess the proliferation capacity of CRC cells that have been transfected with sh-NUP43/NUP43. The left panel depicts cellular proliferation following the manipulation of NUP43 through knockdown or overexpression. The findings of the cell counting analysis are displayed in the right section. **E** Transwell test was used to assess the invasive and metastatic properties of CRC cells that were transfected with sh-NUP43/NUP43. The upper panel displays the outcomes of cell invasion and metastasis following the manipulation of NUP43 through knockdown or overexpression. The lower panel displays the findings of migration and metastatic cell count assessments. **F** A scratch experiment was conducted to assess the invasive capacity of CRC cells that were transfected with sh-NUP43/NUP43. *P < 0.05; **P < 0.01; ***P < 0.001; ****P < 0.0001.

To better understand the function of NUP43 in CRC cells and to determine its association with cell proliferation and metastasis, we validated the effect of shRNA targeting NUP43 in HCT116 and SW480 cells by using qRT-PCR and Western blot analysis (Fig. 4A, B). Based on the findings from CCK-8 and EdU, it has been observed that sh-NUP43 has the ability to impede the growth of HCT116 and SW480 cells. Transwell and scratch studies demonstrated attenuation in the invasion and migration capabilities of HCT116 and SW480 cells when the expression of NUP43 was diminished. The overexpression of NUP43 had a reverse effect (Fig. 4C–F). To summarize, the aforementioned research demonstrates that NUP43 has a role in facilitating the occurrence and progression of CRC both in vitro and in vivo, and additionally contributes to the liver metastasis of CRC.

### NUP43 facilitates the translocation of PD-L1 to the nucleus

**Fig. 5:**
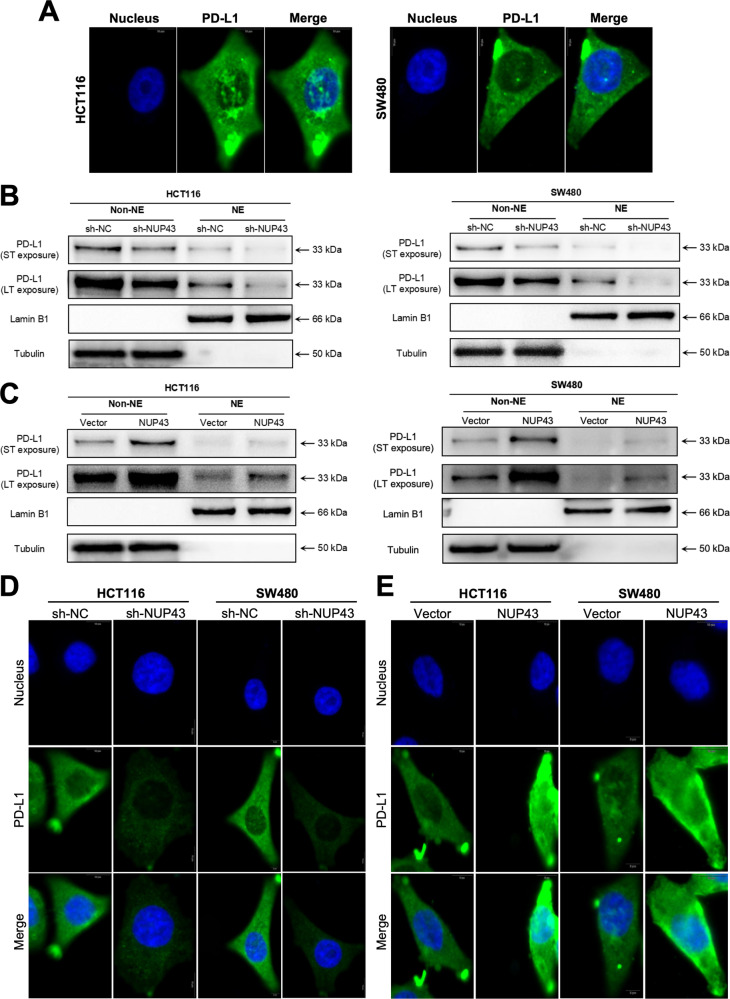
NUP43 facilitates the nuclear translocation of PD-L1. **A** The location of PD-L1 in CRC cells (HCT116 and SW480) was studied using immunofluorescence labeling and confocal imaging. The scale bar used was 5 μm. **B**, **C** Following the separation of cells into several groups (sh-NUP43, sh-NC, Vector, NUP43), a PD-L1 Western blot analysis was conducted.ST exposure refers to a brief duration of exposure, while LT exposure refers to a prolonged duration of exposure. Lamin B1 serves as a marker for nuclear proteins, while tubulin serves as a marker for cytoplasmic proteins. **D**, **E** The cellular localization of PD-L1 was examined in each group using confocal microscopy and immunofluorescence labeling.

Previous research has commonly held the belief that PD-L1 is present in the cytoplasm and cell membrane. However, only a limited number of studies have provided evidence that PD-L1 can also be found in the nucleus of various tissues, such as esophageal cancer, lung cancer, and prostate cancer. During the examination of immunofluorescence data from CRC tissue, we observed the presence of PD-L1 expression in the nucleus. Simultaneously, we have also discovered a potential correlation between this expression and the expression of NUP43. Hence, we proceeded with additional investigations about the subcellular distribution of PD-L1. To investigate the expression and distribution of PD-L1, we conducted immunofluorescence detection on HCT116 and SW480 cells. The findings demonstrated that PD-L1 is present not only in the cell membrane and cytoplasm, but also shows positive expression in the nucleus of CRC cell lines (Fig. 5A). To enhance differentiation, we referred to it as nuclear PD-L1 (nPD-L1). Subsequently, we conducted Western blot studies to isolate cytoplasmic and nuclear proteins from cells in both the NUP43 overexpression group and the control group. The findings once again confirmed the presence of nPD-L1. Additionally, we observed that overexpression of NUP43 resulted in an increase in the expression level of nPD-L1. In contrast, the suppression of NUP43 resulted in a substantial reduction in the expression level of nPD-L1 (Fig. 5B, C). The findings from the immunofluorescence detection experiments provide more evidence in favor of this perspective (Fig. 5D, E and S2A–D). This data validates the presence of nPD-L1 in CRC, and NUP43 can enhance its expression to achieve additional augmentation.

### IPO5 plays a crucial role in the NUP43-mediated nuclear translocation of PD-L1

**Fig. 6:**
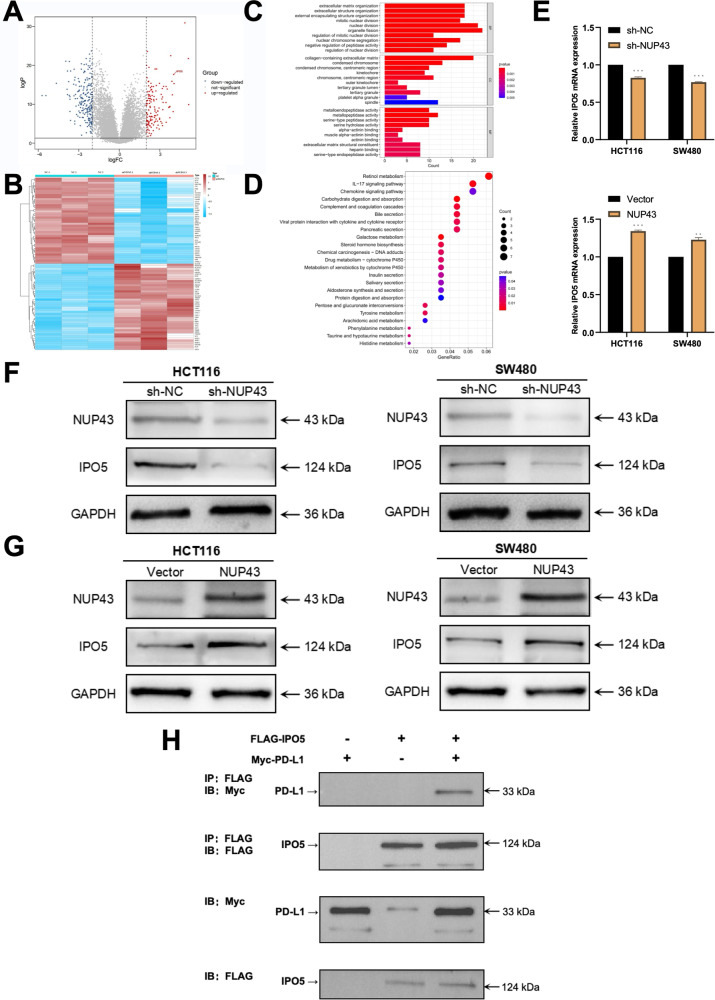
IPO5 plays a role in the NUP43-facilitated movement of PD-L1 into the nucleus. **A** RNA sequencing was employed to assess the differential expression of mRNAs in the sh-NUP43 and sh-NC groups. In the Volcano plot, the color red represents a higher gene expression level in the sh-NC group compared to the sh-NUP43 group, while the color blue represents a lower expression level. An arrow denotes IPO5. **B** Heatmap illustrating the expression levels of mRNAs that are differently expressed between the sh-NUP43 and sh-NC groups. The intensity of the color red directly correlates with the degree of expression. **C** Performing GO analysis on the mRNA in CRC cells treated with sh-NUP43 and sh-NC. **D** Performing KEGG pathway analysis on mRNA in CRC cells treated with sh-NUP43 and sh-NC. **E** The expression of IPO5 mRNA was detected by qRT-PCR in cells from each group, namely sh-NUP43, sh-NC, Vector, and NUP43. **F**, **G** Performing Western blot analysis to detect the presence of NUP43 and IPO5 proteins in each cell group. **H** The HEK293T cells were transfected with the specified plasmids. The interaction between PD-L1 and IPO5 was assessed using Western blotting and co-IP techniques. *P < 0.05; **P < 0.01; ***P < 0.001; ****P < 0.0001.

While the aforementioned findings provide evidence for the presence of nPD-L1 in CRC and the influence of NUP43 on it, the mechanism by which NUP43 triggers the nuclear translocation of PD-L1 remains unidentified. In order to investigate the process by which NUP43 causes the transportation of PD-L1 to the nucleus in CRC cells and assess its influence on subsequent gene expression, we conducted RNA sequencing. In comparison to the sh-NC and sh-NUP43 groups, the sh-NUP43 group exhibited a substantial upregulation of 311 mRNAs and a significant downregulation of 398 mRNAs (Fig. 6A, B). The GO analysis results indicate that the differential genes mostly control cell proliferation activity by influencing processes such as cell mitosis, nuclear division, and nuclear chromosome segregation (Fig. 6C). By conducting KEGG analysis, we discovered that the differentially expressed genes were primarily enriched in pathways that had an impact on cellular energy metabolism (Fig. 6D). During our gene screening process, we observed notable disparities between the two groups. Specifically, we discovered that IPO5 may have a connection to the NUP43-mediated nuclear translocation of PD-L1. Research has indicated that IPO5, belonging to the nuclear transport protein family, can facilitate the onset and progression of several disorders, such as cancers, by regulating the transportation of substances within and outside the nucleus [34–36]. Our current objective was to investigate whether IPO5 likewise possesses this effect regarding PD-L1. Initially, we determined the presence of identical trends in IPO5 and NUP43 at the mRNA and protein levels by qRT-PCR and western blotting assays (Fig. 6E–G). Subsequently, we conducted a co-immunoprecipitation assay in HEK 293 T cells, where we co-expressed Myc-tagged PD-L1 and Flag-tagged IPO5. This allowed us to validate the natural interaction between the PD-L1-IPO5 complex (Fig. 6H).

**Fig. 7:**
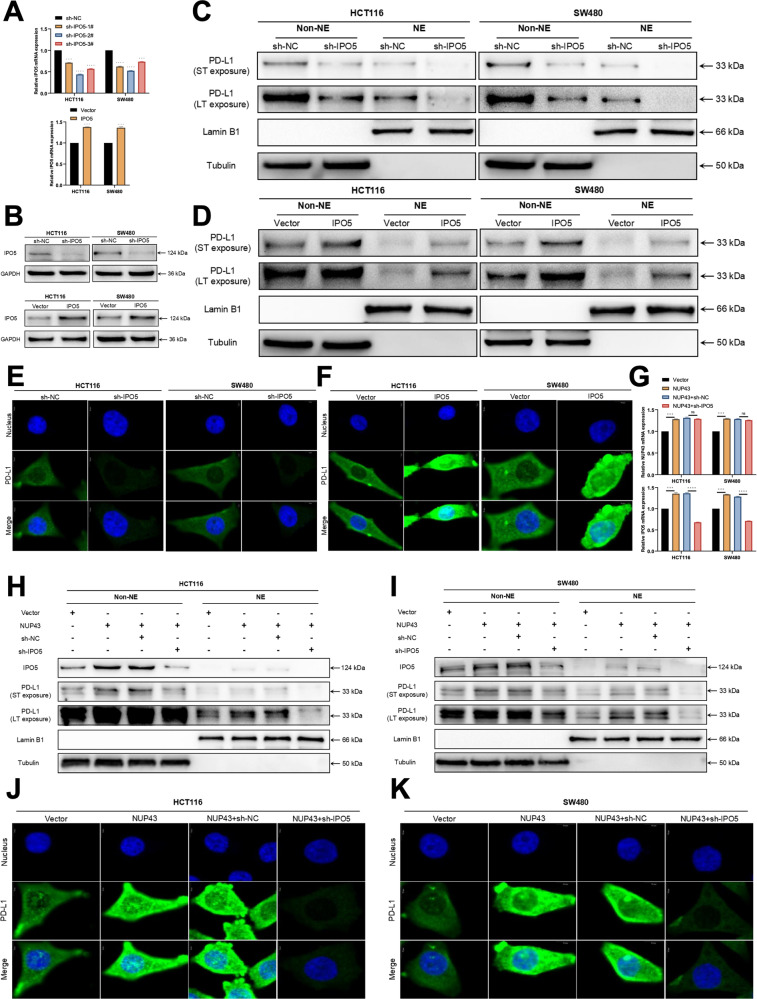
IPO5 is an indispensable key molecule during the NUP43-mediated nuclear translocation of PD-L1. **A**, **B** Three shRNAs (sh-IPO5-1#, sh-IPO5-2#, and sh-IPO5-3#) were designed to silence CRC cells (HCT116 and SW480) and verified by qRT-PCR and Western blotting. We used sh-IPO5-2# to downregulate IPO5. IPO5 overexpression was verified in CRC cells (HCT116 and SW480) by qRT-PCR and Western blotting. **C**, **D** Following the division of cells into several groups (sh-IPO5, sh-NC, Vector, IPO5) based on nucleocytoplasmic distribution, PD-L1 Western blot analysis was conducted. **E**, **F** Confocal microscopy was used to assess the immunofluorescence staining of PD-L1 in cells from each group. **G** qRT-PCR was conducted to identify the presence of NUP43 and IPO5 mRNA in cells from each experimental group (Vector, NUP43, NUP43+sh-NC, and NUP43+sh-IPO5). **H**, **I** Perform Western blot analysis to examine the expression levels of IPO5 and PD-L1 in each cell group. **J**, **K** Confocal microscopy was used to assess the immunofluorescence staining of PD-L1 in cells from each group. *P < 0.05; **P < 0.01; ***P < 0.001; ****P < 0.0001.

To further elucidate the function of IPO5 in the nuclear translocation of PD-L1, we devised three distinct shRNAs to suppress the expression of IPO5. qRT-PCR and Western blot tests demonstrated a decrease in cell IPO5 expression following shRNA transfection (Fig. 7A, B). Subsequently, we isolated the nucleoplasm from the cells in both the IPO5 knockdown group and the control group, and obtained the respective proteins for conducting Western blotting studies. The results were in line with the predictions. When the level of IPO5 expression fell, the level of PD-L1 expression in the nucleus also decreased proportionally. In the group where IPO5 was overexpressed, there was a considerable rise in the expression of PD-L1 in the nucleus as the level of IPO5 increased (Fig. 7C, D). The findings of cell immunofluorescence detection also exhibited a consistent pattern (Figs. 7E and S2E, F, Figs. 7F and S2G, H). To further validate the association between NUP43, IPO5, and PD-L1 nuclear translocation, we employed plasmid and lentiviral transfection techniques to enhance the expression of NUP43 in CRC cells. Simultaneous treatment was administered to induce NUP43 overexpression and knockdown of IPO5. The cells were categorized into four groups based on the treatment approach: Vector group, Vector+NUP43 overexpression group, Vector+NUP43 overexpression+sh-NC group, and Vector+NUP43 overexpression+IPO5 knockdown group (Fig. 7G). The four groups underwent nucleocytoplasmic separation, followed by Western blotting investigations and cellular immunofluorescence detection experiments. The experimental findings demonstrated that the nuclear aggregation of PD-L1 induced by the overexpression of NUP43 can be counteracted by suppressing the expression of IPO5 (Fig. 7H–K and S2I, J). Our experimental findings conclusively demonstrate that the nuclear translocation of PD-L1, facilitated by NUP43, is accomplished through the upregulation of the PD-L1 binding protein IPO5.

### High nPD-L1 expression can enhance the TM4SF1 level

**Fig. 8:**
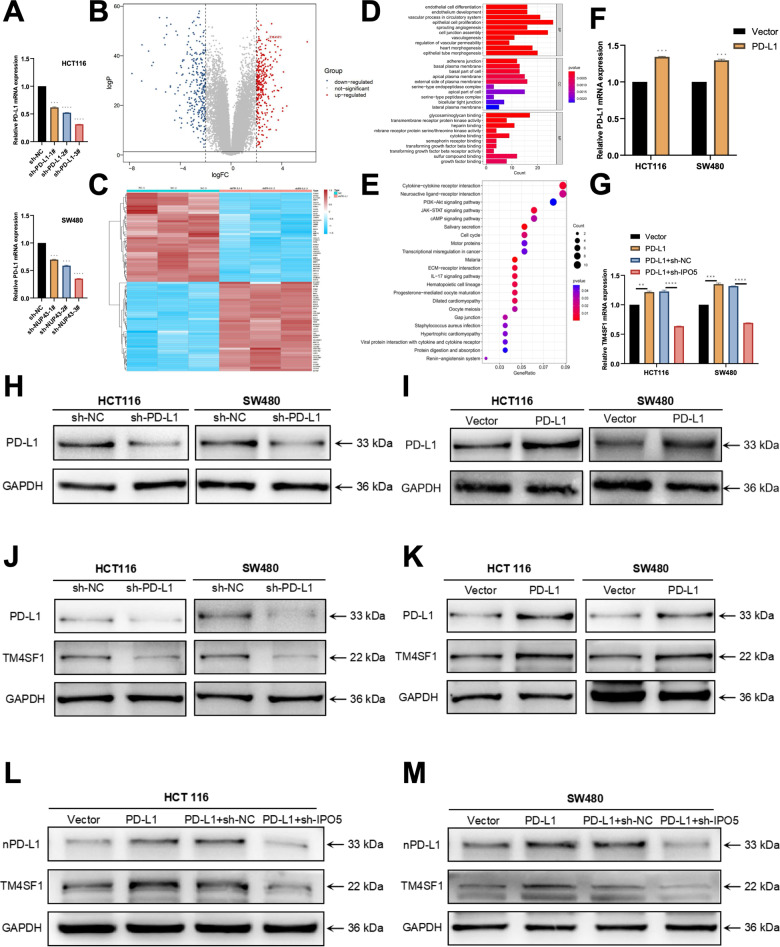
Once PD-L1 penetrates the nucleus, it stimulates the activation of the cancer-promoting factor TM4SF1. **A**, **H** Three shRNAs (sh-PD-L1-1#, sh-PD-L1-2#, and sh-PD-L1-3#) were designed to silence CRC cells (HCT116 and SW480) and analyzed by qRT-PCR and Western blotting for verification. We used sh-PD-L1-3# to downregulate PD-L1. **B** RNA sequencing was employed to assess the differential expression of mRNAs in the sh-PD-L1 and sh-NC groups. In the Volcano plot, the color red signifies that the gene expression level of the sh-NC group is greater than that of the sh-PD-L1 group, while the color blue suggests a lower expression level. TM4SF1 is denoted by an arrow. **C** Heatmap displaying the mRNAs that are expressed differently between the sh-PD-L1 and sh-NC groups. Greater intensity of the color red corresponds to a higher degree of expression. **D** Performing GO analysis on mRNA in CRC cells treated with sh-PD-L1 and sh-NC. **E** Performing KEGG pathway analysis on mRNA in CRC cells treated with sh-PD-L1 and sh-NC. **F**, **I** PD-L1 overexpression in CRC cells (HCT116 and SW480) was confirmed using qRT-PCR and Western blotting techniques. **G** The expression of TM4SF1 mRNA in cells from each group (Vector, PD-L1, PD-L1+sh-NC, and PD-L1+sh-IPO5) was detected by qRT-PCR. **J**, **K** Western blot analysis was performed to examine the expression of TM4SF1 and PD-L1 in each experimental group, including sh-NC, sh-PD-L1, Vector, and PD-L1. **L**, **M** Western blot analysis was performed on cells from each group, namely Vector, PD-L1, PD-L1+sh-NC, and PD-L1+sh-IPO5, to detect TM4SF1 protein expression. Following the separation of cells into nuclear and cytoplasmic fractions, the nuclear protein fraction was subjected to PD-L1 Western blot analysis. *P < 0.05; **P < 0.01; ***P < 0.001; ****P < 0.0001.

Subsequently, to validate the role of PD-L1 within the nucleus, we devised three distinct shRNAs to suppress PD-L1 expression. The results of qRT-PCR and Western blot assays demonstrated that transfection of sh-PD-L1 in CRC cells led to a decrease in PD-L1 expression (Fig. 8A, H). Next, we conducted RNA sequencing on cells in the sh-NC group and sh-PD-L1 group and scrutinized the related data. In the sh-PD-L1 group, we observed a substantial upregulation of 281 mRNAs and a significant downregulation of 355 mRNAs (Fig. 8B, C). The results of the GO analysis showed that the differentially expressed genes were primarily enriched in biological processes such as blood vessel development, cell adhesion, and cell differentiation, which played a crucial role in regulating tumor growth and metastasis (Fig. 8D). In addition, we identified TM4SF1 throughout the process of screening genes that exhibited clear distinctions. TM4SF1 has been extensively cited in prior research as a facilitator of tumor growth. According to reports, TM4SF1 has the ability to enhance the metastasis of esophageal squamous cell carcinoma by forming a connection with integrin α6 [37]. Furthermore, TM4SF1 has been well-documented for its ability to initiate carcinogenesis in hepatocellular carcinoma, ovarian cancer, pancreatic cancer, and other malignancies [38–40]. Our research findings indicate that the expression level of TM4SF1 in CRC has a similar pattern to that of PD-L1(Fig. 8F, I–K).

To enhance our understanding of the role of PD-L1 both within and outside the nucleus, we designed the subsequent studies. Prior reports indicate that elevated expression of PD-L1 leads to a corresponding accumulation of PD-L1 in the nucleus to a certain degree [41]. Therefore, we used lentivirus and plasmid to transfect CRC cell lines and divided the cells into the control group (Vector), PD-L1 overexpression group (PD-L1), PD-L1 overexpression+sh-NC group (PD-L1+sh-NC) and PD-L1 overexpression + IPO5 knockdown group (PD-L1 + sh-IPO5) (Fig. 8G). The findings indicated that as the level of PD-L1 expression in CRC cells increased, PD-L1 accumulated within the nucleus, and the expression of TM4SF1 was also markedly elevated. Following the overexpression of PD-L1 and the knockdown of IPO5, which resulted in the loss of the PD-L1 binding protein, there was an increase in the overall expression of PD-L1 in the cells. However, the expression of nPD-L1 decreased, and there was also a reduction in the expression of TM4SF1 (Fig. 8L, M). The aforementioned findings indicate that the nuclear entrance of PD-L1 is responsible for stimulating the expression of TM4SF1 in CRC.

### TM4SF1 can initiate the JAK/STAT3 signaling pathway to increase the expression of total cell PD-L1 and promote the development of CRC

**Fig. 9:**
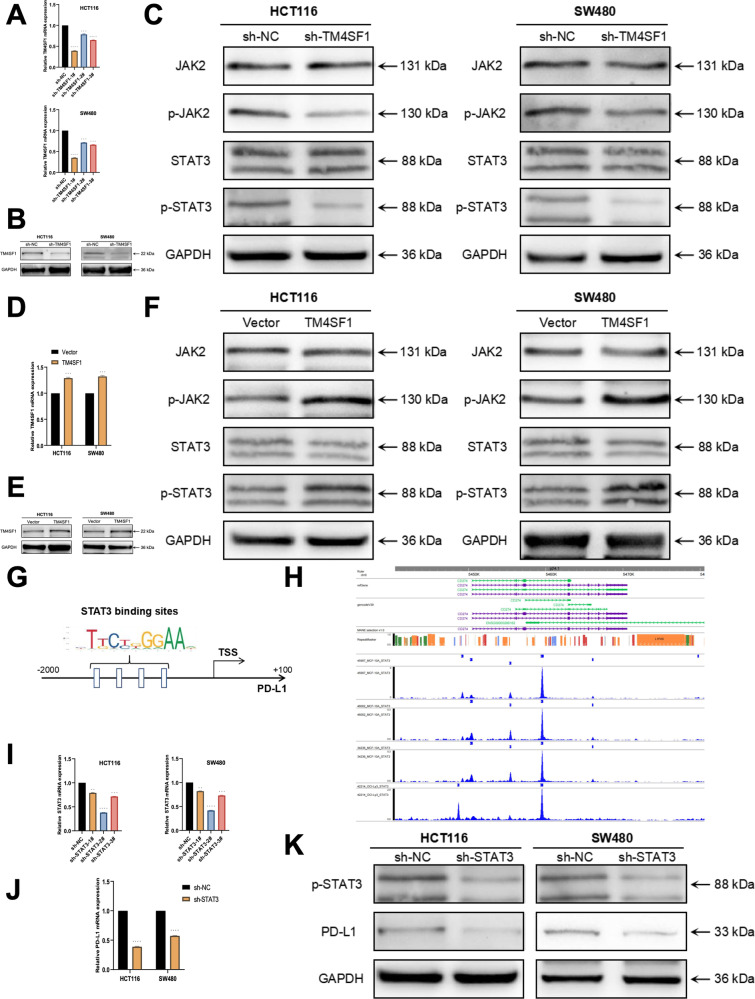
TM4SF1 enhances the process of PD-L1 gene transcription by stimulating the JAK/STAT3 signaling pathway. **A**, **B**, **D**, **E** Three shRNAs (sh-TM4SF1-1#, sh-TM4SF1-2#, and sh-TM4SF1-3#) were designed to silence CRC cells (HCT116 and SW480) and were analyzed by qRT-PCR and Western blotting. We used sh-TM4SF1-1# to downregulate TM4SF1. TM4SF1 overexpression was verified in CRC cells (HCT116 and SW480) by qRT-PCR and Western blotting. **C**, **F** The Western blot analysis reveals the levels of JAK2, phospho-JAK2, STAT3, and phospho-STAT3 expression in CRC cells with either reduced or increased TM4SF1 expression. **G** According to the JASPAR database, it was anticipated that the promoter of the PD-L1 gene contains the binding site for STAT3. **H** The ChIP data for GSM935457, GSM935399, GSM935551, and GSM1227206 revealed a distinct peak of STAT3 located upstream of PD-L1. **I** Three shRNAs (sh-STAT3-1#, sh-STAT3-2#, and sh-STAT3-3#) were designed to silence CRC cells (HCT116 and SW480) and verified by qRT-PCR. We used sh-STAT3-2# to downregulate STAT3. **J** The expression of PD-L1 mRNA in cells of each group (sh-NC, sh-STAT3) was detected by qRT-PCR. **K** Western blot analysis was conducted to assess the efficacy of sh-STAT3 silencing in each group, and to analyze the expression of PD-L1 in cells from each group. *P < 0.05; **P < 0.01; ***P < 0.001; ****P < 0.0001.

Upon conducting KEGG analysis on RNA-seq data, we discovered a high correlation between the differentially expressed genes and the JAK/STAT3 signaling pathway (Fig. 8E). Prior research has demonstrated that TM4SF1 has the ability to initiate the JAK2/STAT3 signaling pathway in breast cancer, hence facilitating the spread of breast cancer to several target organs [42]. Furthermore, based on the prior research findings of this study, we noted that the reduction of PD-L1 accumulation in the nucleus, either through the NUP43 or IPO5 pathways, leads to a substantial decrease in overall PD-L1 expression in the cells. Hence, we conducted additional investigations to determine if TM4SF1 has the ability to initiate the JAK/STAT3 signaling pathway and consequently impact the expression of PD-L1. For this purpose, three shRNAs were designed to specifically suppress the expression of TM4SF1 in HCT116 and SW480 cells. The effectiveness of sh-TM4SF1-1# in silencing TM4SF1 was assessed by qRT-PCR and western blotting (Fig. 9A, B). Our investigation revealed that the decreased expression of TM4SF1 resulted in the inhibition of the phosphorylation of JAK2 and STAT3 (Fig. 9C). Nevertheless, the overexpression of TM4SF1 regulated the activation of the JAK/STAT3 signaling pathway (Fig. 9D–F). Subsequently, we examined the correlation between the JAK/STAT3 signaling pathway and PD-L1. The JASPAR database indicated that the promoter region of the PD-L1 gene is expected to encompass the binding site for STAT3 (Fig. 9G). The ChIP data for GSM935457, GSM935399, GSM935551, and GSM1227206 revealed a prominent STAT3 peak upstream of PD-L1, providing evidence of a distinct binding site between STAT3 and PD-L1 (Fig. 9H).

Simultaneously, based on the findings from qRT-PCR and western blotting, the downregulation of STAT3 leads to a decrease in both the mRNA and protein levels of PD-L1 (Fig. 9I–K). The aforementioned findings clearly indicate that TM4SF1 enhances the production of PD-L1 via activating the JAK/STAT3 signaling pathway. Ultimately, in order to demonstrate that PD-L1 has the ability to control the growth of CRC without relying on the immunosuppressive mechanism, we employed CCK-8 and EdU techniques to assess the proliferation efficiency of CRC cells at varying levels of PD-L1 expression. The findings demonstrated that suppressing PD-L1 effectively impeded cellular proliferation. In contrast, the cellular growth efficiency was dramatically increased in cells that had an overexpression of PD-L1 (Fig. S4A, B). The Transwell invasion assay and scratch assay demonstrated that the downregulation of PD-L1 considerably impeded the invasive capabilities of HCT116 and SW480 cells. Conversely, the PD-L1 overexpression model had the opposite impact (Fig. S4C, D). Hence, according to the aforementioned experimental findings, we have discovered that the increase in TM4SF1 expression induced by the entrance of PD-L1 into the nucleus triggers the activation of the JAK/STAT3 signaling pathway, leading to an enhancement in overall cell PD-L1 expression. Consequently, this process ultimately contributes to the facilitation of CRC initiation and progression.

## Discussion

Despite significant progress in clinical therapies, the survival rate of CRC patients has substantially increased. However, the primary cause of the high mortality rate in CRC patients is currently attributed to tumor metastasis, and the underlying mechanism of this biological process is still unknown [43, 44]. In order to achieve this objective, we designed a CRISPR/Cas9 screening that examines the entire genome. This technique allows us to thoroughly investigate genes that could potentially facilitate the growth and advancement of CRC, and their involvement in the metastasis of cancer cells to other parts of the body. Through our research, we have identified nucleoporin NUP43 as a significant catalyst in the formation of metastasis in CRC. NUP43 is widely recognized for its significant involvement in various biological processes. It serves as a stable constituent of the Nup107-160 complex and is positioned at the centromere during mitosis to oversee the advancement of mitosis and the separation of chromosomes. The Nup107-160 complex potentially facilitates the nuclear translocation of phosphorylated ERK to control the malignant transformation of senescent cells [45, 46]. NUP43 overexpression contributes to the advancement of multiple malignant cancers, including gastric cancer, breast cancer, and ovarian cancer [47–49]. Nevertheless, the role of NUP43 in CRC has not been reported. Our investigation revealed that NUP43 shows great potential as a prognostic indicator for patients with CRC. Significantly, our findings indicate that the increased expression of NUP43 in patients with CRC plays a role in the buildup of PD-L1 within the nucleus of tumor cells.

There has been a consistent increase in the number of confirmed instances of nuclear PD-L1 in recent years. However, there is still a limited amount of research available on the specific process by which PD-L1 gets transported to the nucleus [26, 27]. Through our investigation of the mechanism by which NUP43 facilitates the build-up of PD-L1 in the nucleus in CRC, we identified the chaperone protein IPO5 that is responsible for PD-L1. Nucleocytoplasmic transport is a crucial cellular process that plays a vital role in cell metabolism and viability. Any impairment in this process can result in various illnesses, including cancer [50–53]. Importin and exportin 5 are important regulating molecules in this process [54]. IPO5 is a nuclear transport protein belonging to the importin β family. Recent reports indicate that elevated levels of IPO5 can facilitate epithelial-mesenchymal transition (EMT) and enhance the progression of esophageal cancer via the RAS-ERK signaling pathway [35]. The Kaplan–Meier survival study revealed a significant association between high IPO5 expression and shorter survival in CRC patients, compared to those with low IPO5 expression (Fig. S1A). Furthermore, our research also discovered that suppressing IPO5 can counteract the nuclear buildup of PD-L1 in CRC resulting from the increased expression of NUP43. This indicates that IPO5 has a significant function in the NUP43-mediated movement of PD-L1 into the nucleus.

The involvement of PD-L1 in facilitating tumor cell escape from immune detection has been extensively debated. The immune system of the host demonstrates anti-tumor behavior by stimulating immunological responses. The interaction between PD-L1 and PD-1 on T cells results in the removal of phosphate groups from T cell receptors, which hinders the anti-tumor effect of T cells by decreasing their proliferation and activity [55]. PD-L1 or PD-1 monoclonal antibodies, known as immune checkpoint inhibitors, have been employed in the treatment of various types of malignancies, including melanoma, non-small cell lung cancer, gastric cancer, and breast cancer [56–59]. Despite the notable clinical advantages demonstrated by PD-1/PD-L1 blocking therapy in different types of cancer, the rate of positive response among patients remains below 40%, and the underlying mechanism remains uncertain. Therapies that block the PD-1 or PD-L1 pathway in CRC have a narrower range of effects. The US Food and Medicine Administration (FDA) authorized pembrolizumab as a second-line treatment for patients with microsatellite instability-high (MSI-H) mCRC in 2017, making it the first medicine of its kind to receive approval. Nevertheless, the population of dMMR/MSH-H patients is limited, comprising around 15% of CRC patients and 4% of mCRC patients. Additionally, a subset of these patients rapidly progress to the immunological resistance phase [60]. Furthermore, it is important to highlight that recent research has discovered that PD-L1 relocates to the nucleus and has an impact on tumors that is unrelated to the immune system. These effects include stimulating cell growth, spreading to other parts of the body, influencing metabolism, and developing resistance to immunotherapy [20–23]. Patients with elevated PD-L1 expression in CRC also exhibit a worse prognosis (Fig. S1B). The influence of nPD-L1 on CRC and its underlying mechanism remains an unresolved issue. This work discovered, for the first time, that PD-L1 is transported into the nucleus and subsequently controls the expression of TM4SF1. TM4SF1 has been consistently identified as an oncogenic factor, facilitating the initiation and progression of several malignancies. The findings from the Kaplan–Meier survival analysis indicate that CRC patients who exhibit elevated levels of TM4SF1 expression experience a more unfavorable outcome (Fig. S1C). In addition, our work revealed that the increase in TM4SF1 expression can stimulate the JAK/STAT3 signaling pathway, leading to an elevation in the overall expression of PD-L1 in cells. This eventually contributes to the initiation and progression of CRC.

**Fig. 10:**
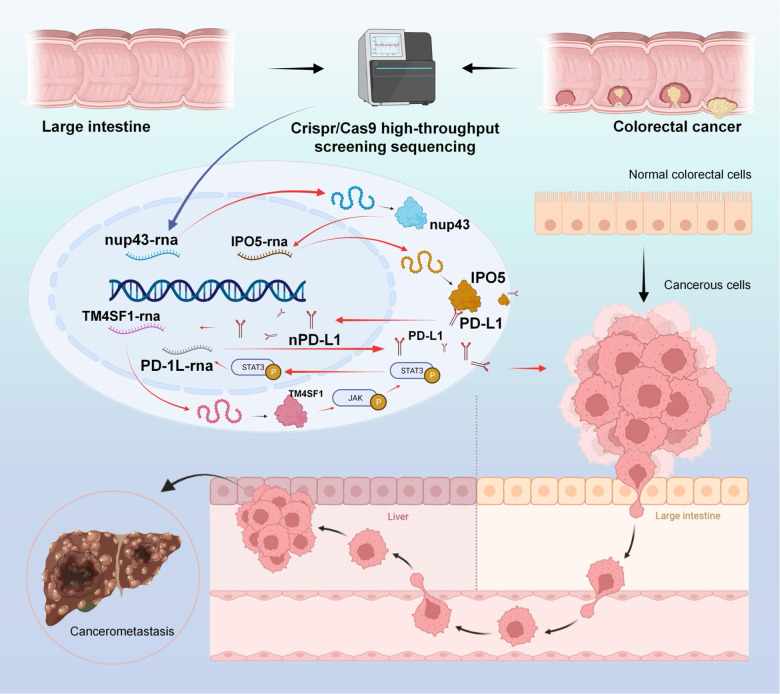
The schematic picture demonstrates that the oncogenic factor NUP43 enhances the nuclear transport of PD-L1 by controlling the expression of the PD-L1 chaperone protein IPO5. Nuclear PD-L1 stimulates the expression of the tumor-promoting factor TM4SF1 and activates the JAK/STAT3 signaling pathway. This ultimately leads to increased transcription of PD-L1, establishing a cyclic enhancement system involving PD-L1, nPD-L1, and PD-L1. Consequently, this system promotes the occurrence and progression of CRC and liver metastasis.

Our research demonstrates that NUP43 facilitates the movement of PD-L1 into the cell nucleus via IPO5, thereby increasing the expression of the oncogenic factor TM4SF1. This process activates the JAK/STAT3 signaling pathway, augmenting the overall expression of PD-L1 in colorectal cancer (CRC) cells. Notably, our study unveils a feedback loop involving PD-L1, nPD-L1, and PD-L1, which significantly contributes to CRC progression and metastasis formation (Fig. 10). These findings hold promise for the identification of prognostic biomarkers in CRC, and provide fresh insights into the molecular mechanisms underlying tumor metastasis, and furnish valuable preclinical data for the development of systemic treatments targeting this formidable disease.

## Materials and methods

### CRISPR/Cas9 sequencing and data analysis

The construction of the CRISPR Screen II sequencing library was carried out in the following manner. The library was constructed using a two-step PCR process, with different reaction numbers chosen based on the sample categories. Primer sequences are designed to flank the sgRNA sequence in the specified area, and the reaction system is assembled using high-fidelity enzymes. PCR was initially employed to get the desired fragment, with the cycle count typically limited to 18–22 cycles. For the second phase, PCR was conducted by introducing primers (Index) and adapters (Adapter) to acquire the desired fragments, with the cycle count typically limited to 8–12 cycles. Once the two-step PCR process is finished, the adhesive is cleaved and subjected to cycling in order to obtain the desired band. Following the purification of the library, the concentration was measured using qubit 3.0 (Invitrogen, Carlsbad, CA, USA), and the fragment size of the library was detected using 2200 (Agilent Technologies, Palo Alto, USA). An appointment was scheduled for Nano QC at Illumina in San Diego, USA. The DNA libraries, each labeled with distinct index markers, were combined and subjected to 2 × 150 bp dual-end sequencing (PE) following the manufacturer’s instructions for the Illumina instrument (Illumina, San Diego, USA). The HiSeq Control Software (HCS) + OLB+Gapeline-1.6 (Illumina) was utilized to analyze images and detect bases in the HiSeq instrument. Ultimately, the unprocessed sequencing data was examined by bioinformatics experts at GENEWIZ.

The sgRNAs (19–21 bp) were obtained by extracting target sequences using a custom script that considered the 5 bp bases upstream and downstream. The extracted sgRNAs were then subjected to quality control to get clean reads. The clean readings were compared to the sgRNA library using MAGeCK software, and the resulting counts were recorded. The results of differential gene analysis and GSEA enrichment analysis were compared using MAGeCK software.

### Patient and tissue specimen collection

Prior to commencing the study, we adhered to the guidelines outlined in the Declaration of Helsinki by providing all participants with a clear explanation of the study’s process and objectives. Furthermore, we received written consent from all patients after ensuring they were fully informed about the study. The Medical Ethics Committee of Nanjing Medical University has granted prior approval for the complete procedure of human specimen collecting. For immunofluorescence detection, we obtained cancerous and nearby normal intestinal samples from 5 individuals diagnosed with primary colorectal cancer.

### RNA extraction and qRT-PCR

To assess the effectiveness of the lentivirus we created to target NUP43, PD-L1, TM4SF1, IPO5, and STAT3 in human CRC cell lines HCT116 and SW480, as well as mouse CRC cell lines MC38 and CT26, we performed qRT-PCR. Total RNA was extracted from the aforementioned cells using TRIzol reagent (Invitrogen, USA) according to the manufacturer’s instructions. The RNA was converted into complementary DNA (cDNA) using a reverse transcription kit manufactured by Takara in Japan. Tables S1, S2 provides a comprehensive list of all primer sequences. The mRNA expression levels were standardized using an internal control (GAPDH).

### RNA sequencing and pathway analysis

RNA was extracted from 5 × 10^6^ CRC cells that were treated with sh-NUP43 or sh-NC and sh-PD-L1 or sh-NC. The development of the library and the sequencing process were carried out by Shenzhen BGI (BGI, China). The sequences of lncRNA, mRNA, and circRNA were determined. The high-throughput sequencing process involves converting the raw picture data files into sequencing reads using base calling analysis. The Kyoto Encyclopedia of Genes and Genomes (KEGG) pathway annotation and enrichment were performed utilizing the DAVID platform (https://david.ncifcrf.gov/). We deemed routes with a Q value ≤0.05 to be considerably enriched. The procedure of functional annotation was carried out by adhering to the Gene Ontology database, which includes classifications such as biological process, cellular component, and molecular function. We used a Fisher exact test to identify important categories. The Gene Ontology (GO) words with a computed Q value ≤0.05 demonstrated statistical significance.

### Cell and cell culture

We acquired human CRC cell lines and mice CRC cell lines, such as HCT116, SW480, MC38, and CT26, from the Cell Bank of Type Culture Collection at the Chinese Academy of Sciences in China. The cells collected were grown in DMEM medium (BI, USA) with the addition of 10% fetal bovine serum (FBS; Gibco, USA). A temperature-controlled incubator set at a constant 37 °C and with a CO_2_ concentration of 5% was utilized for cell culture.

### Cellular fractionation, western blot, and co-immunoprecipitation assay

The cellular fractions were obtained by utilizing the nuclear protein extraction kit (R0050, Solarbio Life Science). Typically, cells were initially broken down using cytoplasmic protein extraction buffer, and then nuclear extraction buffer was used to isolate the nucleus fraction. The analysis employed antibodies against PD-L1, Tubulin, Lamin B1, NUP43, IPO5, GAPDH, TM4SF1, JAK2, p-JAK2, STAT3, and p-STAT3 from Abcam (UK). Additionally, Flag antibodies from Sigma (St. Louis, MO, USA) and secondary antibodies against mouse or rabbit from Cell Signaling Technology were used. In immunoprecipitation experiments, protein lysates were subjected to incubation with anti-PD-L1, anti-IPO5, anti-Flag, anti-Myc, or the normal IgG antibody at a temperature of 4 °C for an extended period of time overnight, with continuous rotation. On the second day, the mixture was also cultured with protein A/G beads or M2 anti-Flag resin at room temperature for a duration of 2–3 h. After undergoing three rounds of washing with lysis buffer, the beads were subjected to boiling and thereafter proceeded with a western blot assay.

### Cell proliferation assay

The HCT116 and SW480 cells were partitioned into an experimental group and a control group, and subsequently placed in separate 96-well plates. A total of 1000 cells were distributed evenly in each well using 100 μL of media, and subsequently exposed to 10 μL of CCK-8 solution (RiboBio, China). Cell absorbance at 450 nm was measured using a microplate reading element at 0, 24, 48, and 72 h of culture. The measurement approach adhered to the manufacturer’s specifications (Synergy, USA).

We conducted Cell-Light 5-ethynyl-2’-deoxyuridine (EdU) studies to assess the ability of cells to proliferate using the EdU DNA Cell Proliferation Kit (RiboBio, China). 50,000 cells were seeded in each well of a 24-well plate. Following regular culture, the cells were exposed to a 50 mmol/L EdU solution for a duration of 2 h. Subsequently, they were fixed using a 4% paraformaldehyde solution. According to the instructions provided by the manufacturer, we applied Apollo Dye Solution and DAPI to the cell lines. Subsequently, we used an Olympus FSX100 microscope (Olympus, Japan) to capture and count the cells.

### Transwell migration and invasion assays

According to the instructions provided by the manufacturer, we introduced 200 μl of serum-free DMEM media into the upper chamber. Subsequently, we separately placed 20,000 HCT116 cells and SW480 cells in the chamber, dividing them into experimental and control groups. In order to evaluate the ability of cells to invade, we prepared Transwell chambers (manufactured by Corning, USA) by coating them with a mixture of Matrigel (produced by BD Biosciences, USA). The Matrigel mixture was excluded for the evaluation of cell migratory capacity. A total of 700 microliters of DMEM media, which contained 10% FBS, was added to the lower chamber in order to bind the chemotactic agent of the CRC cells. Following a 24-h incubation in a standard incubator, the upper chamber was taken out. The serum-free media was then removed and the cells were treated with 4% paraformaldehyde for 10 min to fix them. Subsequently, the cells were stained with crystal violet (Kaigen, China) for 15 min and washed with PBS. The cell lines were visually captured under a microscope and five regions were selected for quantification.

### Wound healing assay

HCT116 and SW480 cells were placed on six-well culture plates in order to facilitate the post-transfection process. The fused cell monolayer was manually treated using a conventional 20 μl pipette tip to eliminate linear wounds. Following the rinsing of the suspended cells with PBS, introduce the entire medium and place them in a 37 °C incubator for incubation. Images were captured using an inverted microscope at 0, 24, and 48 h, and the width of the scratch was quantified. The experiments were conducted in duplicate for each group.

### Immunofluorescence and immunohistochemistry assays

In order to conduct immunofluorescence tests, we sliced the paraffin-embedded material, which consisted of primary colorectal cancer and matching intestine tissue taken from individuals with colorectal cancer, into sections that were 4 mm in thickness. The cells were treated with a 4% formaldehyde solution and kept at room temperature for 20 min to fix them. Treat with PBS solution containing 0.05% Triton X-100 (Sigma-Aldrich, USA) for a duration of 5 min to increase permeability. The samples were immersed in a solution of phosphate-buffered saline (PBS) containing 2% bovine serum albumin (BSA) and kept sealed for a duration of 1 h. The antibody NUP43 and PD-L1 (Abcam, UK) were incubated at a temperature of 4 °C overnight. Afterward, Alexa fluorine-HRP conjugated secondary antibodies (Abcam, UK) were added and incubated for one hour at room temperature. DAPI, obtained from Sigma-Aldrich in the USA, was utilized for reverse staining the nuclei, which were subsequently photographed. In our immunohistochemical tests, we initially subjected the samples (which included samples of CRC liver metastases from mice in various treatment groups) to overnight incubation at a temperature of 4 °C. Subsequently, we introduced 3’-diaminobenzidine, as well as specific antibodies for NUP43, TUNEL, and KI-67. The immunohistochemistry and immunofluorescence images were analyzed using ImageJ software to determine the fraction of positive regions. These data were then used for further statistical analysis.

### Animal models

Our animal research were authorized by the Animal Care Committee of Nanjing Medical University. All operations adhered to the ethical criteria set by the animal experimental facilities. The mice utilized in our investigations were kept in a controlled environment with specific pathogen-free (SPF) conditions at the Experimental Animal Center of Nanjing Medical University. The mice were euthanized by cervical dislocation.

In order to create a mouse model with tumors under the skin, we injected either MC38 cells or CT26 cells (1 × 10^6^) treated with NUP43 shRNA (sh-NUP43) into the right groin of C57BL/6 mice (*n* = 5 per group). The control group consisted of cells that were treated with control shRNA (sh-NC). The dimensions of the tumor were recorded at 4-day intervals, and the volume of the tumor was determined using the formula: volume (mm^3^) = width^2^ × length/2. The mice were euthanized on the 28th day.

In order to create a mouse model of liver metastasis in CRC, we administered a total of 2 × 10^6^ MC38 cells and CT26 cells (including sh-NC and sh-NUP43) into the spleen of male C57BL/6 mice. The precise protocol involves initially administering an intraperitoneal injection of 0.5% sodium pentobarbital (50 mg/kg) to anesthetize the mice, followed by weighing them. Once the mice were rendered unconscious, they were securely fastened to the operating table using tape. Following the disinfection of the skin, a vertical cut (about 0.5 cm) was performed along the left axillary line of the mouse, just below the back edge of the rib cage. A willow spleen was discovered and extracted from the abdominal cavity. Subsequently, a suspension with a high concentration of either MC38 or CT26 cells (2 × 10^6^ cells) was promptly injected into the lower region of the spleen, and the site of injection was cleansed using an alcohol swab. The abdominal muscles and skin were sutured at intervals following the absence of any bleeding. Following the surgical procedure, the mice were exposed to specific pathogen-free (SPF) settings within an animal laboratory. Grouped as follows: sh-NC (*n* = 5) and sh-NUP43 (*n* = 5) in each group. On the 20th day, the mice were euthanized, and their liver protein expression was analyzed by immunohistochemistry analysis.

### GEO analysis, TCGA data analysis, and site prediction

The tcgportal (http://tumorsurvival.org/index.html) was utilized to assess the correlation between NUP43 expression and the prognosis of patients with CRC. We utilized the hTFtarget database (http://bioinfo.life.hust.edu.cn/hTFtarget#!/) to examine the transcriptional binding sites of PD-L1 and STAT3. To identify the STAT3 binding sites in the PD-L1 gene promoter, we employed the JASPAR database (http://jaspar.genereg.net/). We obtained ChIP data from GSM935457, GSM935399, GSM935551, and GSM1227206 in order to identify binding sites of human PD-L1 and STAT3. The ChIP-Atlas “Peak Browser” feature was utilized to acquire peak-call data for transcription factors and display protein binding at specific genomic locations using the IGV Genome Browser.

### Statistical analysis

The software GraphPad Prism 9.0 was utilized for statistical analysis. The data were presented in the format of mean ± SD (Standard Deviation). The two-tailed independent samples *t*-test was employed to compare two distinct samples. ANOVA was employed to ascertain the variation either within or among groups. The Kaplan–Meier Plotter was utilized for prognostic analysis. A *P* < 0.05 reported statistical significance.

### Supplementary information

Supplemental Figures

Supplementary figure legends and Supplementary Tables

wb&CO-IP RAW DATA

## Data Availability

The supplementary data files and this published article contain the data that were analyzed for this investigation. Additional supporting data were available from the corresponding authors upon reasonable request.
