# Supplementary material for: NUP43 promotes PD-L1/nPD-L1/PD-L1 feedback loop via TM4SF1/JAK/STAT3 pathway in colorectal cancer progression and metastatsis
Source: Cell Death Discov. 2024 May 18;10:241. doi: 10.1038/s41420-024-02025-z (PMC11102480; doi:10.1038/s41420-024-02025-z)
Supplement: Supplementary file 1 — Supplemental Figures [file 41420_2024_2025_MOESM1_ESM.pptx]

## Slide 1
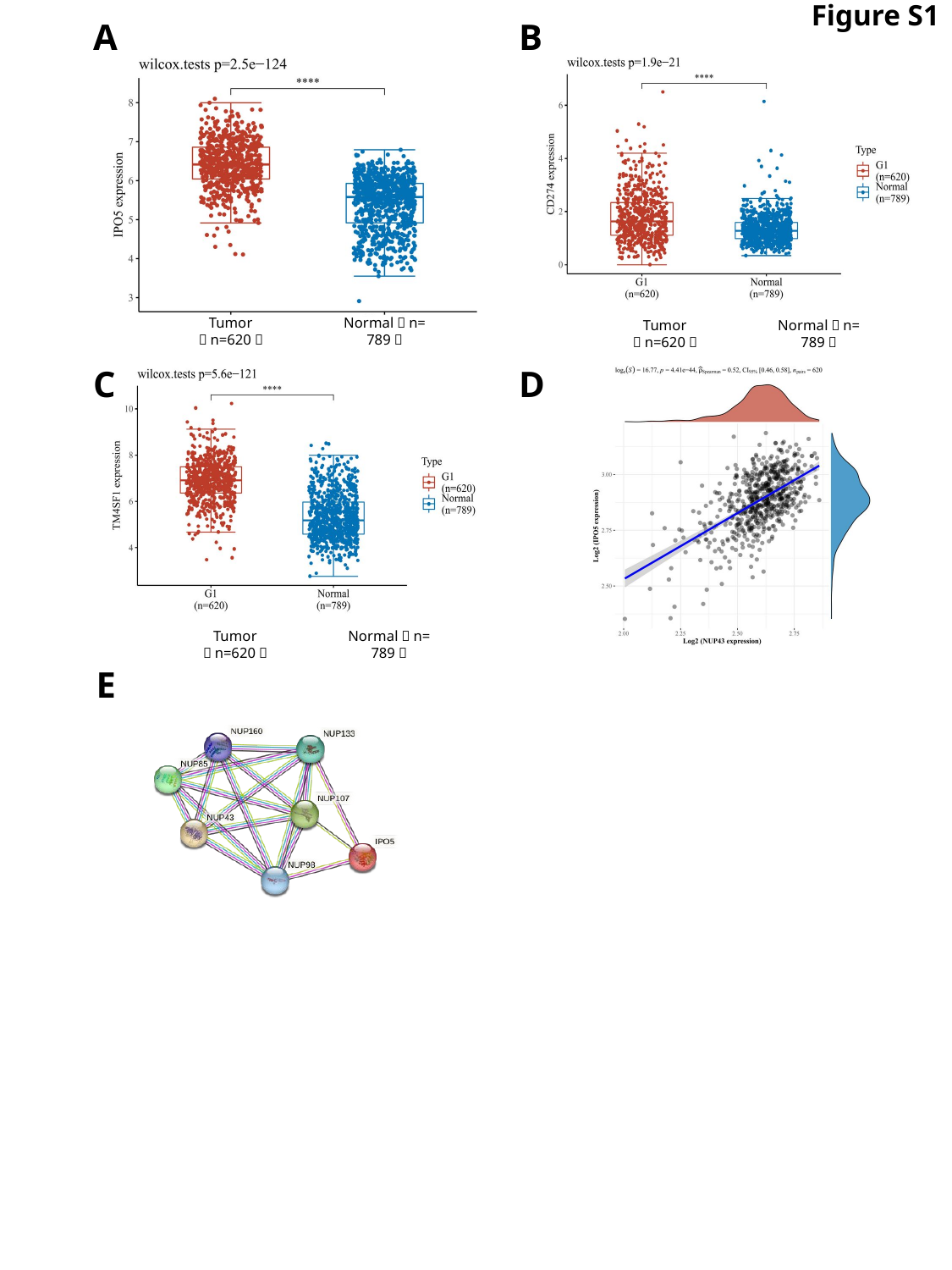

Figure S1
A
B
Tumor
（n=620）
Normal（n=789）
Tumor
（n=620）
Normal（n=789）
D
C
Tumor
（n=620）
Normal（n=789）
E

## Slide 2
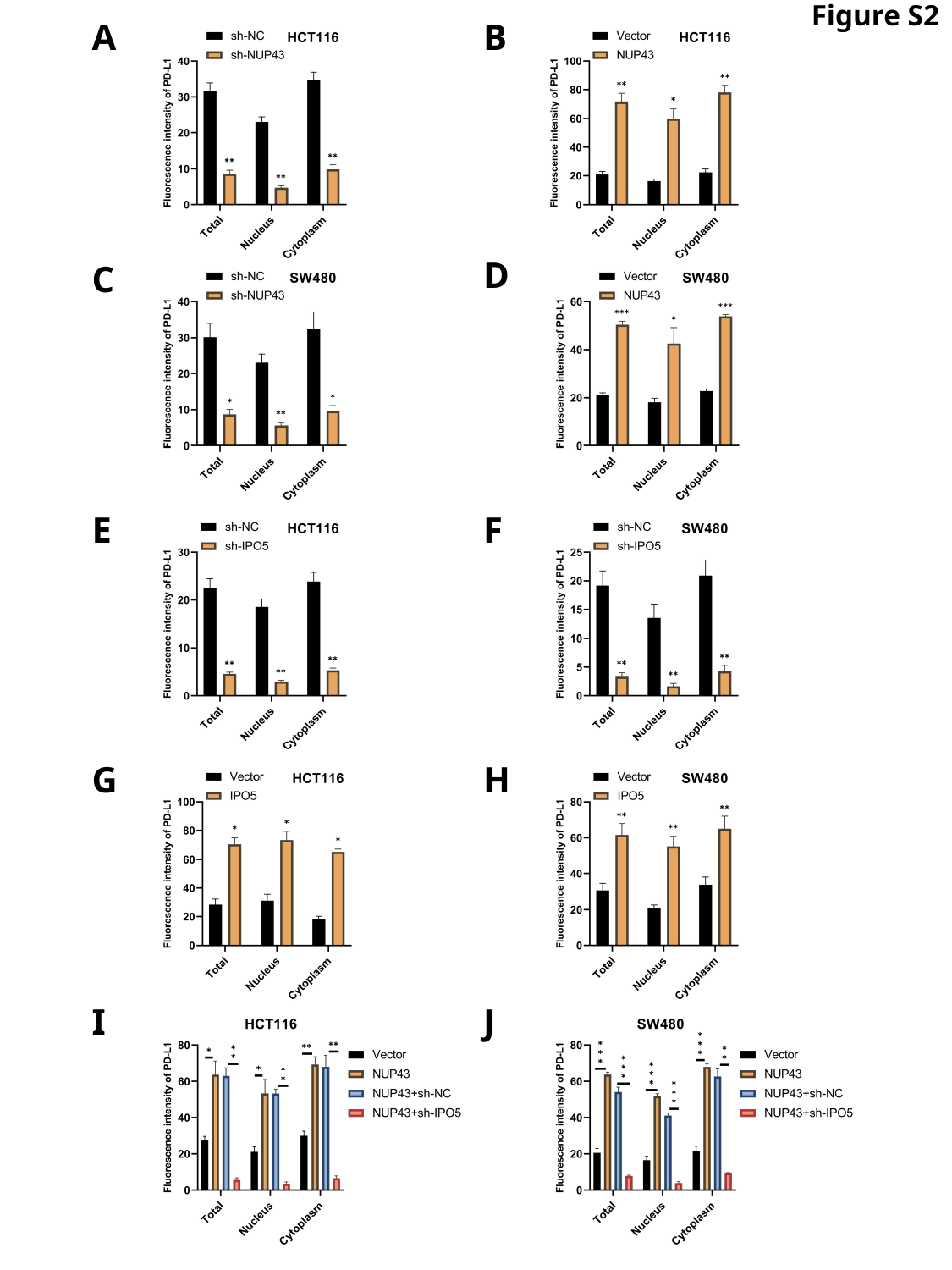

Figure S2
A
B
D
C
E
F
G
H
I
J

## Slide 3
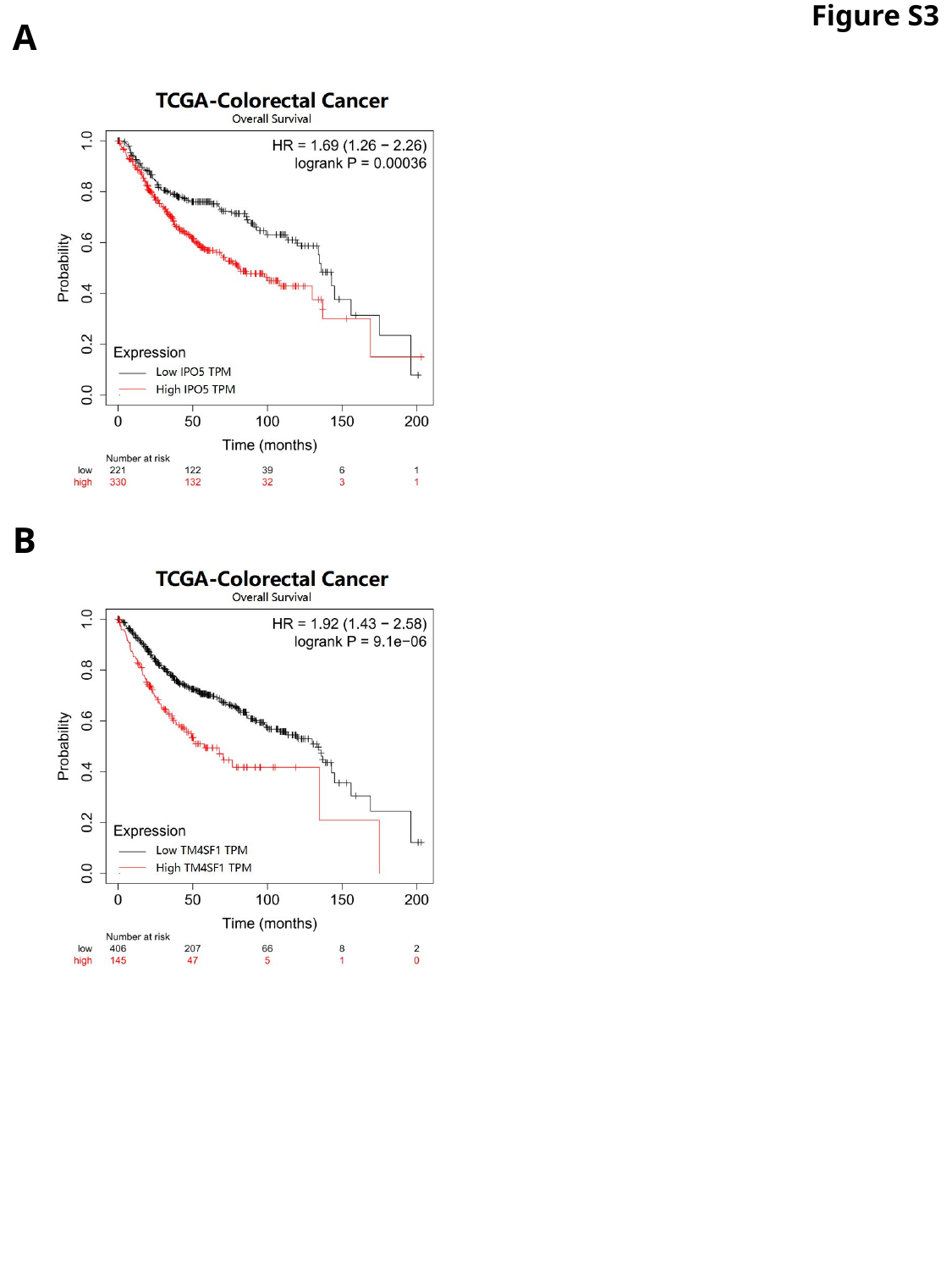

Figure S3
A
B

## Slide 4
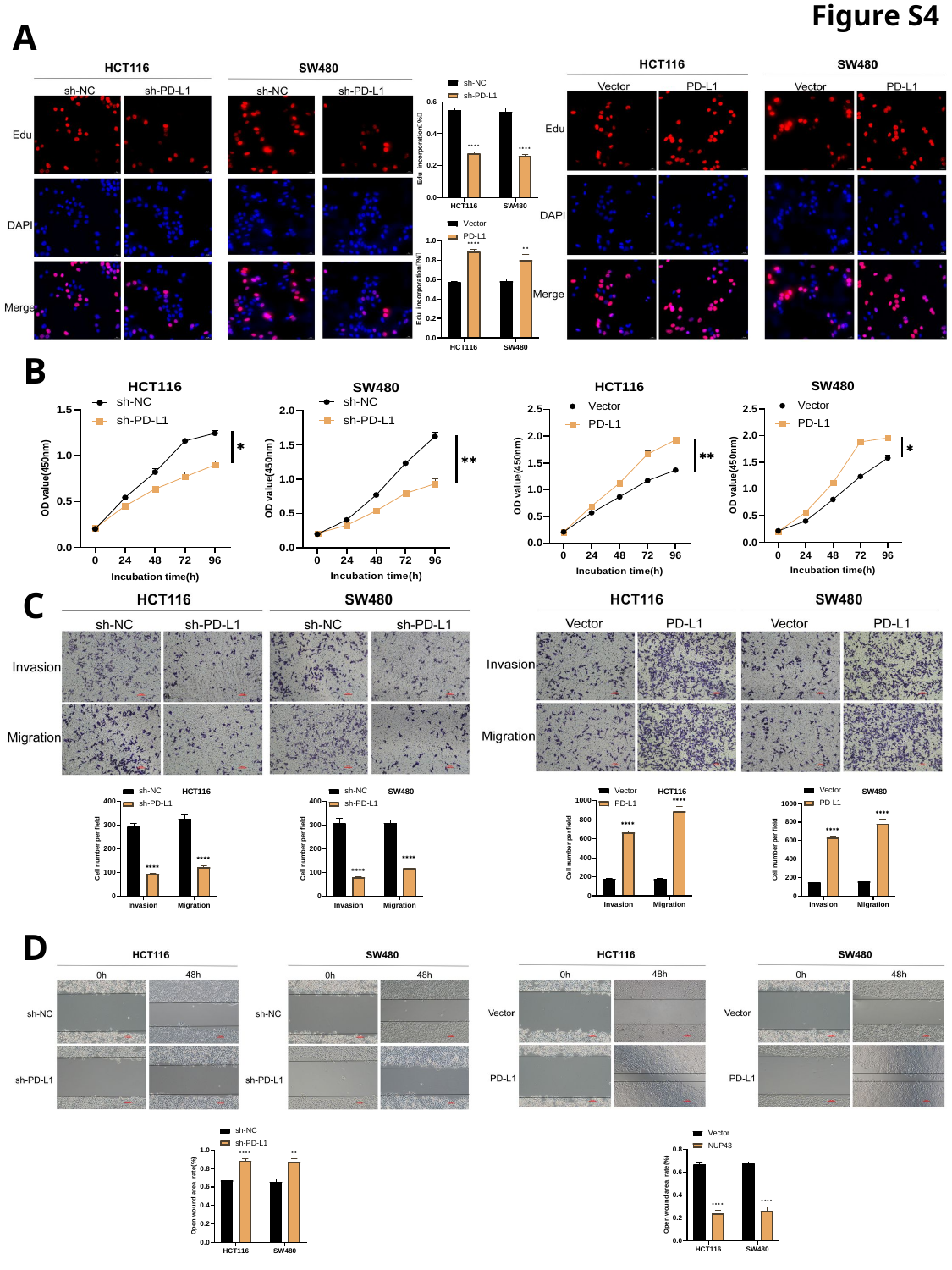

Figure S4
A
B
C
D

## Slide 5
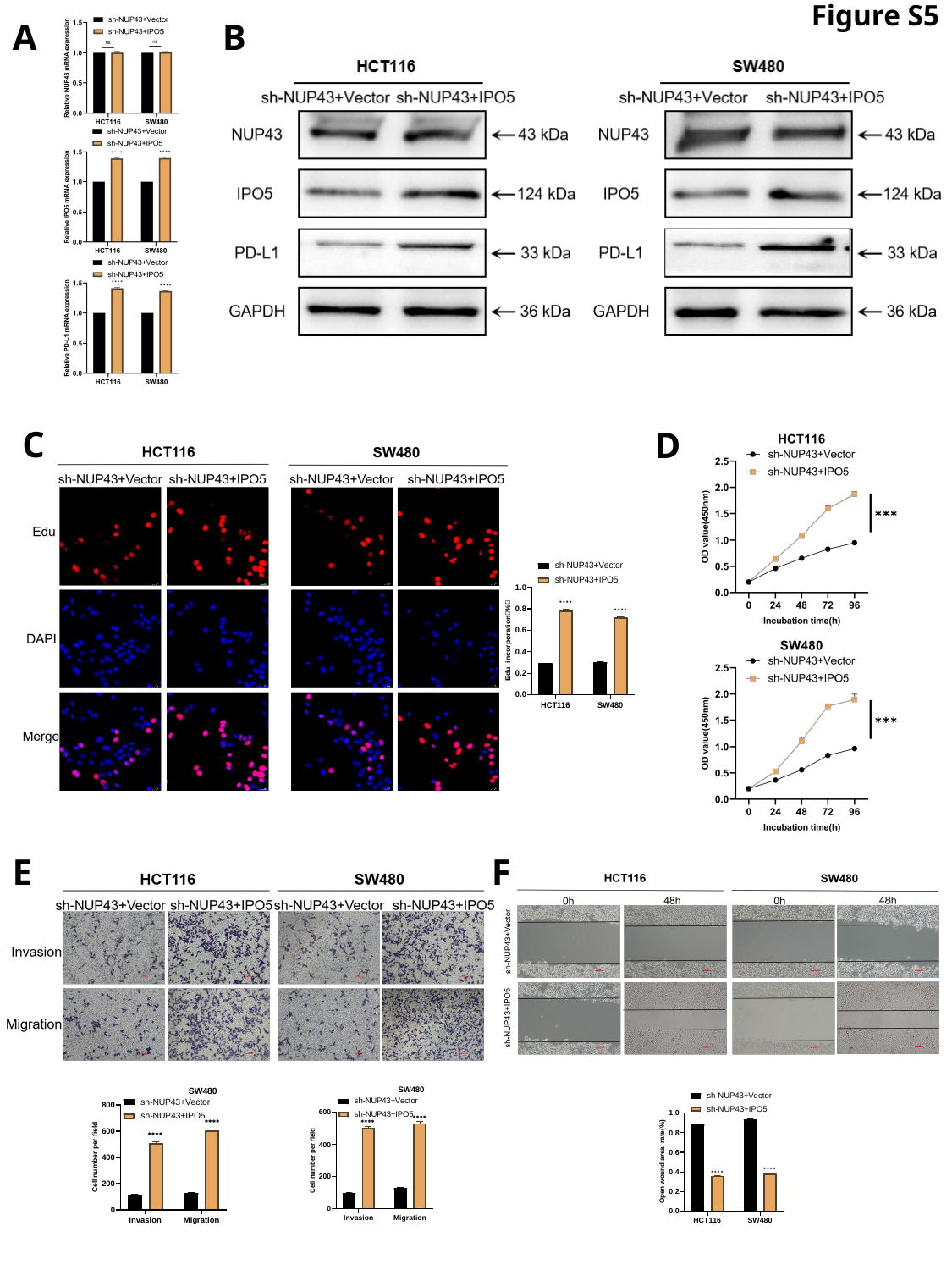

Figure S5
B
A
C
D
E
F

## Slide 6
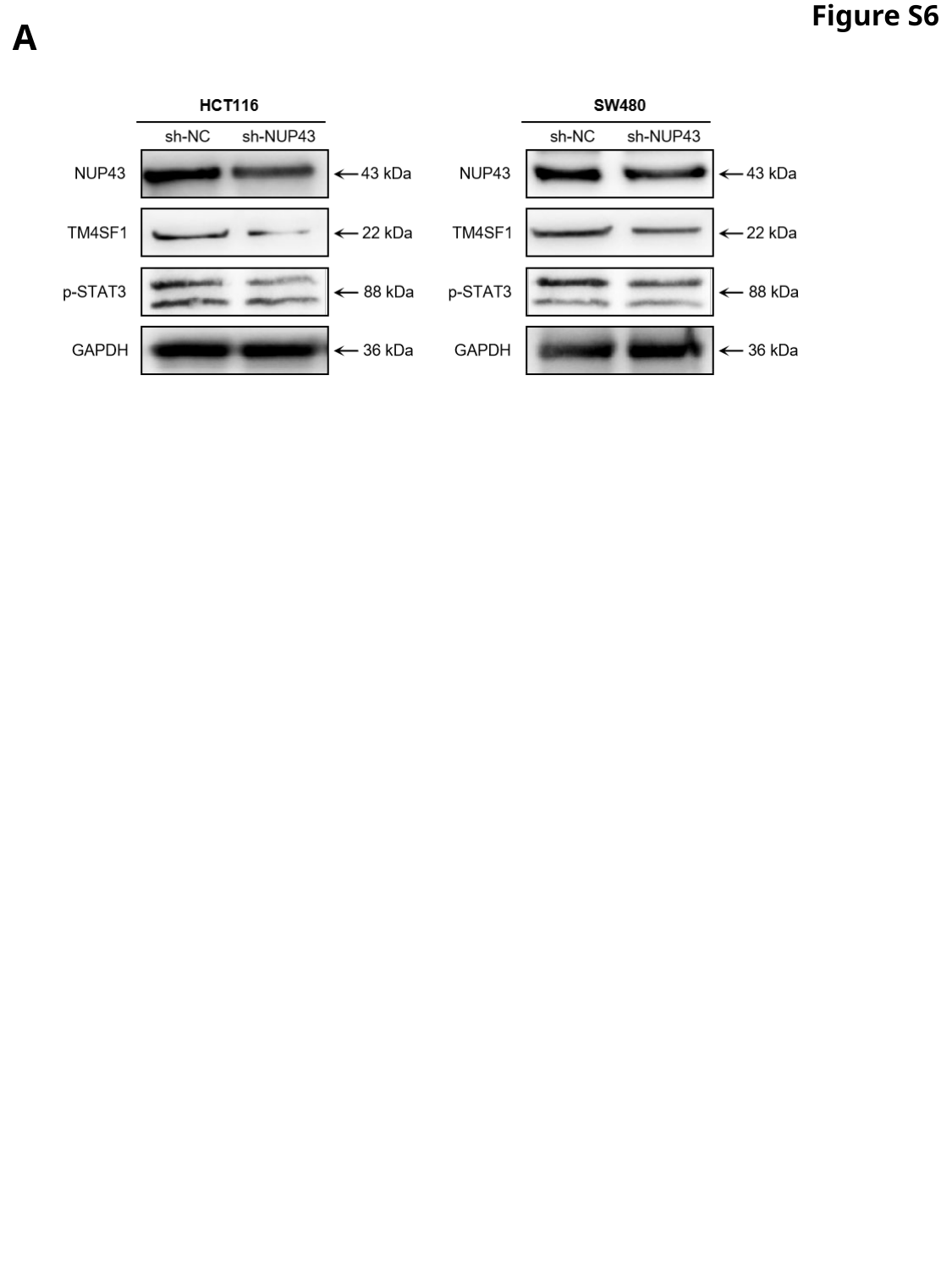

Figure S6
A
