# Supplementary material for: NUP43 promotes PD-L1/nPD-L1/PD-L1 feedback loop via TM4SF1/JAK/STAT3 pathway in colorectal cancer progression and metastatsis
Source: Cell Death Discov. 2024 May 18;10:241. doi: 10.1038/s41420-024-02025-z (PMC11102480; doi:10.1038/s41420-024-02025-z)
Supplement: Supplementary file 2 — Supplementary figure legends and Supplementary Tables [file 41420_2024_2025_MOESM2_ESM.docx]

**Supplementary figure legends:**

**Figure S1. Related bioinformatics analysis.** (A-B) Analysis of IPO5, PD-L1 (CD274), and TM4SF1 expression in both normal and CRC tissues was conducted using data from the TCGA database. (D-E) Performing a comprehensive investigation at the gene and protein levels to investigate the association between NUP43 and IPO5.

**Figure S2. Confocal microscopy analysis of cells in each group after PD-L1 immunofluorescence staining, data analysis of fluorescence intensity in each group.** *, P < 0.05; **, P < 0.01; ***, P < 0.001; ****, P < 0.0001.

**Figure S3. Analysis of survival time using the TCGA database.**

**Figure S4. PD-L1 enhances the growth and infiltration of CRC cells.** (A) The EdU assay is used to assess the proliferation capacity of CRC cells that have been transfected with sh-PD-L1/PD-L1. (B) A growth curve was constructed for cells that were transfected with sh-PD-L1/PD-L1 using the CCK-8 technique. (E) Transwell test is used to assess the invasive and metastatic abilities of CRC cells that have been transfected with sh-PD-L1/PD-L1. (F) A scratch experiment was performed to assess the invasive capacity of CRC cells that were transfected with sh-PD-L1/PD-L1. *, P < 0.05; **, P < 0.01; ***, P < 0.001; ****, P < 0.0001.

**Figure S5. Overexpression of IPO5 can rescue the phenotype of sh-NUP43 cells.** (A) qRT-PCR was used to determine the expression of NUP43, IPO5, and PD-L1 mRNA in cells from sh-NUP43+Vector and sh-NUP43+IPO5. (B) Western blot analysis was performed to examine the expression of NUP43, IPO5 and PD-L1 in each experimental group, including sh-NUP43+Vector and sh-NUP43+IPO5. (C) The EdU assay is used to assess the proliferation capacity of CRC cells that have been transfected with sh-NUP43+Vector/sh-NUP43+IPO5. (D) The growth curve of cells transfected with sh-NUP43+Vector/sh-NUP43+IPO5 was drawn according to the CCK-8 method. (E) Transwell test was used to assess the invasive and metastatic properties of CRC cells that were transfected with sh-NUP43+Vector/sh-NUP43+IPO5. (F) A scratch experiment was conducted to assess the invasive capacity of CRC cells that were transfected with sh-NUP43+Vector/sh-NUP43+IPO5. *, P < 0.05; **, P < 0.01; ***, P < 0.001; ****, P < 0.0001.

**Figure S6. Depletion of NUP43 reduces TM4SF1 expression and thus inhibits p-STAT3 signaling.** (A) The Western blot analysis reveals the levels of NUP43, TM4SF1, and phospho-STAT3 expression in the group of sh-NC and sh-NUP43.

**Table S1. Primer sequences used in RT-qPCR analysis.**

| Target | Forward primer (5’-3’) | Reverse primer (5’-3’) |
| --- | --- | --- |
| IPO5 | CTGCTGAAGAGGCTAGACAAATG | TCTGCCGCAATATCACAAACTT |
| GAPDH | GGAGCGAGATCCCTCCAAAAT | GGCTGTTGTCATACTTCTCATGG |
| PD-L1 | GGTGCCGACTACAAGCGAAT | GGTGACTGGATCCACAACCAA |
| NUP43 | GTCCTGAAATAGTGTCCGTGGGT | TATGTGCTGCTGGTTTGGGTGT |
| TM4SF1 | GTGGAGGAAATTGTGGCTGT | CGTTCATGGTGATCCAACTG |
| STAT3 | GGAGGAGTTGCAGCAAAAAG | TGTGTTTGTGCCCAGAATGT |

**Table S2. Nucleotide sequences used for knockdown**

| NUP43 shRNA 1 | TTTCCTTCACCATCCAAATAA |
| --- | --- |
| NUP43 shRNA 2 | TGGAGAGGATGGTCGAATAAA |
| NUP43 shRNA 3 | CTGAGATTCTTACTGTAAATT |
| pdl1 shRNA 1 | ACCCGAACCTGTCAGTGATAT |
| pdl1 shRNA 2 | GACTGTCAAAGCTACCTATAA |
| pdl1 shRNA 3 | GTTTGACATCCCAGATGAAAT |
| TM4SF1 shRNA 1 | CCATCAGTTGGGAGTTGAAGA |
| TM4SF1 shRNA 2 | GCCAAGTGTCCGAGATGCAAC |
| TM4SF1 shRNA 3 | GCTCTCACCAACAGCAATATT |
| IPO5 shRNA 1 | CCTGCCGATGATCAAGGAACA |
| IPO5 shRNA 2 | GCTTCATTTAAGTATGCAGAA |
| IPO5 shRNA 3 | GCCCATGATTAAGGAACACAT |
| STAT3 shRNA 1 | ATTCATAATCTCTTGGGTGAA |
| STAT3 shRNA 2 | AATGCAGGATCTGAATGGAAC |
| STAT3 shRNA 3 | AAGAGGGTCTCGGAAATTTAA |
