# Supplementary material for: NUP43 promotes PD-L1/nPD-L1/PD-L1 feedback loop via TM4SF1/JAK/STAT3 pathway in colorectal cancer progression and metastatsis
Source: Cell Death Discov. 2024 May 18;10:241. doi: 10.1038/s41420-024-02025-z (PMC11102480; doi:10.1038/s41420-024-02025-z)

Figure 3.B

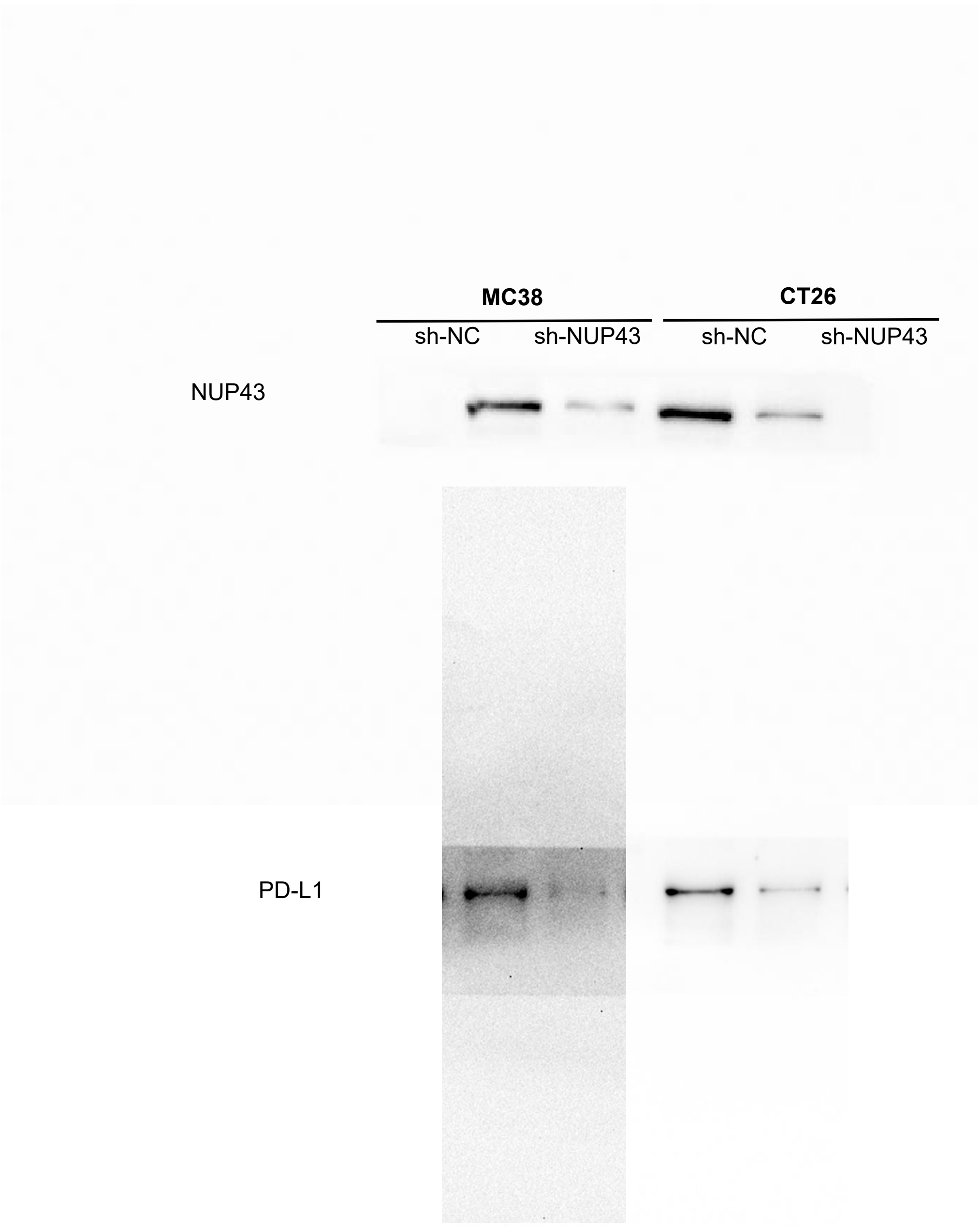
MC38
CT26
sh-NC
sh-NUP43
sh-NC
sh-NUP43
NUP43
PD-L1

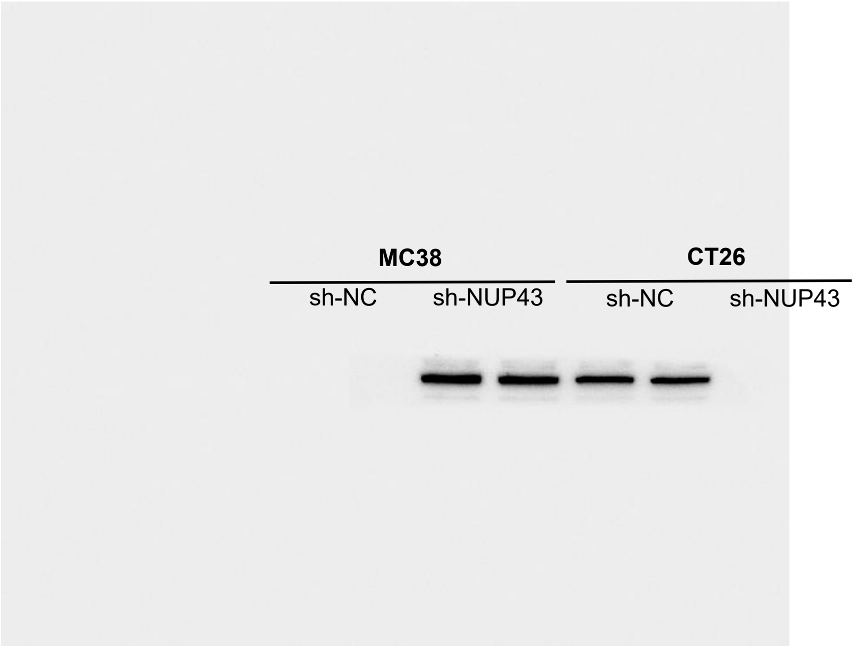
MC38
CT26
sh-NC
sh-NUP43
sh-NC
sh-NUP43

Figure 4.B

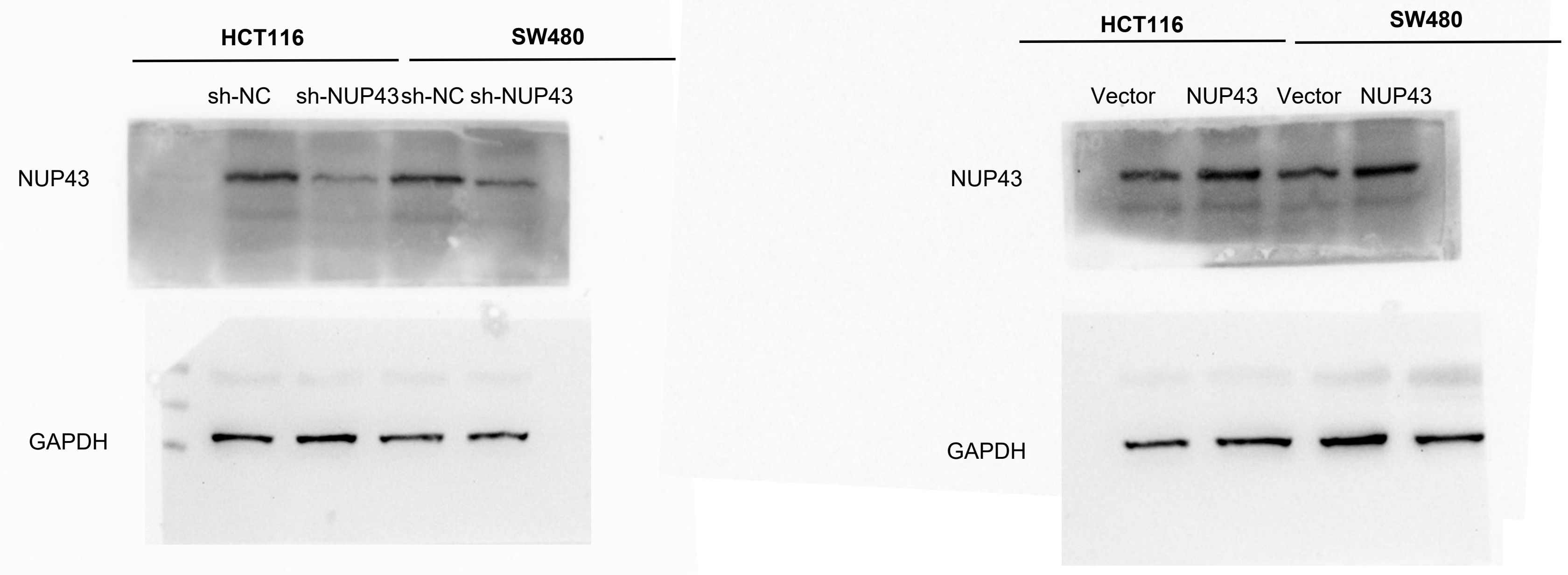
HCT116
SW480
sh-NC
sh-NUP43
sh-NC
sh-NUP43
NUP43
GAPDH
HCT116
SW480
Vector
NUP43
Vector
NUP43
NUP43
GAPDH

Figure 5.B

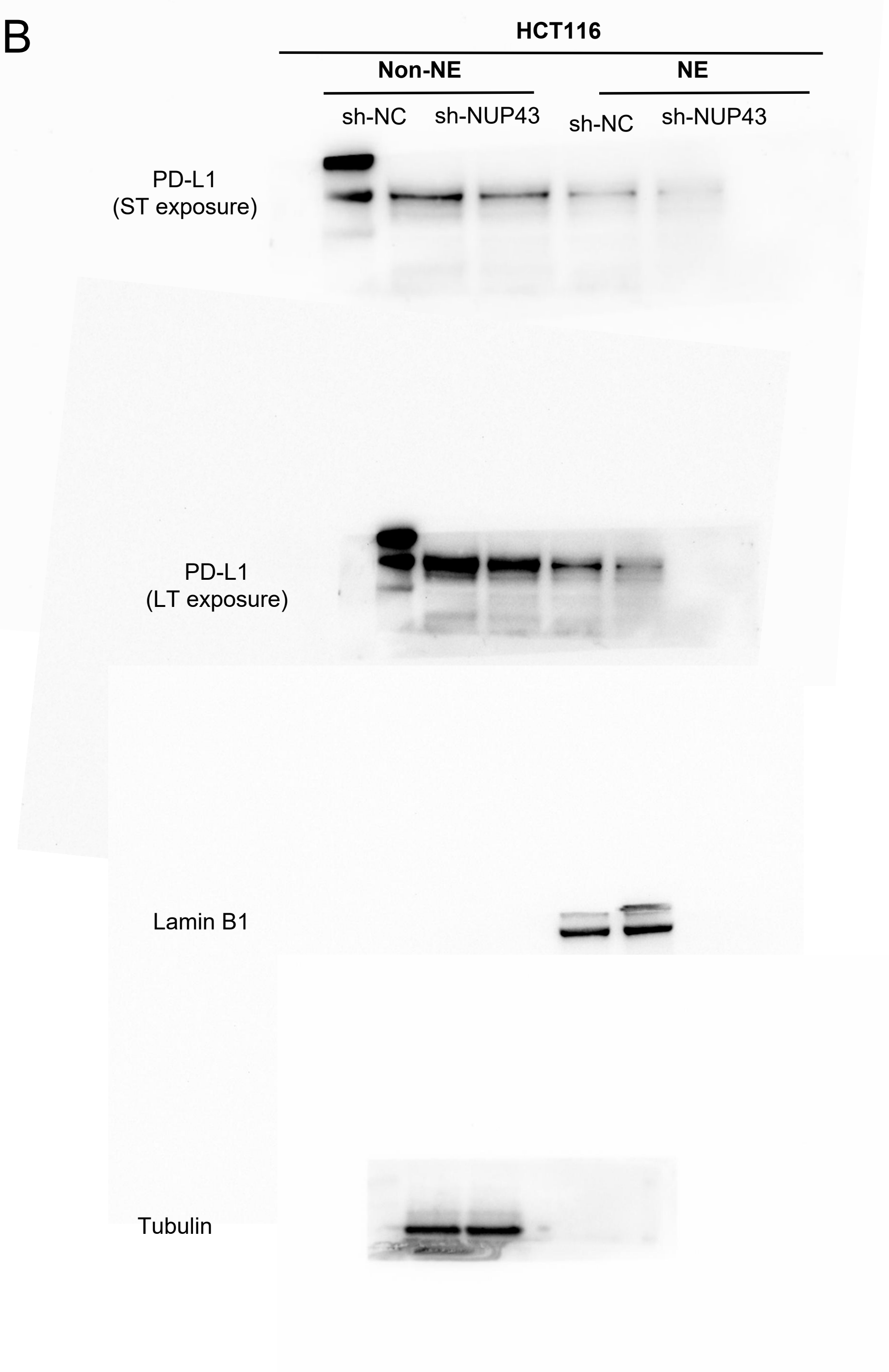
HCT116
Non-NE
NE
sh-NC
sh-NUP43
sh-NC
sh-NUP43
PD-L1
(ST exposure)
PD-L1
(LT exposure)
Lamin B1
Tubulin

Figure 5.B

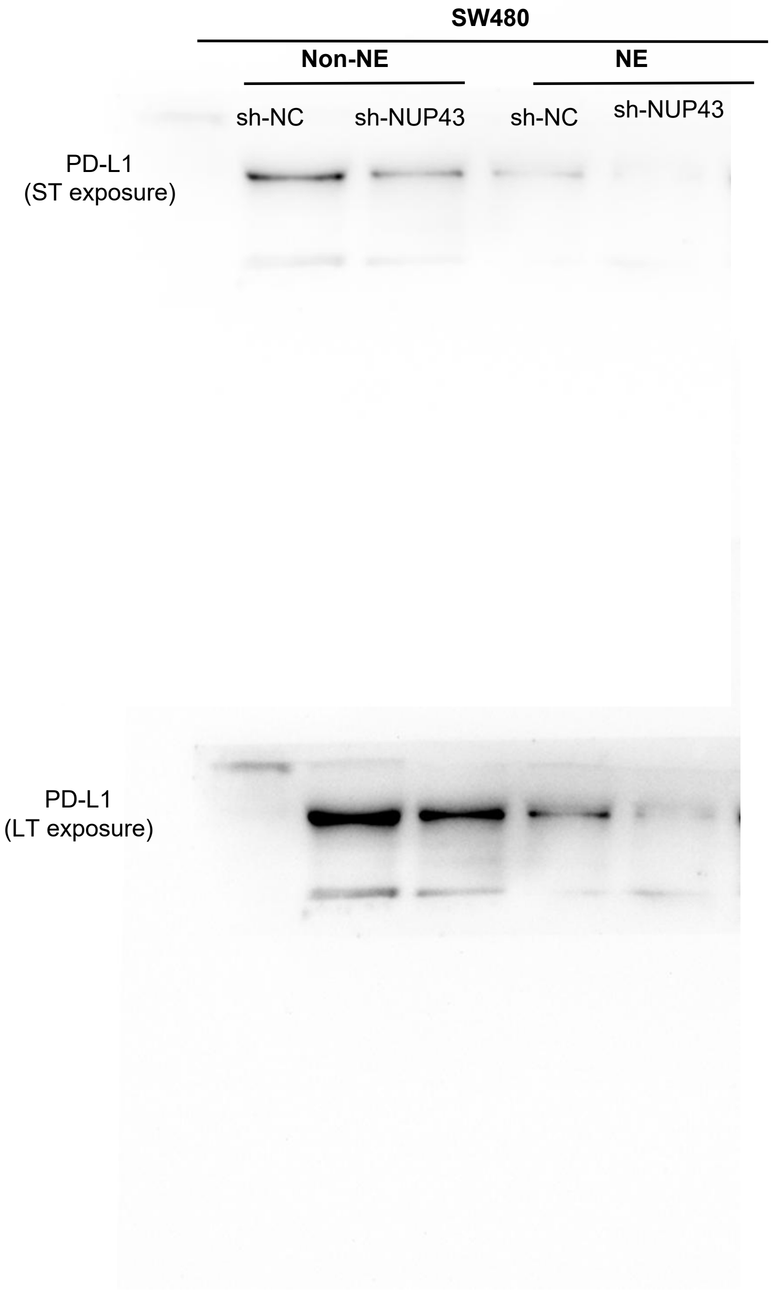
SW480
Non-NE
NE
sh-NC
sh-NUP43
sh-NC
sh-NUP43
PD-L1
(ST exposure)
PD-L1
(LT exposure)

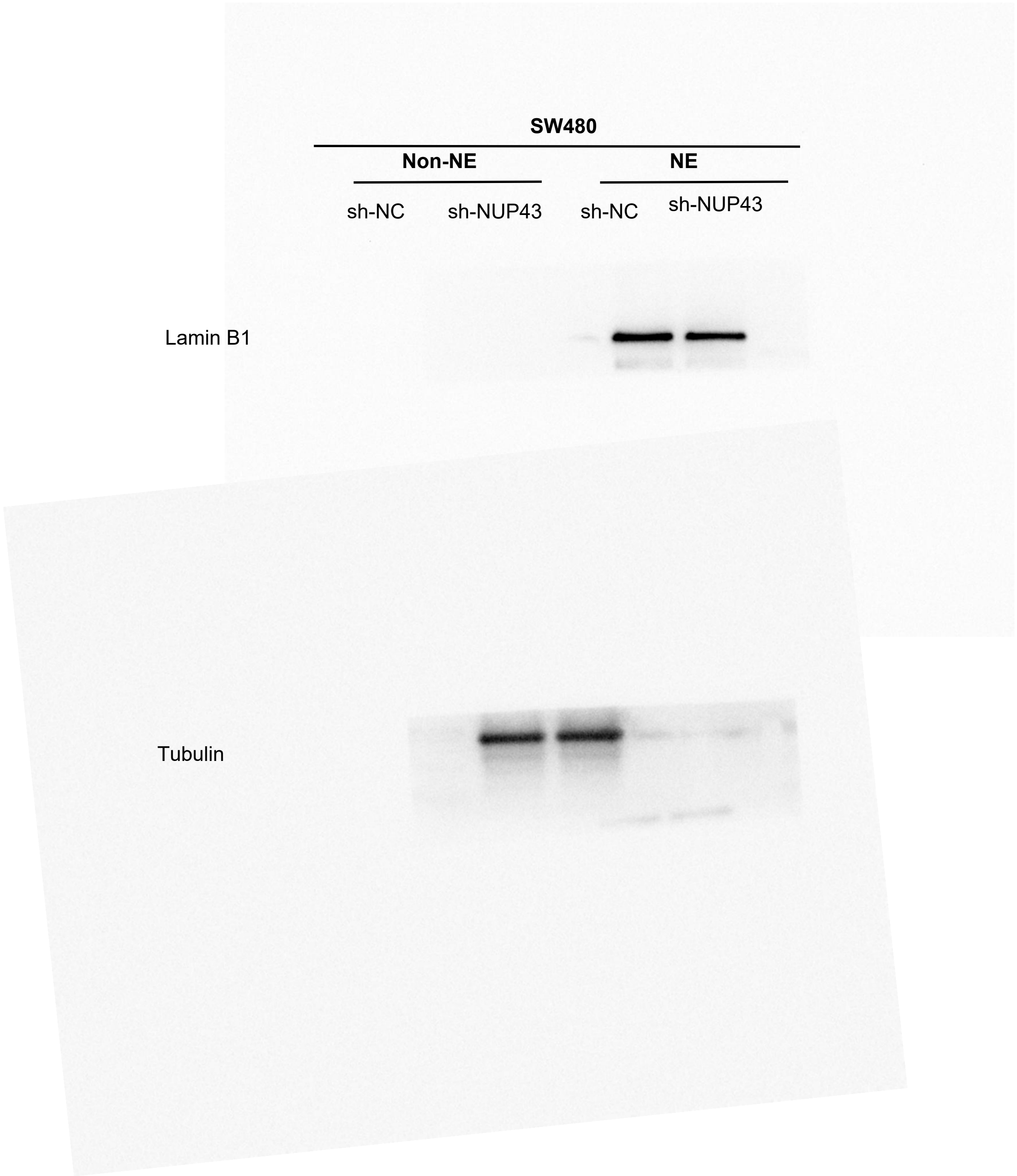
SW480
Non-NE
NE
sh-NC
sh-NUP43
sh-NC
sh-NUP43
Lamin B1
Tubulin

Figure 5.C

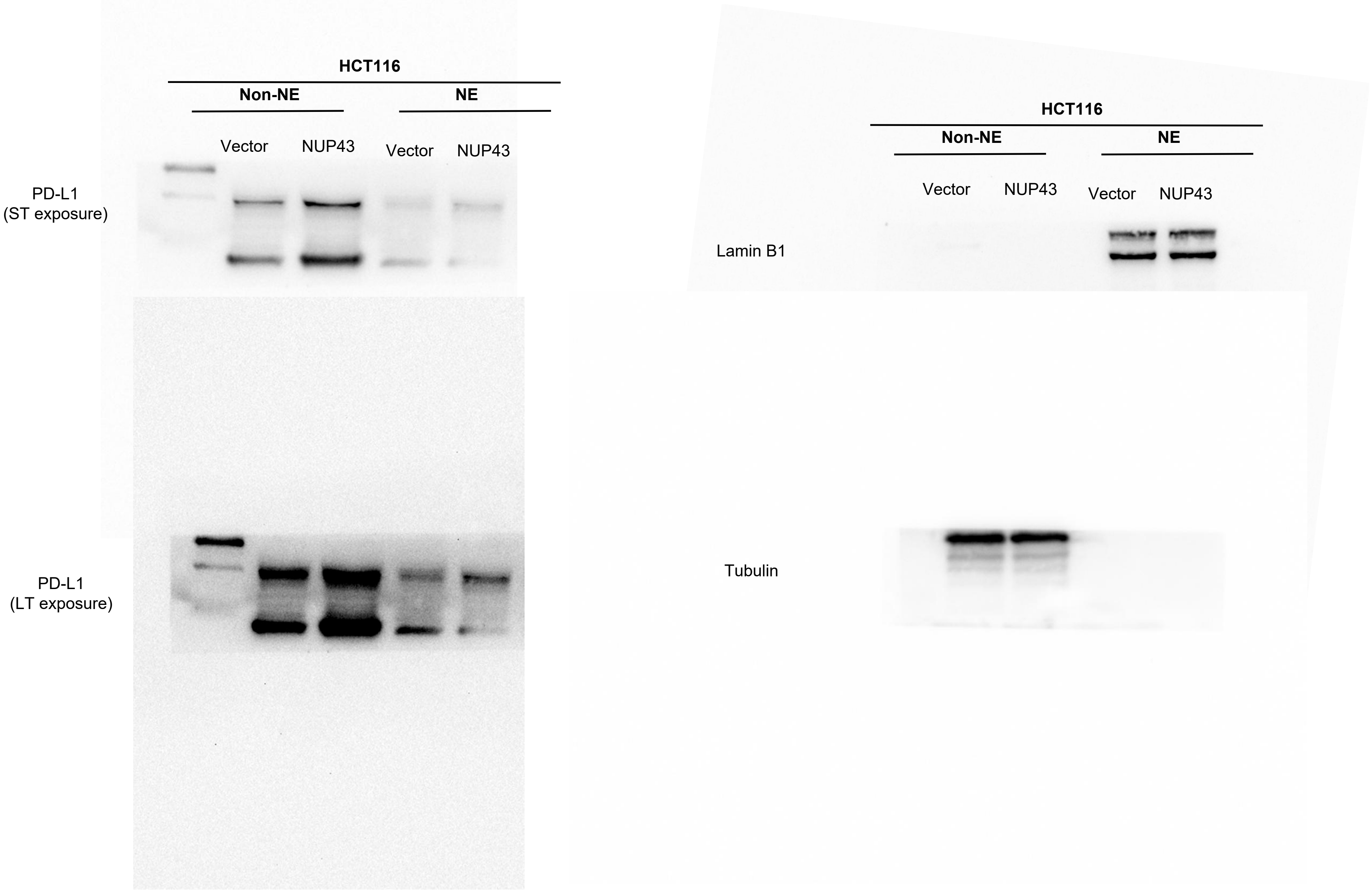
HCT116
Non-NE
NE
Vector
NUP43
Vector
NUP43
PD-L1
(ST exposure)
PD-L1
(LT exposure)
HCT116
Non-NE
NE
Vector
NUP43
Vector
NUP43
Lamin B1
Tubulin

Figure 5.C

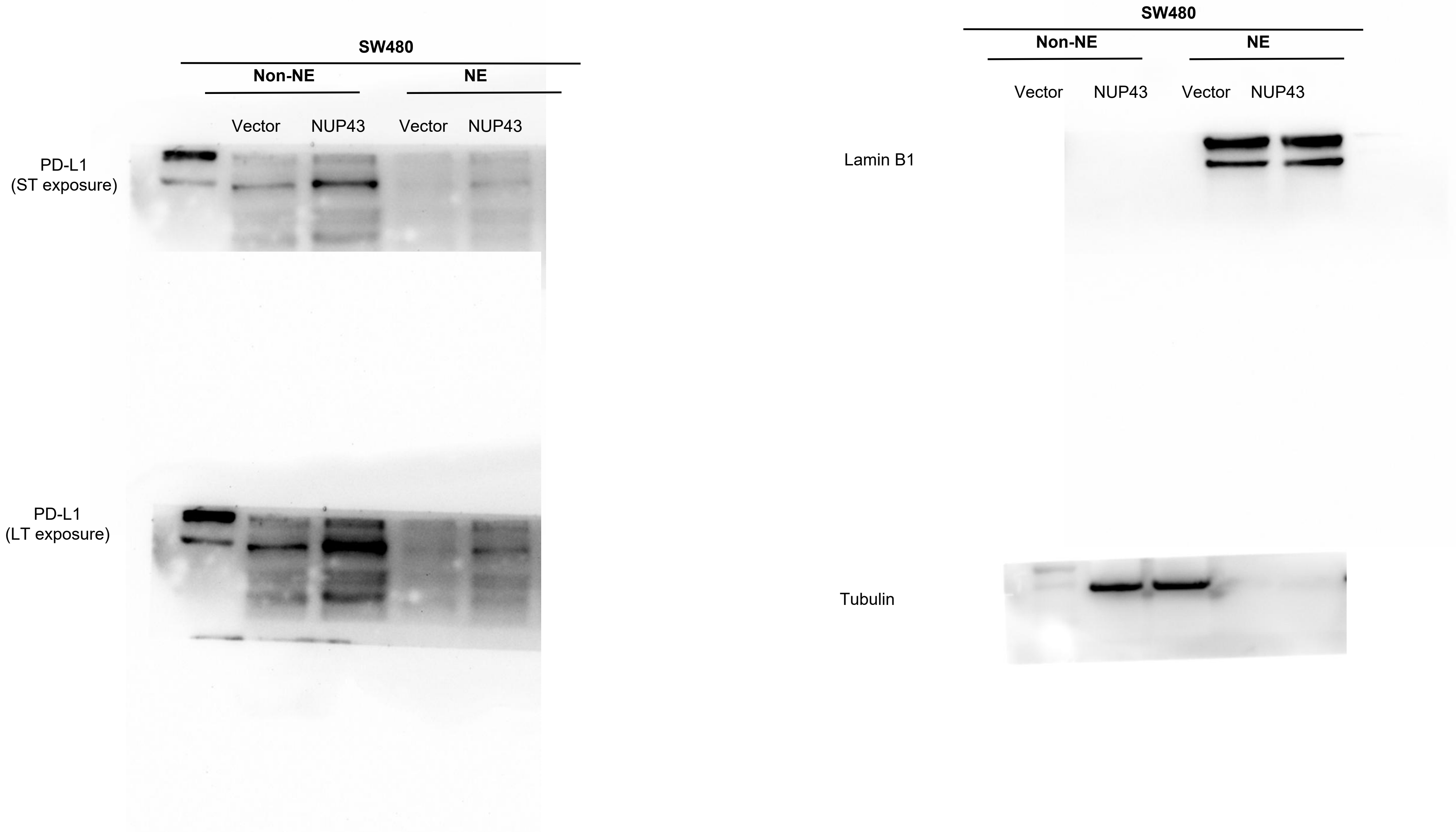

SW480
Non-NE
NE
Vector
NUP43
Vector
NUP43
PD-L1
(ST exposure)
PD-L1
(LT exposure)
SW480
Non-NE
NE
Vector
NUP43
Vector
NUP43
Lamin B1
Tubulin

Figure 6.F&G

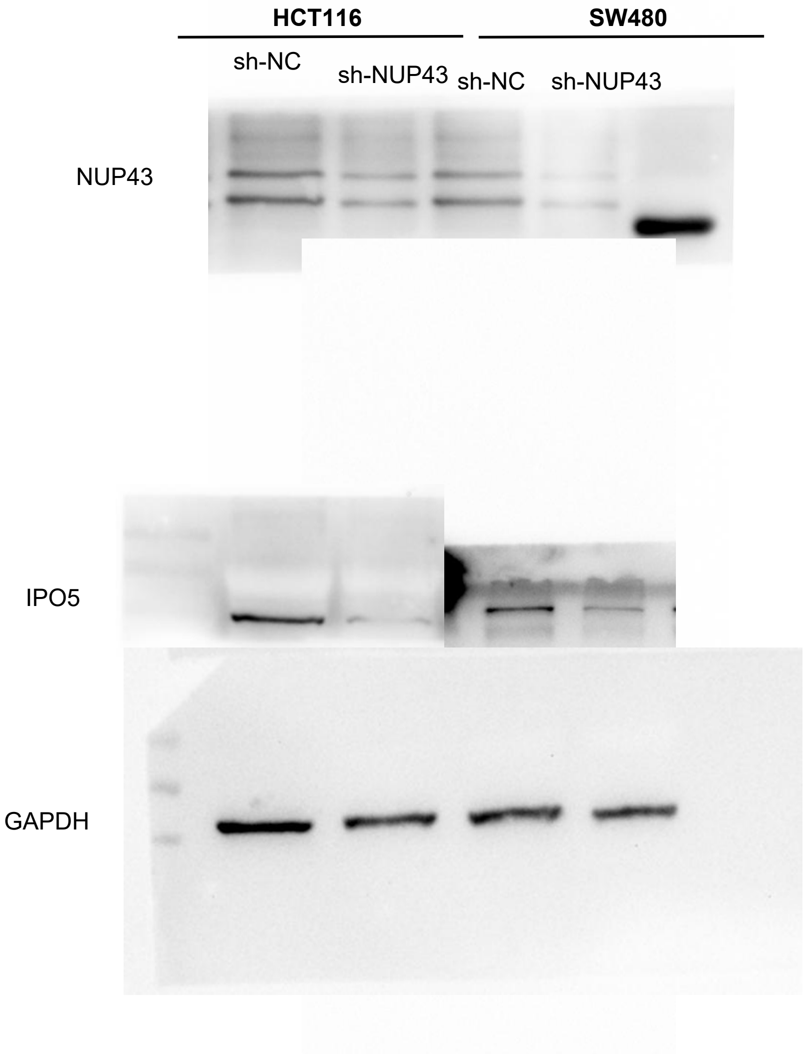
HCT116
SW480
sh-NC
sh-NUP43
sh-NC
sh-NUP43
NUP43
IPO5
GAPDH

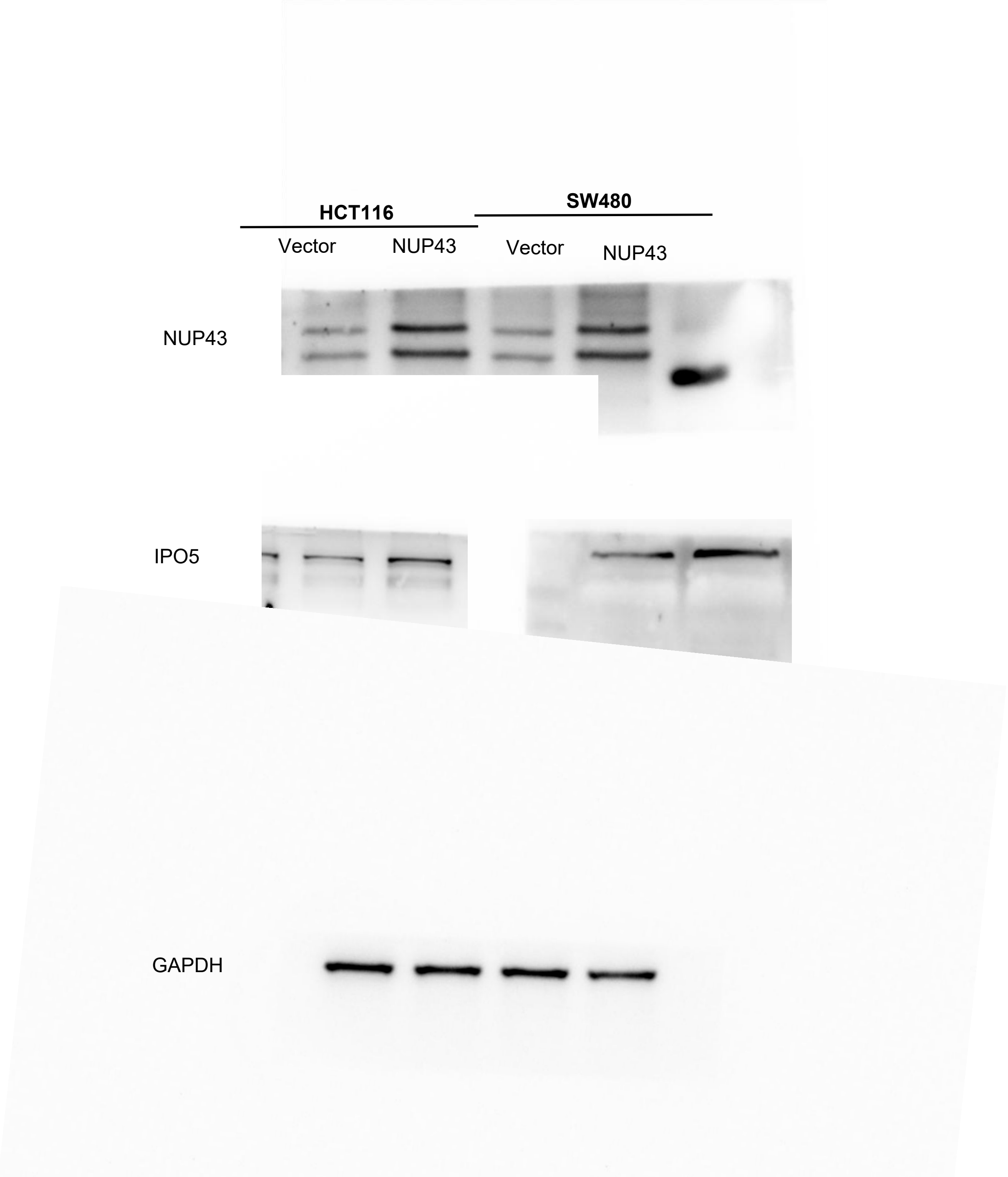
HCT116
SW480
Vector
NUP43
Vector
NUP43
NUP43
IPO5
GAPDH

Figure 6.H

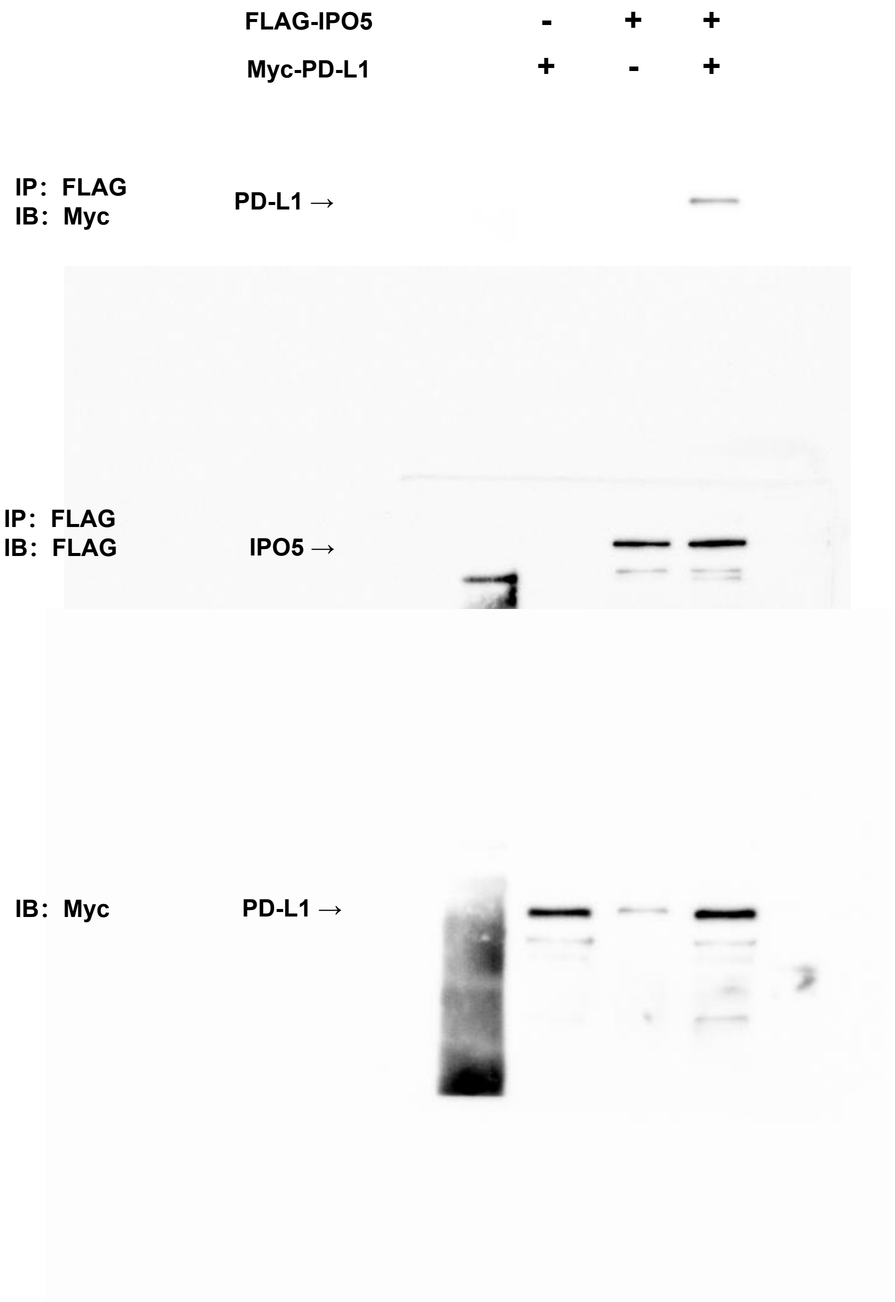
FLAG-IPO5 - + +
Myc-PD-L1 + - +
IP: FLAG
IB: Myc
PD-L1 →
IP: FLAG
IB: FLAG
IPO5 →
IB: Myc
PD-L1 →

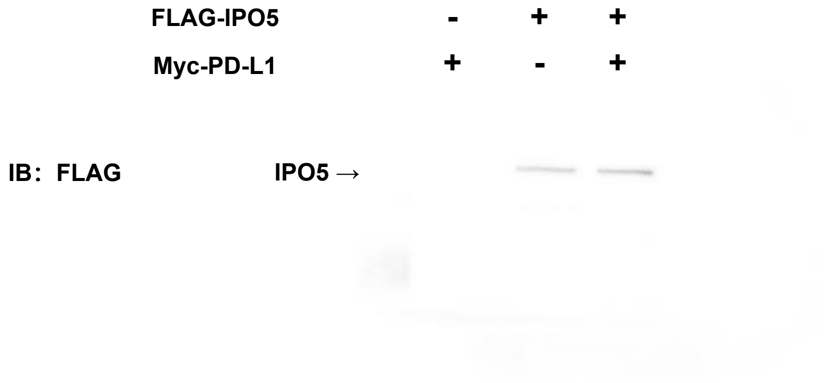
FLAG-IPO5 - + +
Myc-PD-L1 + - +
IB: FLAG
IPO5 →

Figure 7.B

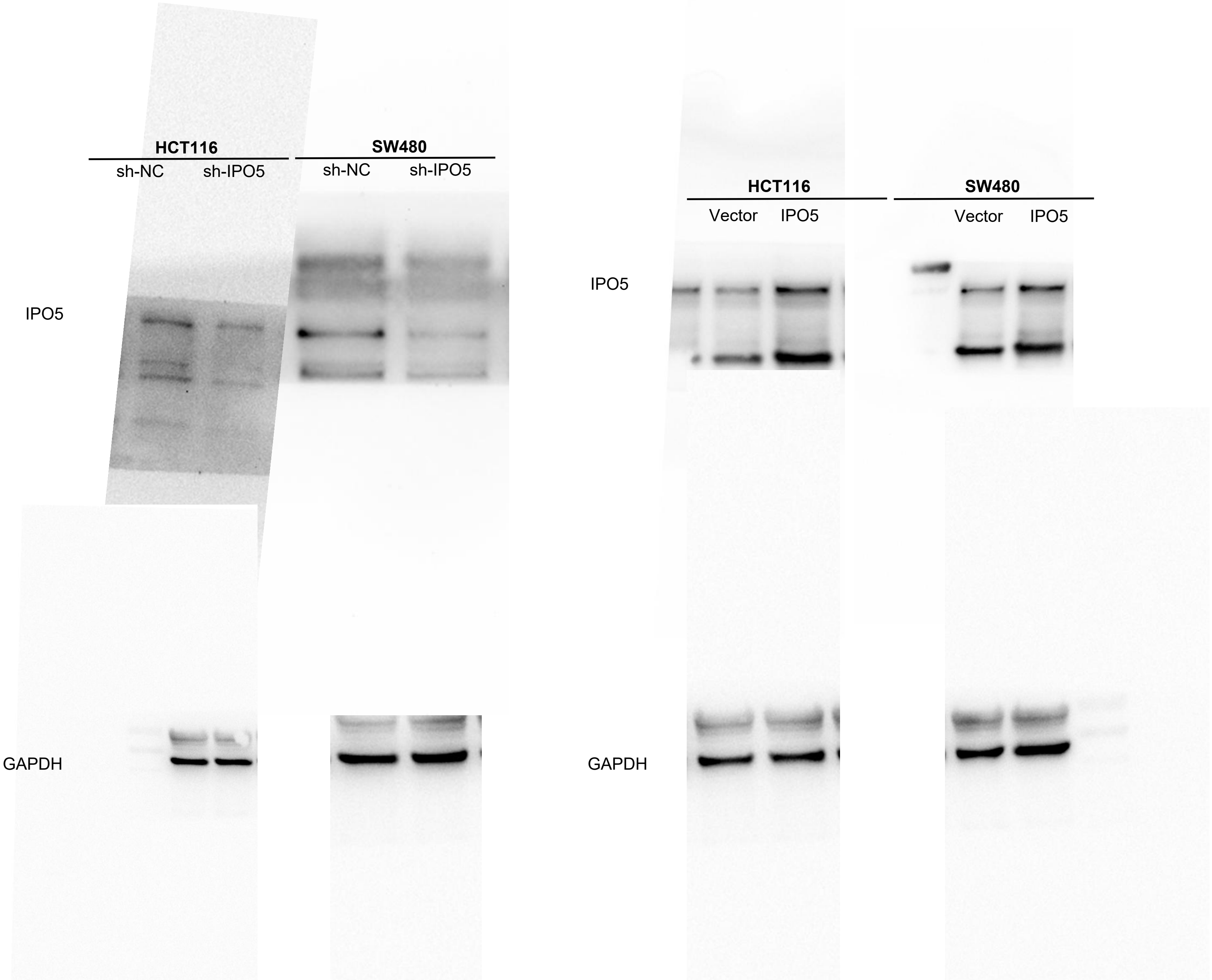

HCT116
sh-NC
sh-IPO5
SW480
sh-NC
sh-IPO5
IPO5
GAPDH
HCT116
Vector
IPO5
SW480
Vector
IPO5
IPO5
GAPDH

Figure 7.C

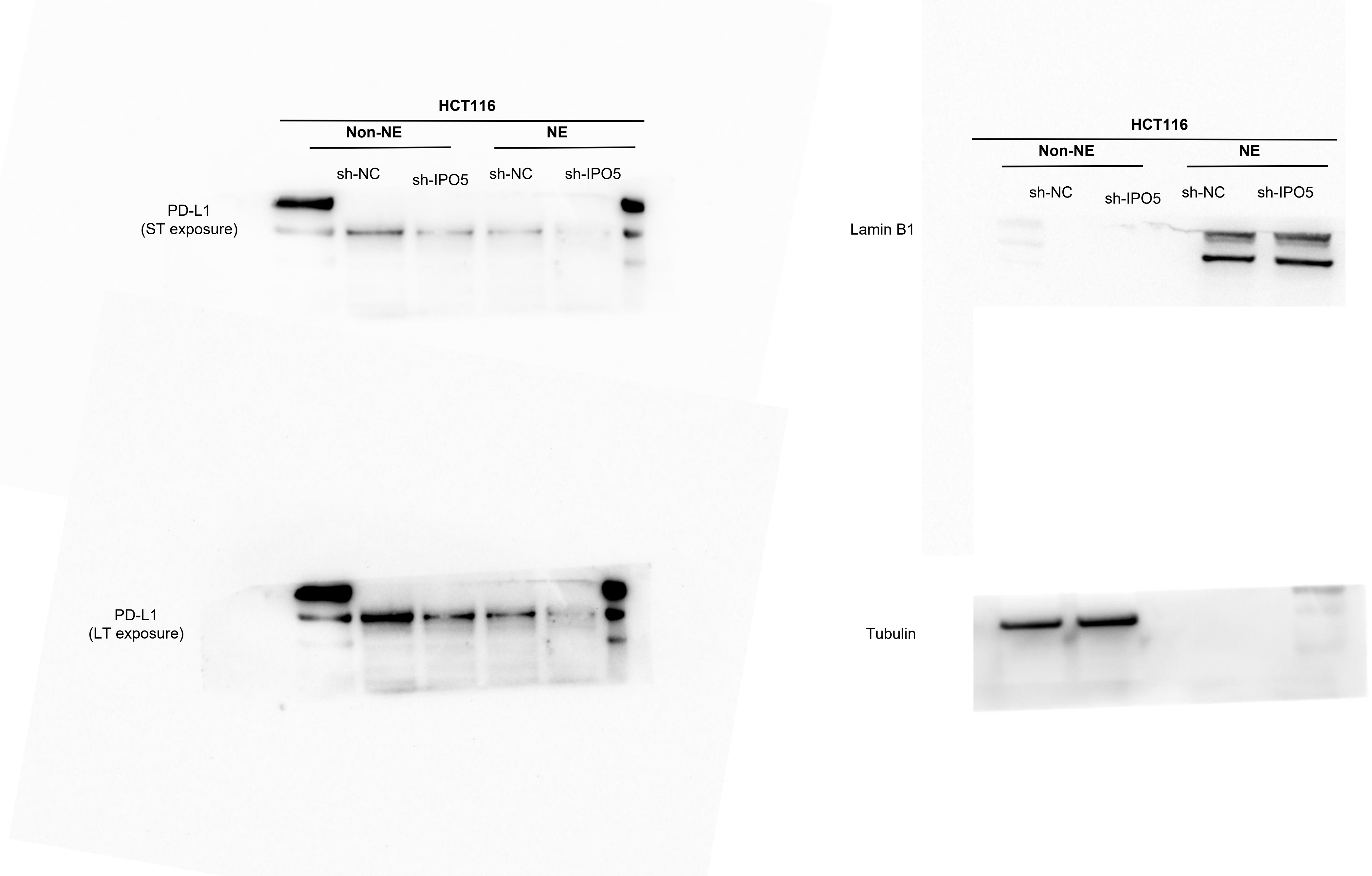
HCT116
Non-NE
NE
sh-NC
sh-IPO5
sh-NC
sh-IPO5
PD-L1
(ST exposure)
PD-L1
(LT exposure)
HCT116
Non-NE
NE
sh-NC
sh-IPO5
sh-NC
sh-IPO5
Lamin B1
Tubulin

Figure 7.C

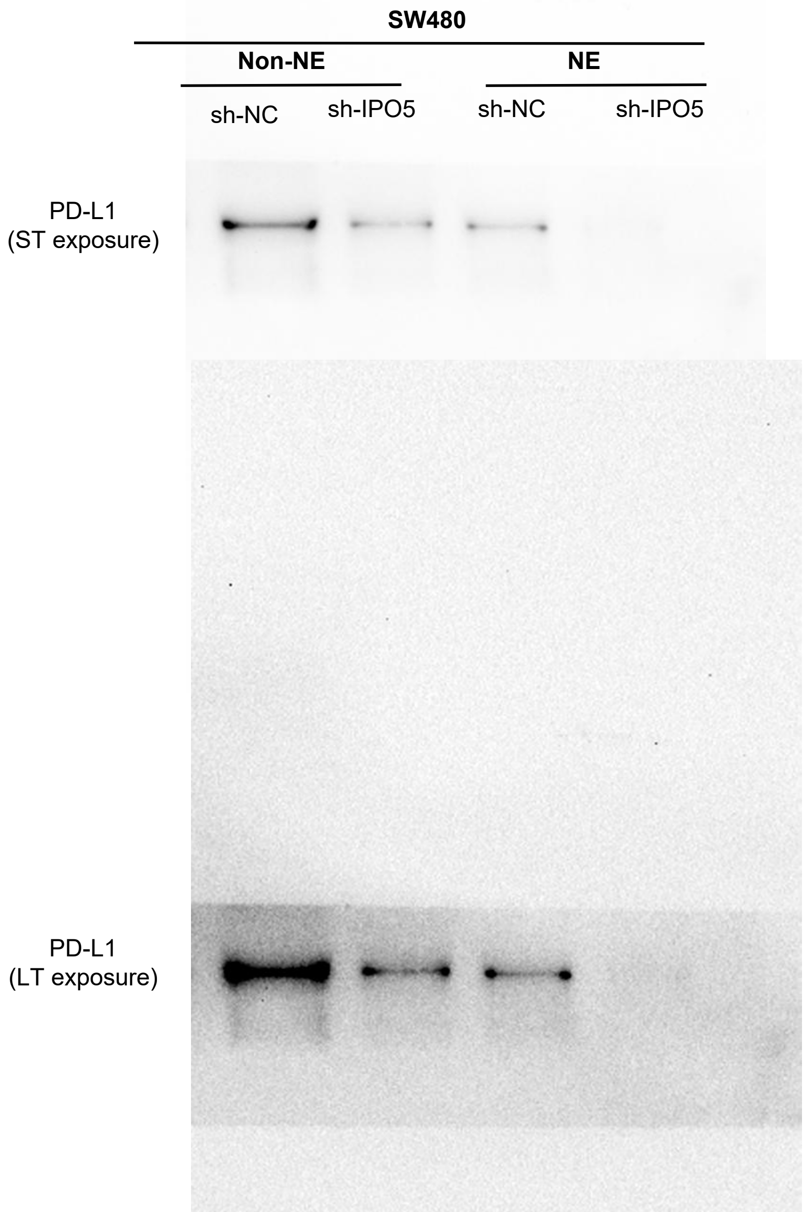

SW480
Non-NE
NE
sh-NC
sh-IPO5
sh-NC
sh-IPO5
PD-L1
(ST exposure)
PD-L1
(LT exposure)

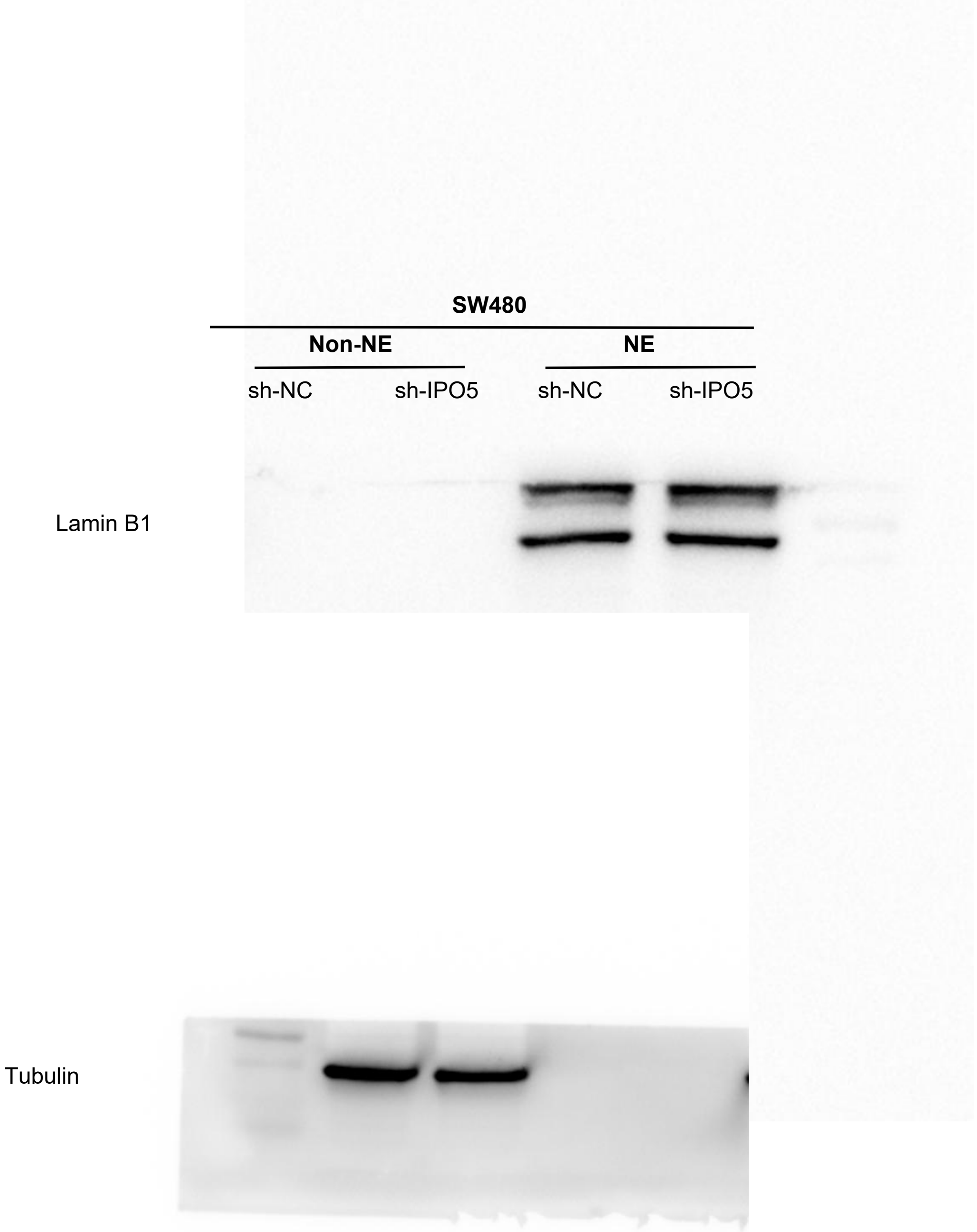

SW480
Non-NE
NE
sh-NC
sh-IPO5
sh-NC
sh-IPO5
Lamin B1
Tubulin

Figure 7.D

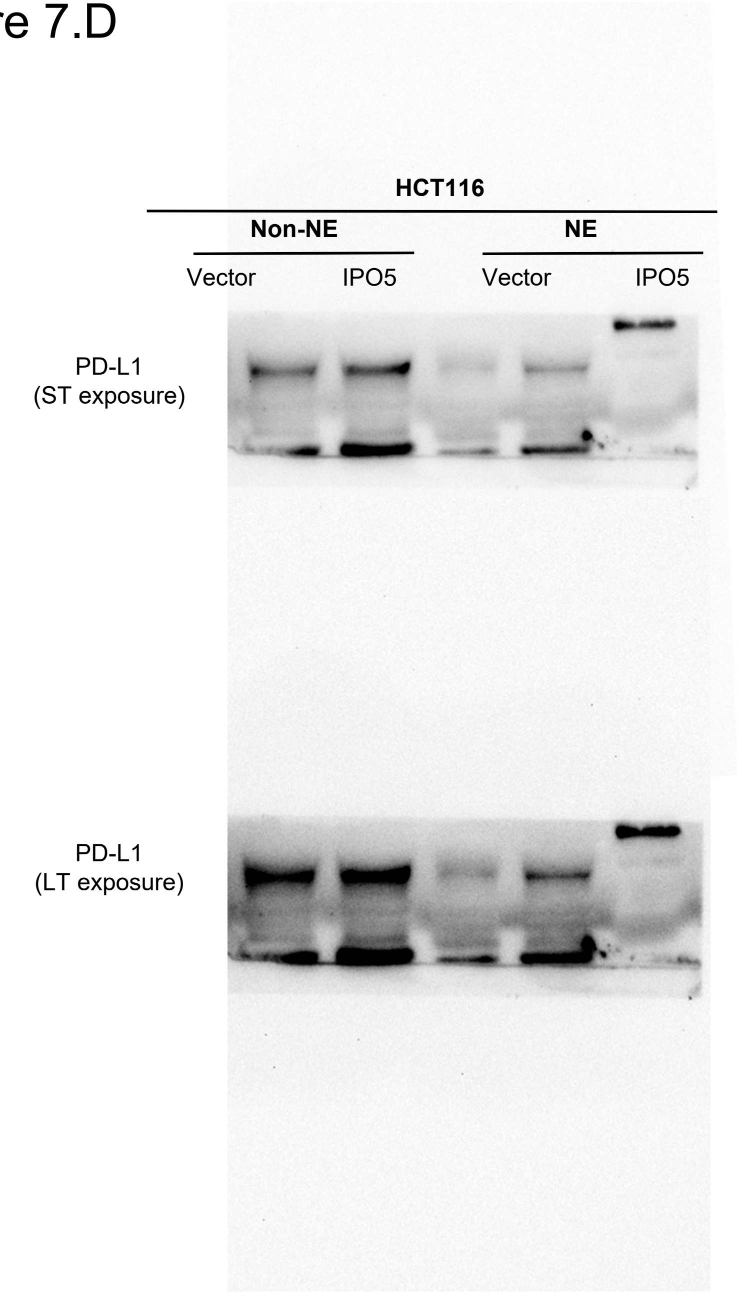
HCT116
Non-NE
NE
Vector
IPO5
Vector
IPO5
PD-L1
(ST exposure)
PD-L1
(LT exposure)

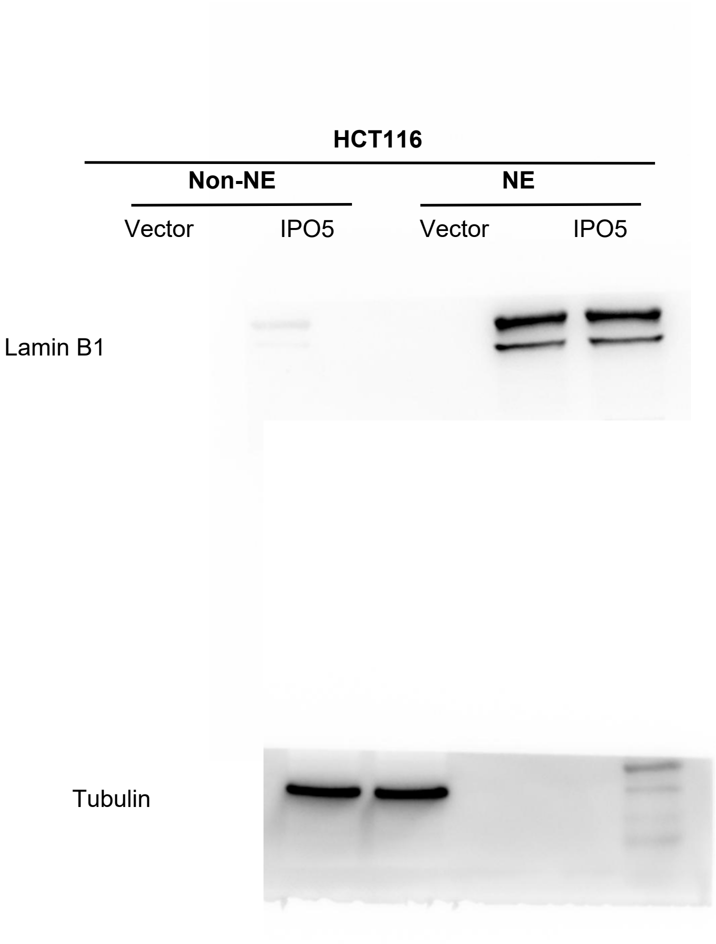
HCT116
Non-NE
NE
Vector
IPO5
Vector
IPO5
Lamin B1
Tubulin

Figure 7.D

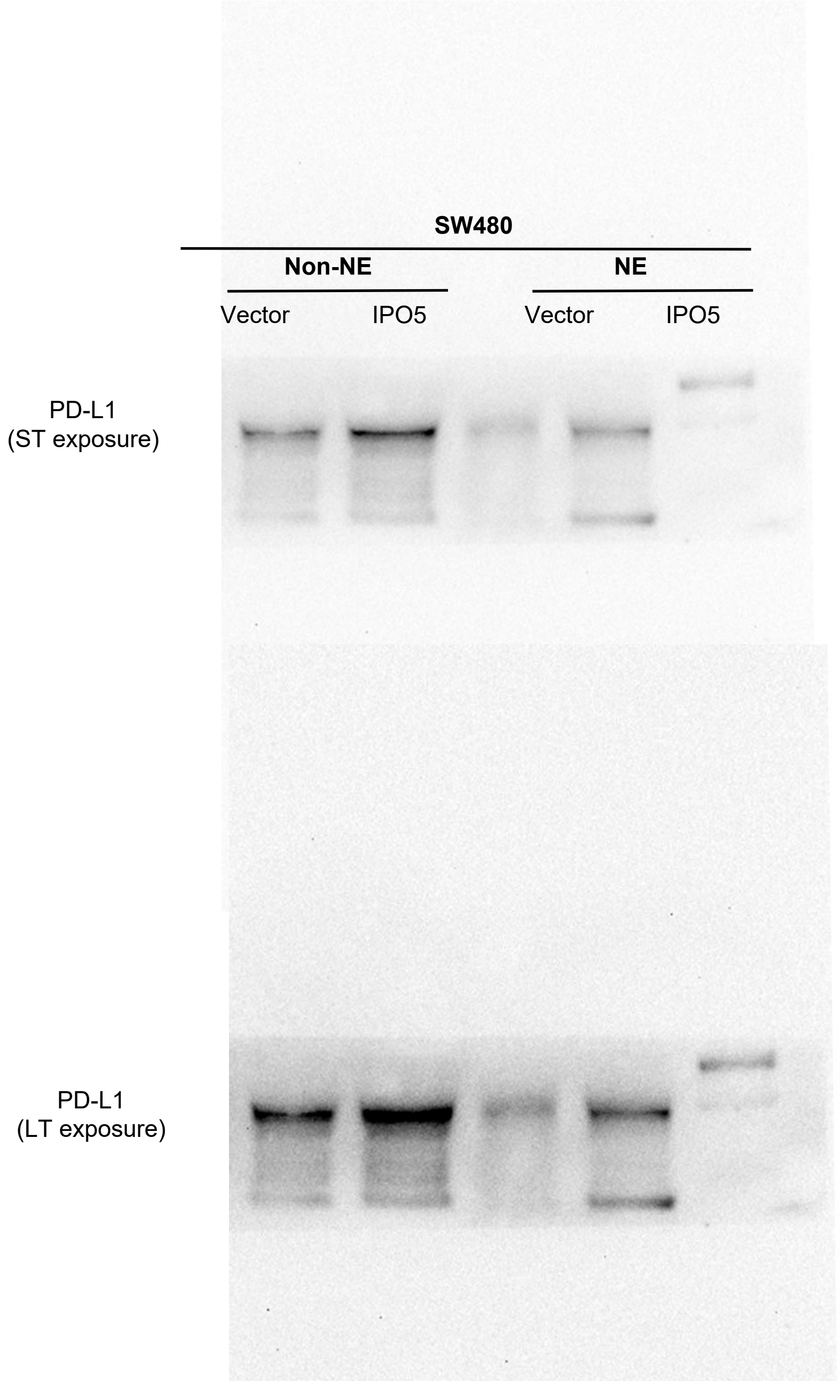

SW480
Non-NE
NE
Vector
IPO5
Vector
IPO5
PD-L1
(ST exposure)
PD-L1
(LT exposure)

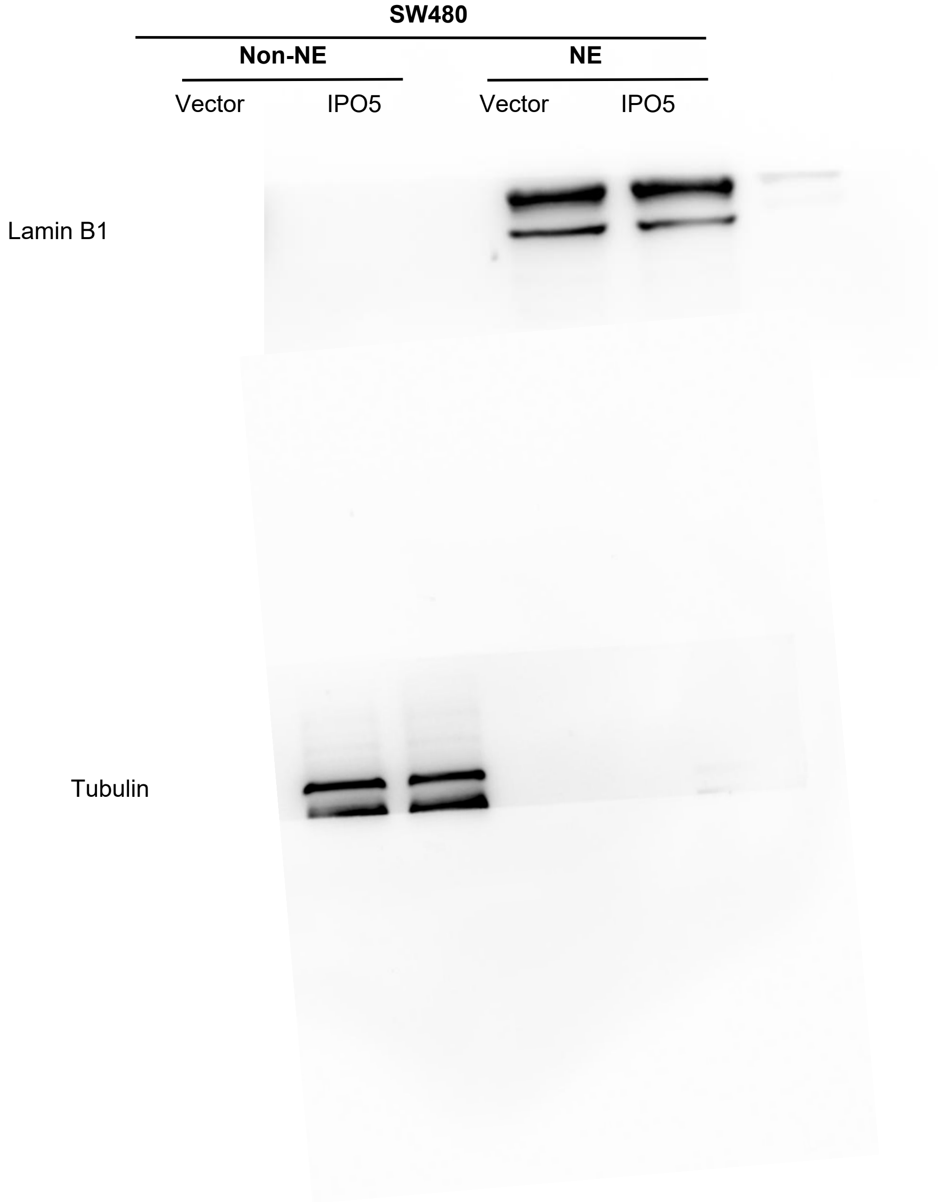

SW480
Non-NE
NE
Vector
IPO5
Vector
IPO5
Lamin B1
Tubulin

Figure 7.H

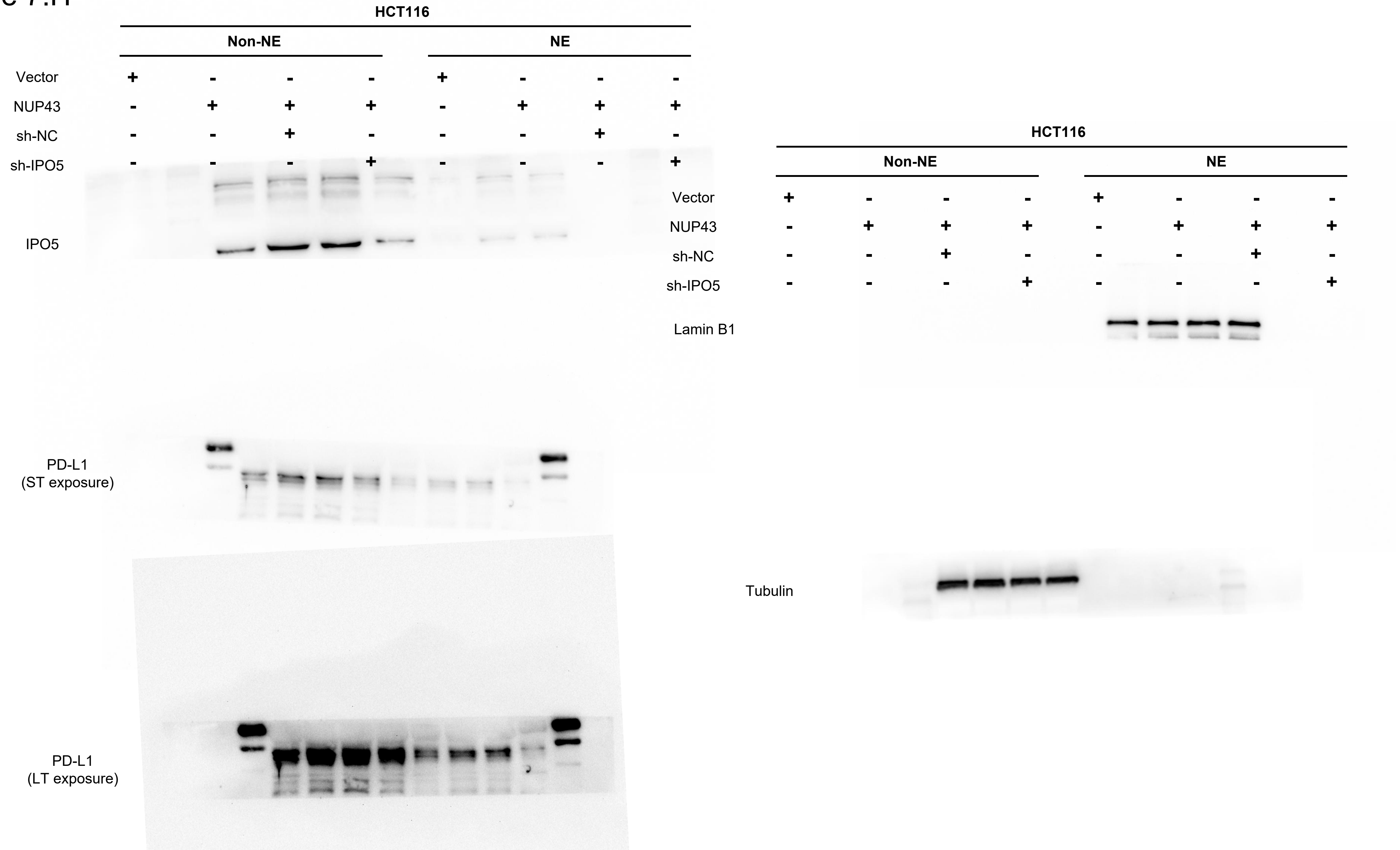
HCT116
Non-NE
NE
Vector
NUP43
sh-NC
sh-IPO5
IPO5
PD-L1
(ST exposure)
PD-L1
(LT exposure)
HCT116
Non-NE
NE
Vector
NUP43
sh-NC
sh-IPO5
Lamin B1
Tubulin

Figure 7.I

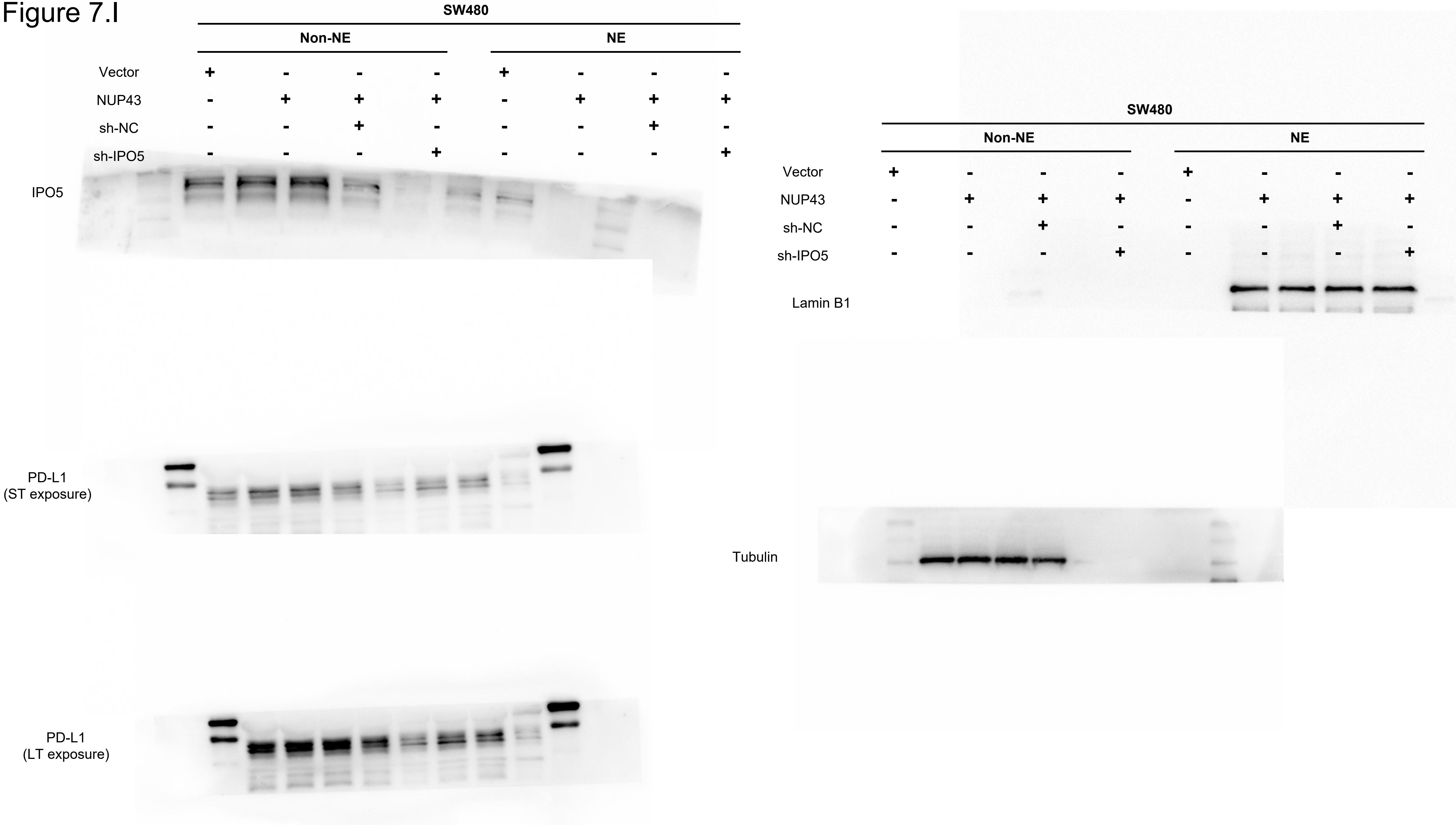
SW480
Non-NE
NE
Vector
NUP43
sh-NC
sh-IPO5
IPO5
PD-L1
(ST exposure)
PD-L1
(LT exposure)
SW480
Non-NE
NE
Vector
NUP43
sh-NC
sh-IPO5
Lamin B1
Tubulin

Figure 8.H&I

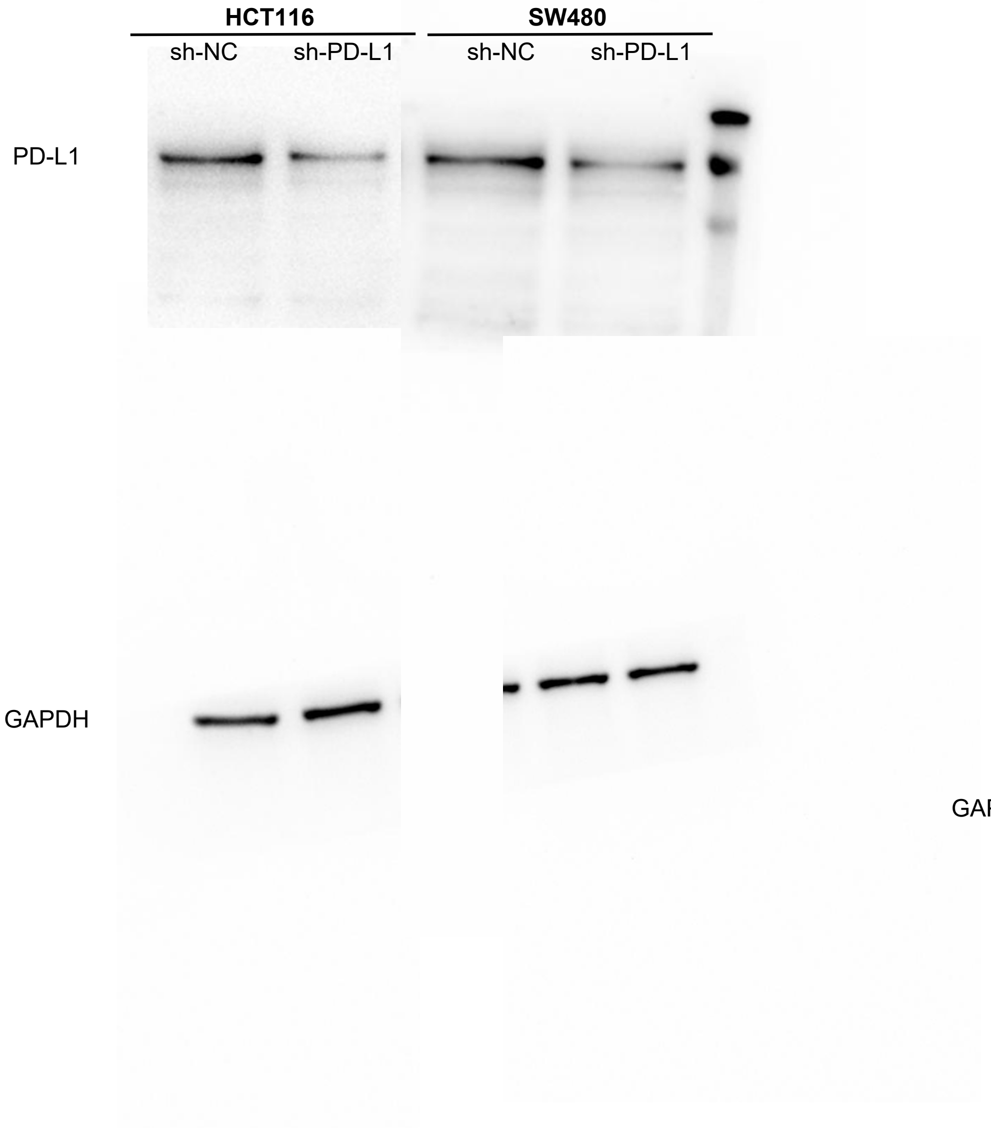
HCT116
SW480
sh-NC
sh-PD-L1
sh-NC
sh-PD-L1
PD-L1
GAPDH

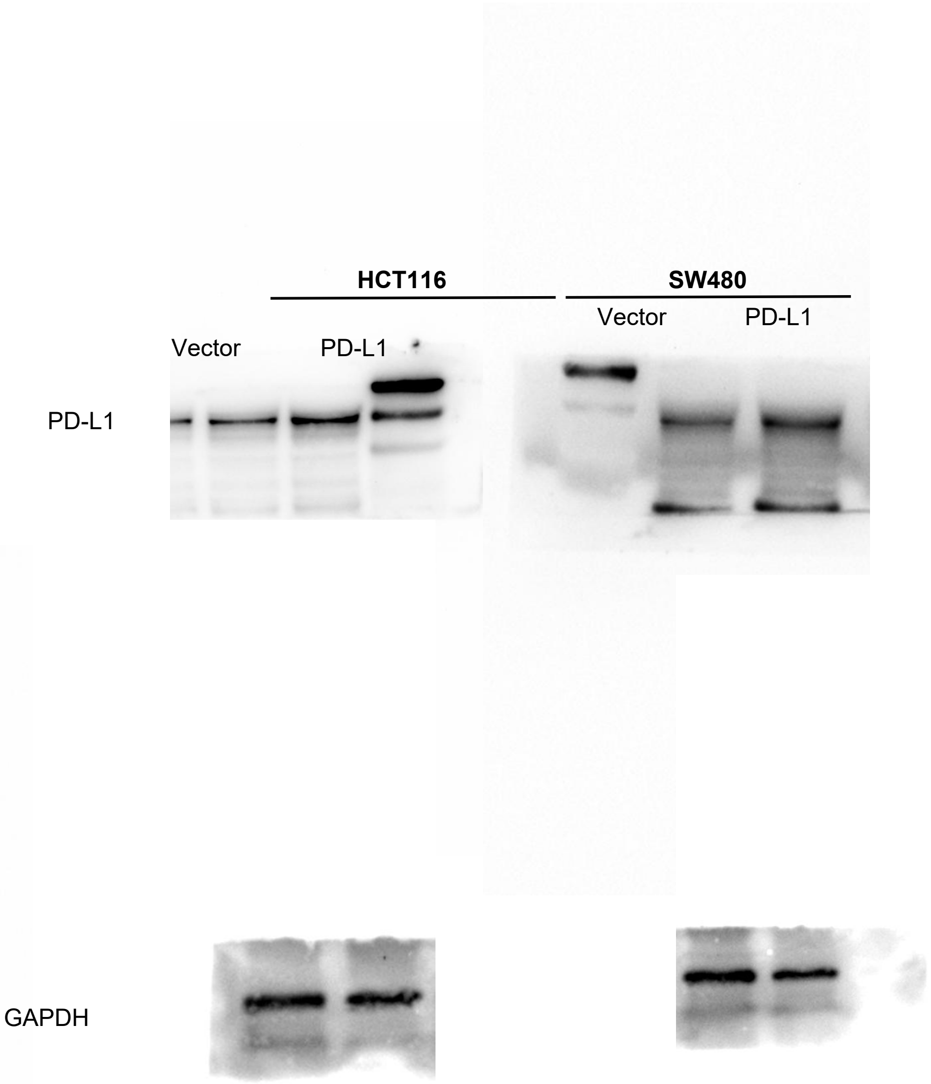
HCT116
SW480
Vector
PD-L1
Vector
PD-L1
PD-L1
GAPDH

Figure 8.J

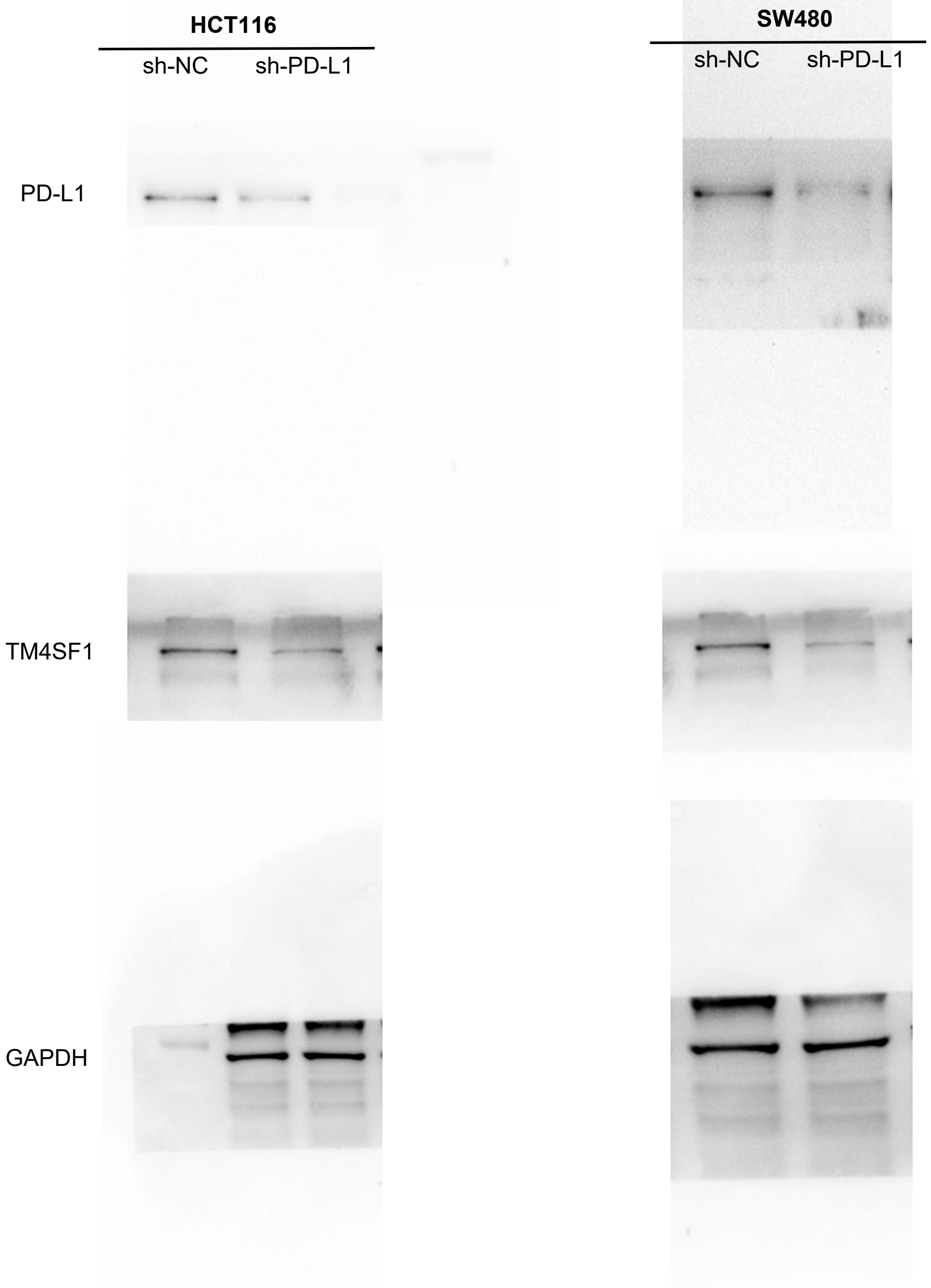

Figure 8.K

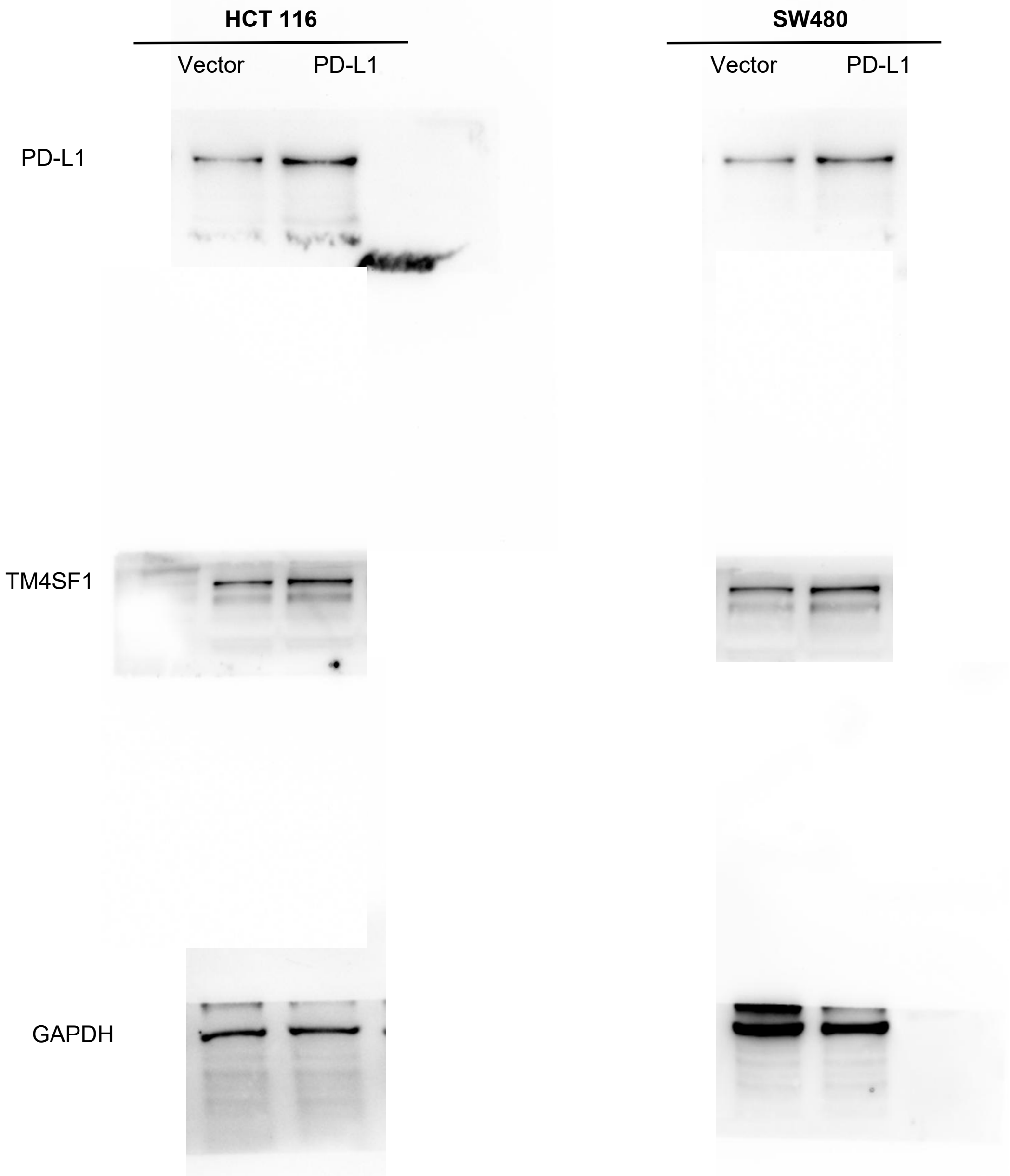

Figure 8. L

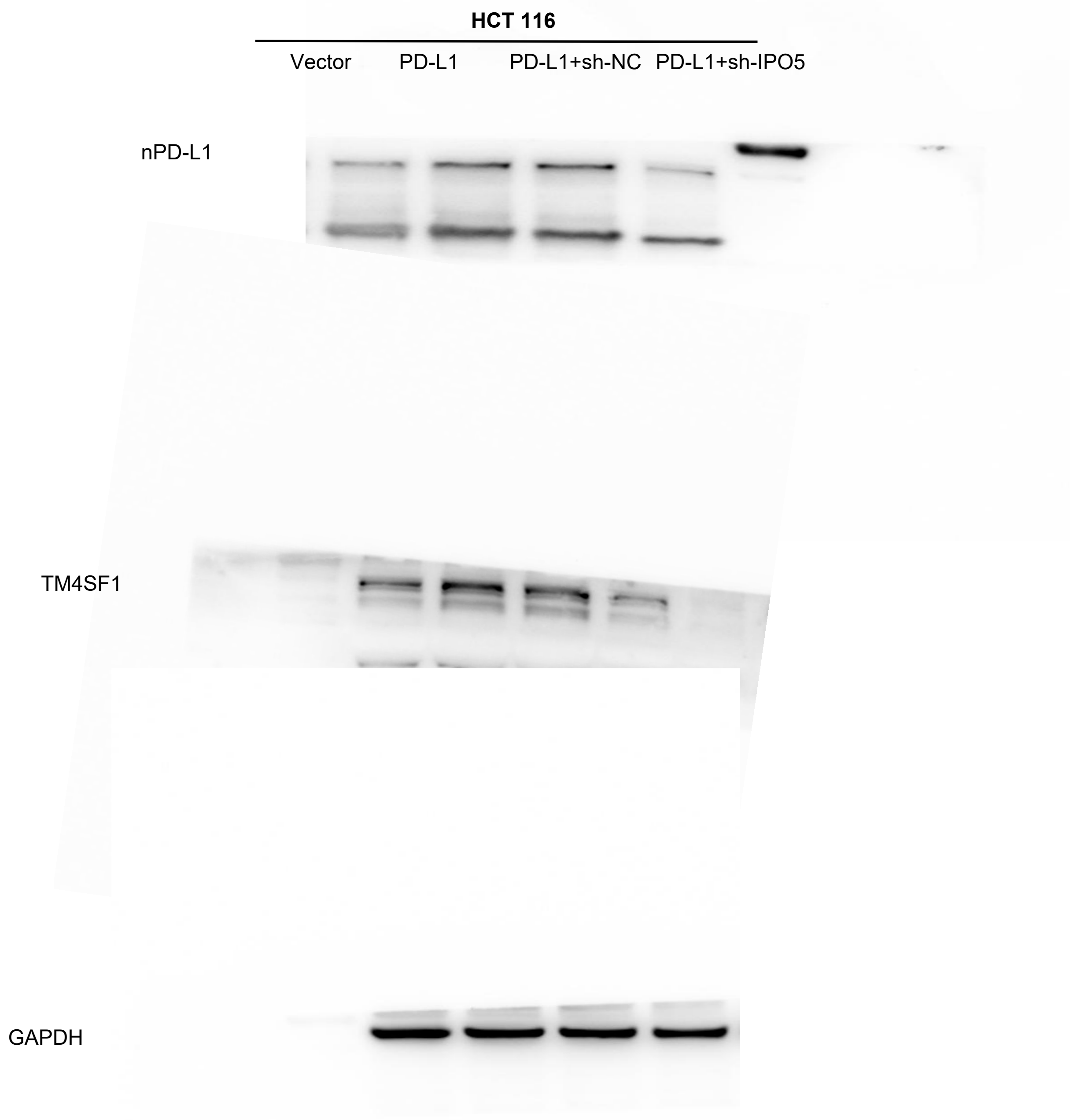

HCT 116
Vector
PD-L1
PD-L1+sh-NC
PD-L1+sh-IPO5
nPD-L1
TM4SF1
GAPDH

Figure 8.M

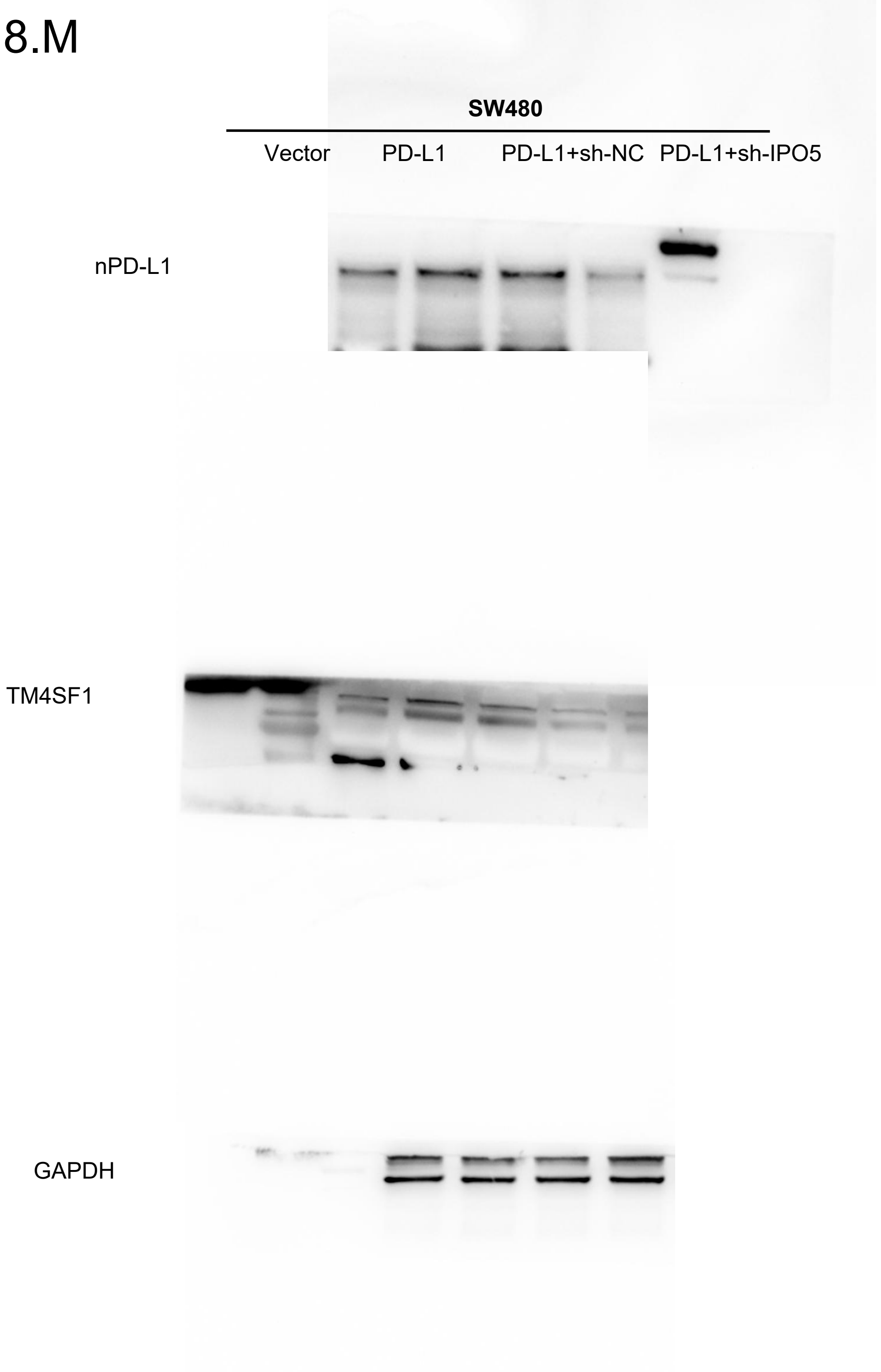

Figure 9.B

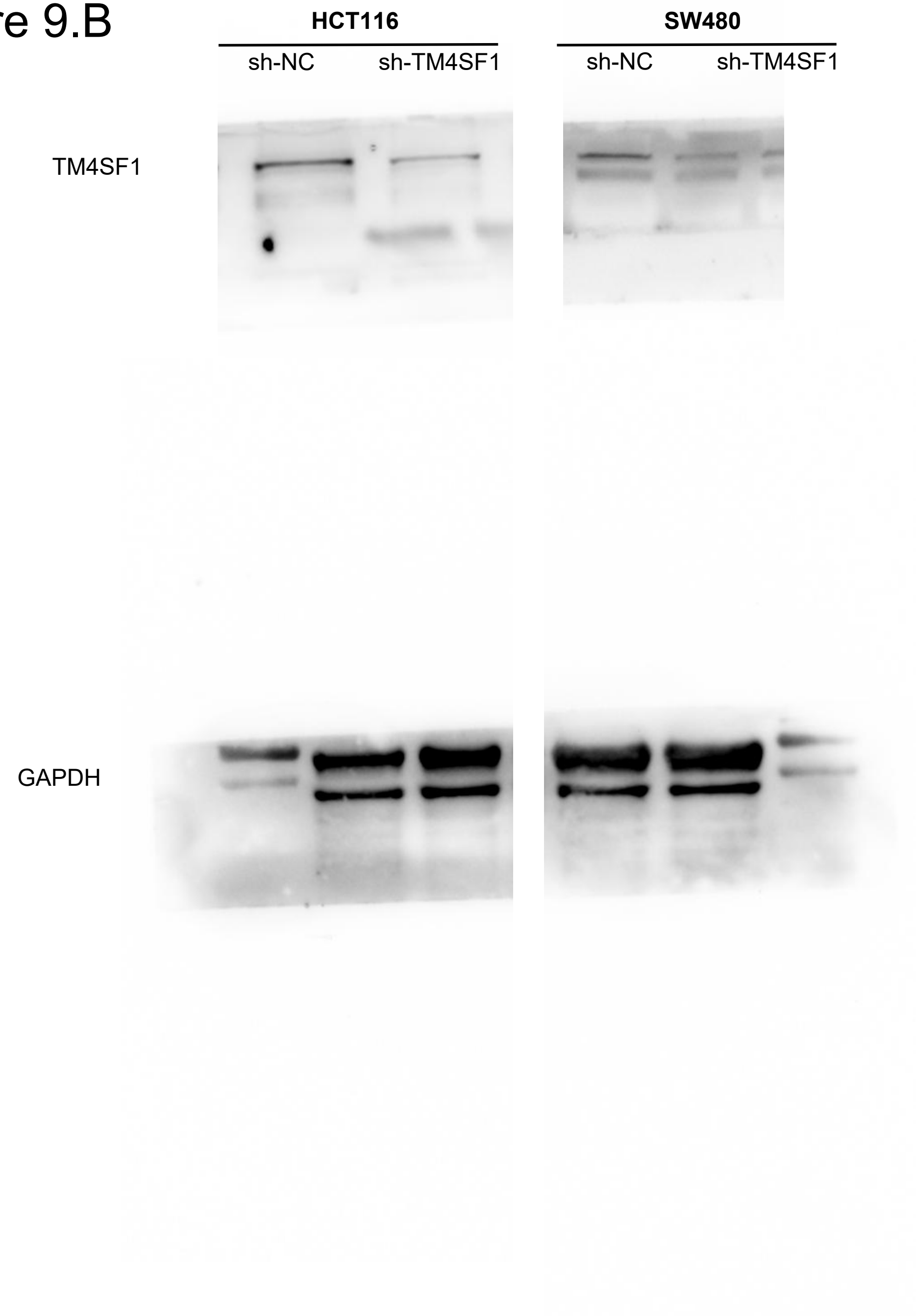

Figure 9.C

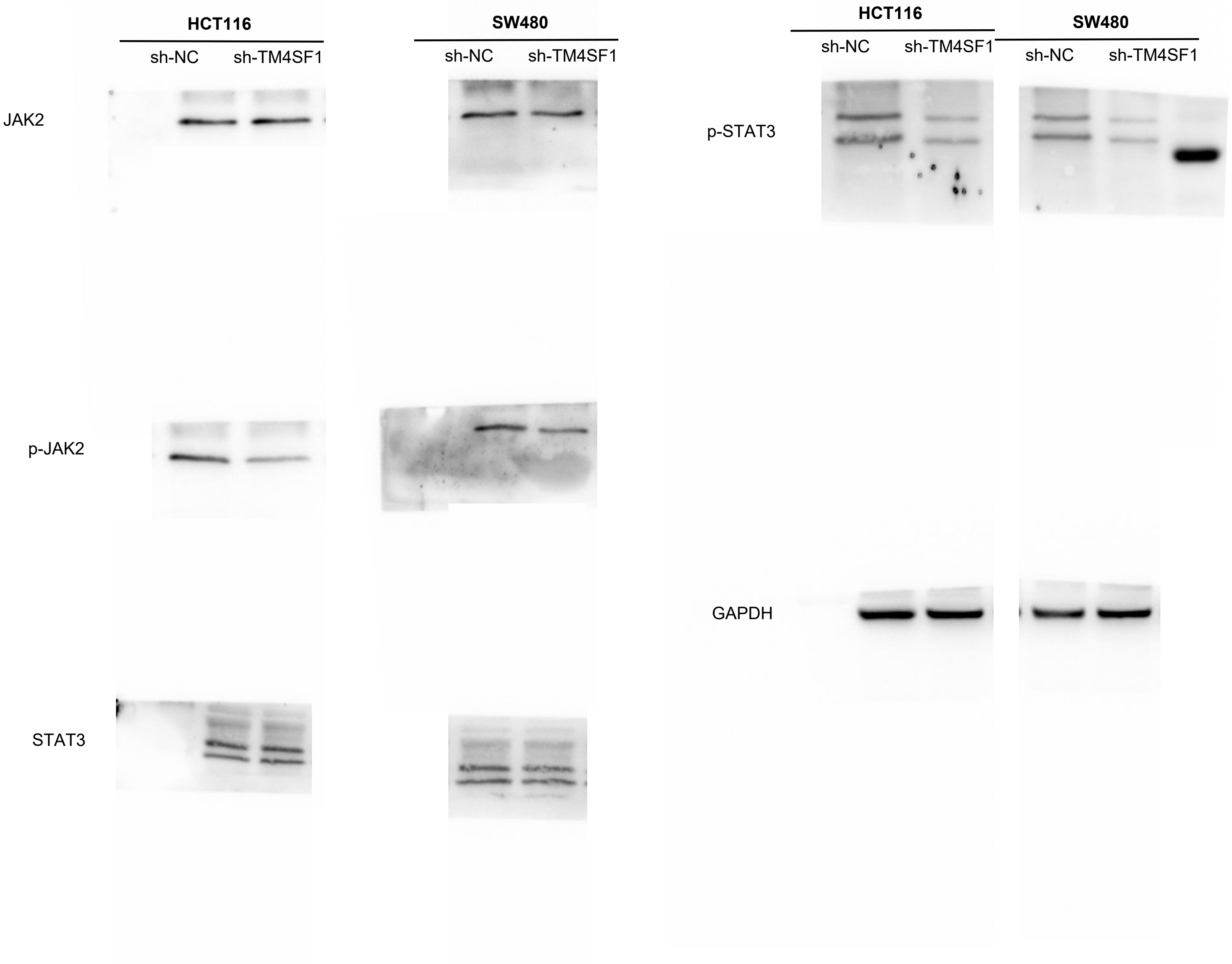
HCT116
sh-NC
sh-TM4SF1
SW480
sh-NC
sh-TM4SF1
JAK2
p-JAK2
STAT3
HCT116
sh-NC
sh-TM4SF1
SW480
sh-NC
sh-TM4SF1
p-STAT3
GAPDH

Figure 9.E

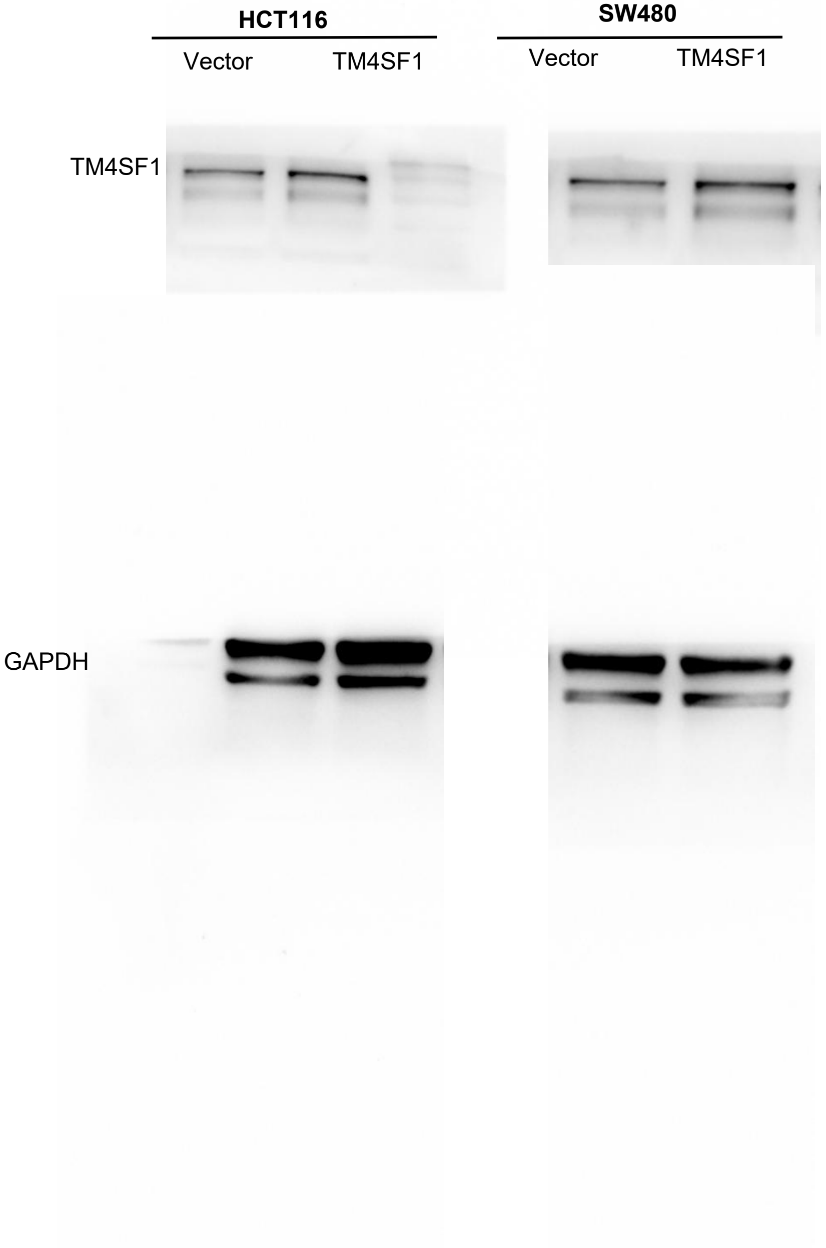

Figure 9.F

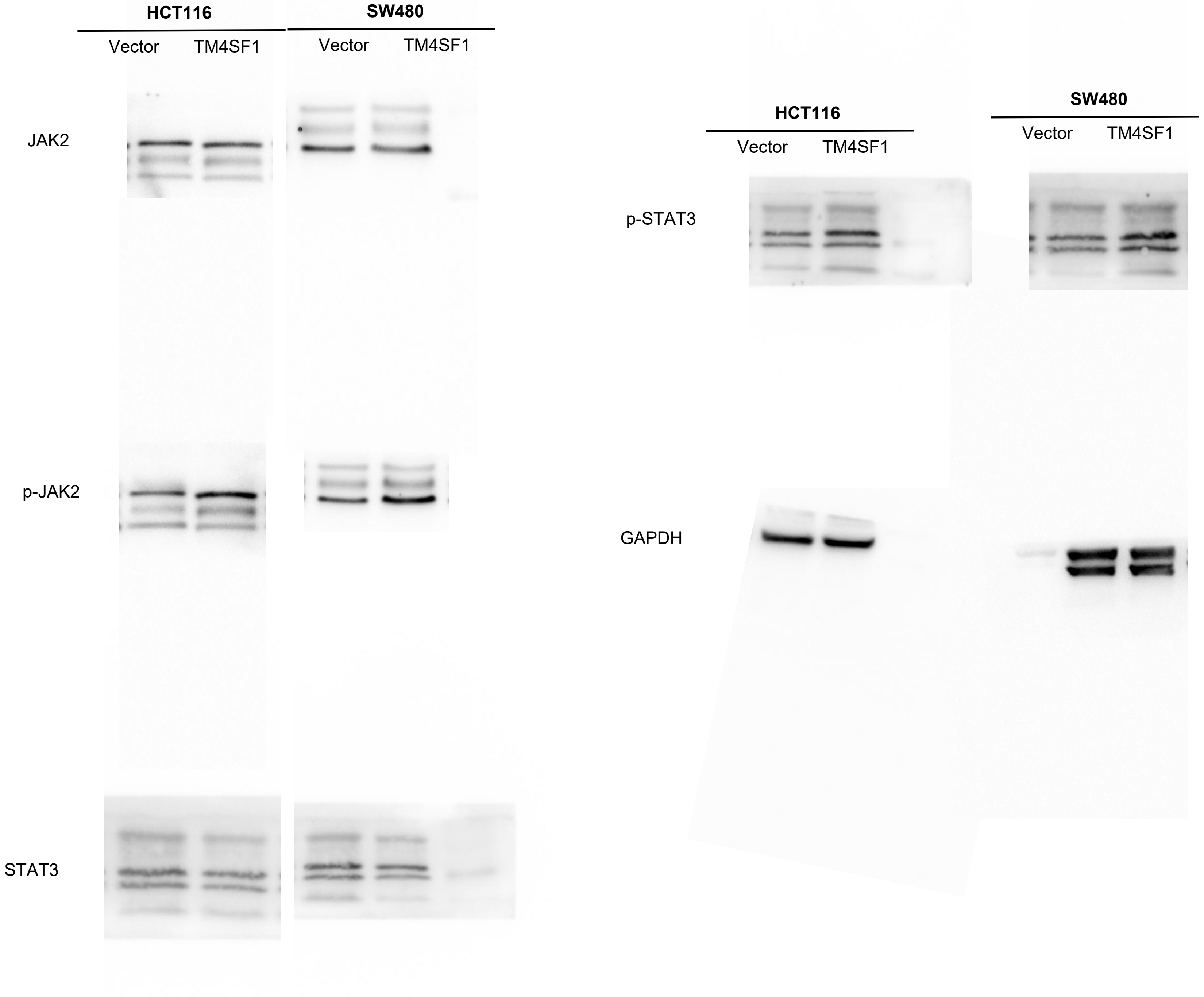
HCT116
SW480
Vector
TM4SF1
Vector
TM4SF1
JAK2
p-JAK2
STAT3
HCT116
SW480
Vector
TM4SF1
Vector
TM4SF1
p-STAT3
GAPDH

Figure 9.K

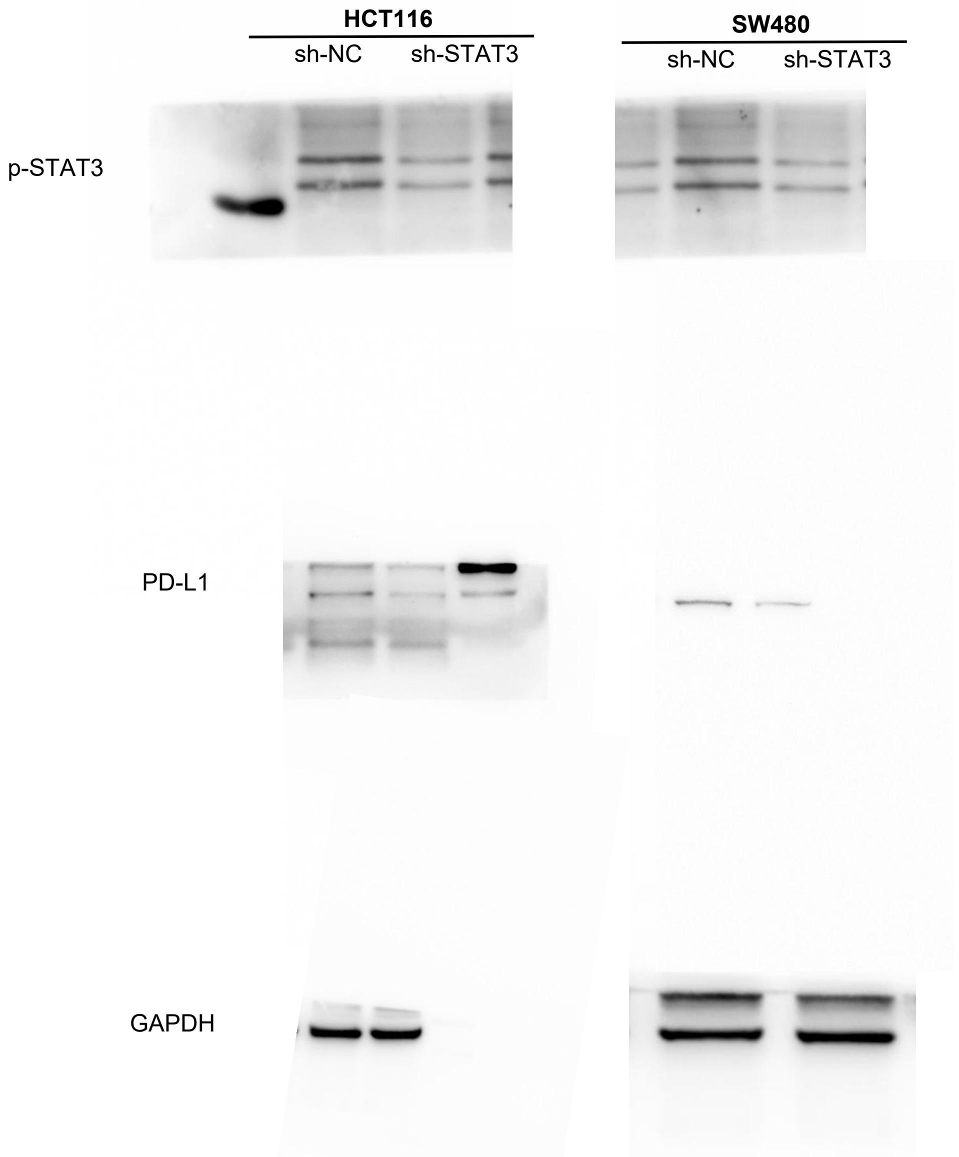

Supplement: Supplementary file 3 — wb&CO-IP RAW DATA [file 41420_2024_2025_MOESM3_ESM.pdf]
